# mTORC1 and CK2 coordinate ternary and eIF4F complex assembly

**DOI:** 10.1038/ncomms11127

**Published:** 2016-04-04

**Authors:** Valentina Gandin, Laia Masvidal, Marie Cargnello, Laszlo Gyenis, Shannon McLaughlan, Yutian Cai, Clara Tenkerian, Masahiro Morita, Preetika Balanathan, Olivier Jean-Jean, Vuk Stambolic, Matthias Trost, Luc Furic, Louise Larose, Antonis E. Koromilas, Katsura Asano, David Litchfield, Ola Larsson, Ivan Topisirovic

**Affiliations:** 1 Lady Davis Institute, SMBD JGH, McGill University, Montreal, Quebec, Canada H3T 1E2; 2 Department of Oncology, McGill University, Montreal, Quebec, Canada H3T 1E2; 3 Department of Biochemistry, McGill University, Montreal, Quebec, Canada H3T 1E2; 4 Department of Experimental Medicine, McGill University, Montreal, Quebec, Canada H3T 1E2; 5 Department of Oncology-Pathology, Science for Life Laboratory, Karolinska Institutet, Stockholm 171 76, Sweden; 6 Departments of Biochemistry and Oncology, Schulich School of Medicine & Dentistry, Western University, London, Ontario, Canada N6A 4L6; 7 Cancer Program, Biomedicine Discovery Institute and Department of Anatomy and Developmental Biology, Monash University, Victoria 3168, Australia; 8 UPMC Univ Paris 06, CNRS-UMR8256, Paris 75005, France; 9 Princess Margaret Cancer Centre, University Health Network, and the Department of Medical Biophysics, University of Toronto, Toronto, Ontario, Canada M5G 1L7; 10 MRC Protein Phosphorylation and Ubiquitylation Unit, University of Dundee, Scotland DD1 5EH, UK; 11 Department of Medicine, Polypeptide Laboratory, McGill University and The Research Institute of McGill University Health Centre, Montreal, Quebec, Canada H3A 2B2; 12 Molecular Cellular and Developmental Biology Program, Division of Biology, Kansas State University, Manhattan, Kansas 66506, USA

## Abstract

Ternary complex (TC) and eIF4F complex assembly are the two major rate-limiting steps in translation initiation regulated by eIF2α phosphorylation and the mTOR/4E-BP pathway, respectively. How TC and eIF4F assembly are coordinated, however, remains largely unknown. We show that mTOR suppresses translation of mRNAs activated under short-term stress wherein TC recycling is attenuated by eIF2α phosphorylation. During acute nutrient or growth factor stimulation, mTORC1 induces eIF2β phosphorylation and recruitment of NCK1 to eIF2, decreases eIF2α phosphorylation and bolsters TC recycling. Accordingly, eIF2β mediates the effect of mTORC1 on protein synthesis and proliferation. In addition, we demonstrate a formerly undocumented role for CK2 in regulation of translation initiation, whereby CK2 stimulates phosphorylation of eIF2β and simultaneously bolsters eIF4F complex assembly via the mTORC1/4E-BP pathway. These findings imply a previously unrecognized mode of translation regulation, whereby mTORC1 and CK2 coordinate TC and eIF4F complex assembly to stimulate cell proliferation.

## Abstract (web summary)

Ternary complex (TC) and eIF4F complex assembly are rate-limiting steps in translation initiation that are regulated by eIF2α phosphorylation and the mTOR/4E-BP pathway. Here the authors show that the protein kinases mTORC1 and CK2 coordinate TC and eIF4F complex assembly through eIF2β to stimulate cell proliferation.

## Graphical abstract

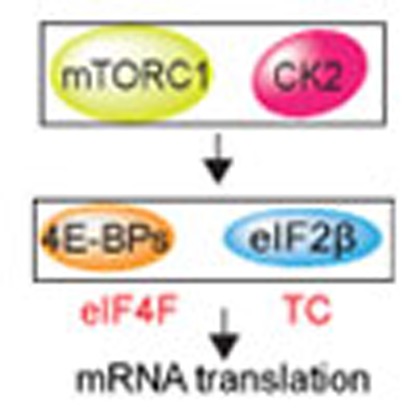

Messenger RNA (mRNA) translation plays a major role in homeostasis, whereas its dysregulation underpins a variety of pathological states including cancer, metabolic syndrome and neurological disorders^1^. Activation of mRNA translation requires rapid and highly coordinated assembly of the eukaryotic translation initiation factor 4F (eIF4F) complex composed of cap-binding subunit eIF4E, large scaffolding protein eIF4G and DEAD box helicase eIF4A, and the ternary complex (TC) comprised of eIF2, GTP and initiator tRNA (tRNA_i_^Met^)^2^. eIF4F recruits mRNA to the ribosome, whereas TC delivers tRNA_i_^Met^ (ref. 2). Mammalian/mechanistic target of rapamycin complex 1 (mTORC1) integrates a number of stimuli including nutrients, growth factors and hormones to bolster protein synthesis^3^. mTORC1 phosphorylates and inactivates the eIF4E-binding proteins (4E-BPs), which leads to their dissociation from eIF4E, thereby allowing eIF4E:eIF4G interaction and eIF4F complex assembly^1^. How mTORC1-dependent stimulation of eIF4F assembly is coordinated with TC recycling, however, remains largely underexplored.

eIF2 is a heterotrimer that comprises eIF2α, β and γ subunits^2^. After recognition of the start codon by tRNA_i_^Met^, eIF2-bound GTP is hydrolyzed to GDP and the TC complex is recycled by the guanine nucleotide exchange factor (GEF) eIF2B, which converts eIF2:GDP to eIF2:GTP^2^. eIF2α phosphorylation, which is induced by eIF2α kinases (protein kinase RNA-like endoplasmic reticulum kinase (PERK), protein kinase RNA-activated (PKR), general control nonderepressible 2 (GCN2) and haem-regulated inhibitor kinase) in response to various types of stress including endoplasmic reticulum stress, amino-acid unavailability, haem deficiency and viral infection, inhibits GEF function of eIF2B, thereby suppressing TC recycling and limiting TC levels^1^,^2^. This leads to suppression of global protein synthesis, with concomitant increase in translation of mRNAs harbouring inhibitory upstream open reading frames (uORF mRNAs) that encode stress-induced transcriptional regulators such as activating transcription factor 4 (ATF4) and CCAAT-enhancer-binding protein homologous protein (CHOP)^4^. Persistent mTORC1 activation is thought to induce chronic endoplasmic reticulum stress and perturbs AKT signalling, resulting in secondary elevation in eIF2 kinase activity and eIF2α phosphorylation^4^. However, it is largely unknown how mTORC1 affects eIF2α phosphorylation during acute activation of the translational machinery by nutrients, growth factors or hormones (for example, insulin).

## Results

### mTOR decreases phospho-eIF2α-stimulated translation

**Figure 1:**
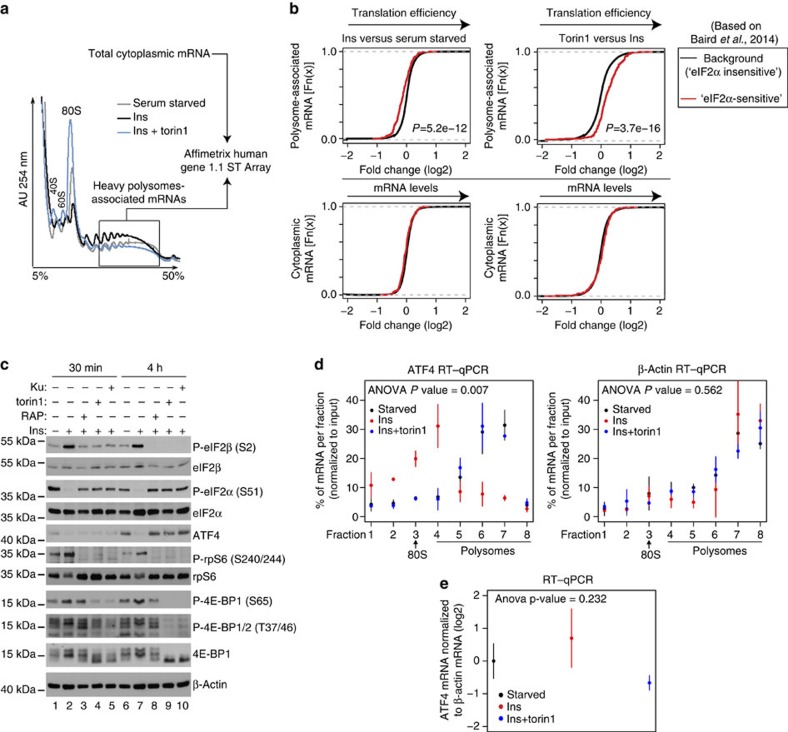
mTOR stimulates eIF2β phosphorylation, decreases eIF2α phosphorylation and represses translation of mRNAs that are upregulated by phospho-eIF2α. (**a**,**b**) MCF7 cells were serum starved for 16 h followed by 4-h stimulation with insulin (4.2 nM; Ins) in the presence of a vehicle (DMSO) or torin1 (250 nM). (**a**) Absorbance profiles (254 nm) of cytosolic extracts loaded onto 5–50% sucrose gradients and sedimented by ultracentrifugation. mRNA was isolated from fractions containing >3 ribosomes (box) and analysed in parallel with cytoplasmic mRNA using microarrays (see Methods). Positions of small (40S) and large (60S) ribosome subunits, monosomes (80S) and polysomes in the gradients are indicated. AU, absorbance units. (**b**) Transcriptome-wide effects of 4-h insulin and insulin+torin1 treatments on the translatome (polysome associated; upper panel) or steady-state cytoplasmic mRNA levels (lower panel). Presented are mRNAs whose translation is upregulated by eIF2α phosphorylation (‘eIF2α sensitive', red curve) and those that are not affected under conditions where eIF2α phosphorylation is stimulated (background; black curve) according to the study of Baird *et al*.^5^. Wilcoxon *P* values contrasting fold changes for eIF2α-regulated to background mRNAs are indicated. The experiment was carried out in four independent replicates. (**c**) MCF7 cells were treated as in **b** for the indicated time periods. In addition to torin1, allosteric mTOR inhibitor rapamycin (RAP; 50 nM) and active-site mTOR inhibitor KU-0063794 (KU; 3 μM) were used. Phosphorylation and expression levels of indicated proteins were monitored by western blotting. β-Actin served as a loading control. Experiments were repeated in at least two independent replicates and quantified by densitometry (Supplementary Fig. 9). (**d**,**e**) MCF7 cells were serum starved for 16 h (Starved) and then treated and fractionated as in **b**. Relative amounts of ATF4 and β-actin mRNA in polysome fractions (**d**) or cytosolic extracts (for steady-state mRNA measurements) (**e**) were determined by reverse transcription–quantitative PCR (RT–qPCR). Position of monosome (80) and polysomal fractions are shown. (**d**,**e**) S.d.'s and interaction (treatment and fraction) *P* values from a two-way analysis of variance (ANOVA) using means of two independent experiments each consisting of technical replicates are indicated.

Recently, a transcriptome-wide catalogue of mRNAs whose translation is upregulated after induction of eIF2α phosphorylation by acute endoplasmic reticulum stress (hereafter referred to as ‘eIF2α-sensitive' mRNAs) was determined^5^. To investigate the effects of changes in mTOR signalling on translation of ‘eIF2α-sensitive' mRNAs^5^, we used the polysome profiling technique, wherein mRNAs are separated based on the numbers of ribosomes they bind using a sucrose gradient and ultracentrifugation, followed by analysis of the changes in translation and cytosolic mRNA levels on a transcriptome-wide scale^6^. Transcriptome-wide polysome profiling in MCF7 cells revealed that induction of mTOR signalling by 4 h insulin treatment coincides with translational suppression of ‘eIF2α-sensitive' mRNAs^5^, as compared with those whose translation was determined to be independent of eIF2α phosphorylation^5^ (background mRNAs; Fig. 1a,b; *P*<5.2e**−**12). In turn, addition of the active-site mTOR inhibitor torin1 (ref. 7) to insulin-treated cells selectively induced translation of ‘eIF2α-sensitive' mRNAs (Fig. 1a,b; *P*<3.7e**−**16). These effects of modulation of mTOR signalling on translation of ‘eIF2α-sensitive' mRNAs were not caused by the changes in cytoplasmic mRNA levels (Fig. 1b).

### mTOR affects phospho-eIF2β and phospho-eIF2α levels

**Figure 2:**
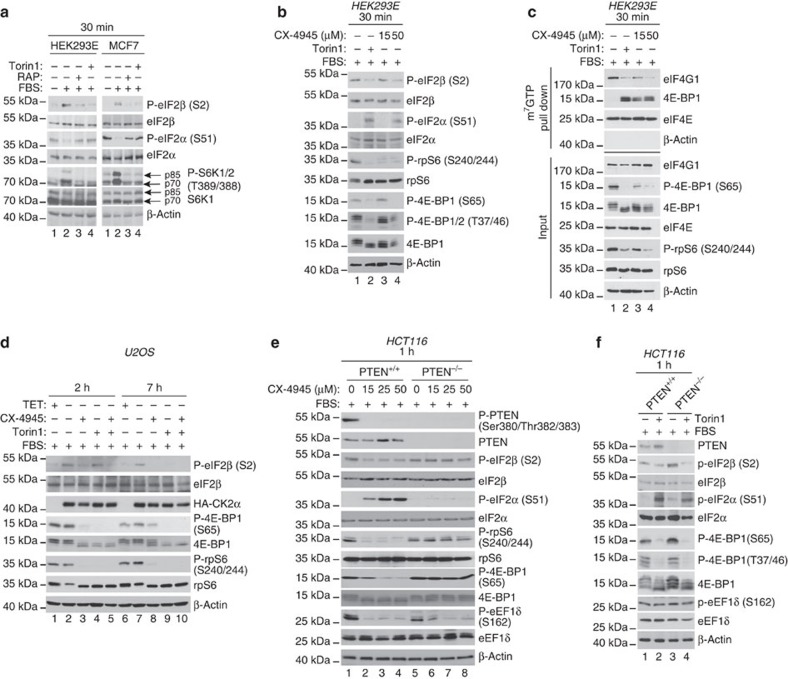
mTOR and CK2 inhibitors suppress eIF2β phosphorylation, increase phospho-eIF2α levels and interfere with eIF4F complex assembly. (**a**) HEK293E or MCF7 cells were serum starved for 16 h and then stimulated with 10% serum (fetal bovine serum, FBS) in the presence of a vehicle (DMSO), rapamycin (50 nM) or torin1 (250 nM) for 30 min. Phosphorylation status and levels of indicated proteins were monitored by western blotting. β-Actin served as a loading control. (**b**) HEK293E cells were serum starved for 16 h and then stimulated for 30 min with serum (FBS; 10%) in the presence of a vehicle (DMSO), 250 nM torin1, 15 or 50 μM CX-4945. Phosphorylation and expression levels of indicated proteins were monitored by western blotting. β-Actin served as a loading control. (**c**) Extracts of HEK293E cells treated as in **b** were subjected to m^7^GTP cap pull down (see Methods). Levels and phosphorylation status of indicated proteins in the pulled down material (25%) and inputs (10%) were determined by western blotting. β-Actin served to exclude contamination of m^7^GTP cap pull downs (for example, non-specific binding to the agarose beads) and as a loading control for inputs. Parallel results were obtained in MCF7 and HCT116 cells (Supplementary Fig. 1a,b). (**d**) Expression of HA-CK2α in U2OS cells was induced by tetracycline withdrawal (doxycycline was used at 1.5 μg ml^−1^ to suppress expression of CK2 subunits in control cells). Cells were starved for 16 h after which cells were stimulated by 10% serum (FBS) in the presence of a vehicle (DMSO), 250 nM torin1, 50 μM CX-4945 or a combination thereof for the indicated time period. (**e**) HCT116 PTEN^+/+^ or PTEN^−/−^ cells were treated with the indicated concentration of CX-4945 for 1 h in the presence of 10% serum (FBS). (**f**) Cells described in **e** were incubated with DMSO or torin1 (250 nM) for 1 h. (**d**–**f**) Expression levels and phosphorylation status of indicated proteins were monitored by western blotting. β-Actin served as a loading control. Experiments in this panel were repeated at least two times independently and the representative results are shown. Where appropriate, quantification was performed using densitometry (Supplementary Fig. 9).

Insulin induced mTORC1 signalling as illustrated by elevated phosphorylation of 4E-BPs and the S6 kinase (S6K) substrate ribosomal protein S6 (rpS6) as compared with control serum-starved cells, which was reverted by the allosteric mTOR inhibitor rapamycin or active-site mTOR inhibitor (KU-0063794 and torin1) (Fig. 1c; compare lanes 2 and 7 with lanes 3–5 and 8–10, respectively). In addition, insulin decreased phospho-eIF2α levels as compared with control serum-starved cells, and cells stimulated with insulin in the presence of mTOR inhibitors (Fig. 1c; compare lanes 2 and 7 with lanes 3–5 and 8–10, respectively). In stark contrast to effects of treatments on eIF2α phosphorylation, eIF2β phosphorylation on serine 2 (Ser2) was enhanced by insulin relative to the control, and diminished when cells were stimulated with insulin in the presence of mTOR inhibitors (Fig. 1c; compare lanes 2 and 7 with lanes 3–5 and 8–10, respectively). Phosphorylation of eIF2α stimulates ATF4 mRNA translation and consequently upregulates ATF4 protein levels^8^. Although the effects of insulin and mTOR inhibitors on eIF2α phosphorylation were detected after 30 min, neither insulin nor mTOR inhibitors exerted a major impact on ATF4 protein expression at this time point (Fig. 1c; compare lane 1 with lanes 2–5). Even though these results appear surprising, they are consistent with several studies showing that under a number of experimental conditions that favour eIF2α phosphorylation including ultraviolet irradiation, endoplasmic reticulum and osmotic stress, induction of ATF4 protein expression is either delayed or absent^9^,^10^,^11^. Accordingly, insulin downregulated ATF4 protein levels relative to the control, which was reversed by mTOR inhibitors after 4 h (Fig. 1c; lanes 6–10). Under these conditions, insulin inhibited ATF4 mRNA translation as illustrated by the shift of ATF4 mRNA towards lighter polysomes, as compared with control serum-starved cells or cells that were stimulated with insulin in the presence of torin1 (Fig. 1d, left panel). In contrast, distribution of β-actin mRNA across the polysomes was essentially unchanged between the treatments (Fig. 1d, right panel). These findings show that under these conditions, mTOR activation leads to inhibition of ATF4, but not β-actin mRNA translation, which is consistent with results obtained from the transcriptome-wide polysome-profiling study (Fig. 1b). Therefore, similarly to the establish selectivity of the stimulatory effects of mTOR on translation of terminal oligopyrimidine (TOP) and long and structured 5′-untranslated region (UTR) harbouring mRNAs^12^, mTOR also appears to selectively inhibit translation of mRNAs that are upregulated under conditions wherein eIF2α phosphorylation is increased. Although insulin slightly induced steady-state ATF4 mRNA levels compared with torin1, these effects were not significant (Fig. 1e), thereby indicating that the changes in ATF4 protein expression observed between treatments are largely caused by the changes in translation and not by the upstream steps in the gene expression pathway (for example, transcription or mRNA stability). Therefore, short-term (4 h) inhibition of mTOR signalling correlates with reduction in phospho-eIF2β levels, increase in phospho-eIF2α levels and concomitant translational activation of ‘eIF2α-sensitive' mRNAs including ATF4. Since the latter findings were based on a single-cell line, we tested the effects of serum withdrawal and repletion on phosphorylation of eIF2β and eIF2α in the presence of mTOR inhibitors in parallel in HEK293E and MCF7 cells. A 30-min serum stimulation induced mTOR activity in HEK293E and MCF7 cells as demonstrated by increased S6K phosphorylation, which coincided with increased eIF2β phosphorylation and reduced phospho-eIF2α levels relative to the control (Fig. 2a; compare lanes 1 and 2). These effects were largely diminished when cells were stimulated with serum in the presence of rapamycin or torin1 (Fig. 2a; compare lane 2 versus lanes 3–4). Therefore, observed correlation between changes in mTOR activity and phosphorylation status of eIF2α and eIF2β is not limited to a single-cell line.

### CK2 phosphorylates eIF2β and stimulates eIF4F assembly

It has been reported that Casein kinase 2 (CK2), which is stimulated by insulin and serum^13^,^14^ phosphorylates eIF2β on serines 2 and 67 (Ser2 and Ser67), and eIF2β phosphorylation at these sites may stimulate global mRNA translation and cell proliferation^15^. We therefore investigated whether CK2 plays a role in the apparent correlation between induction of eIF2β phosphorylation and stimulation of mTOR signalling by serum and insulin. Starved HEK293E cells were serum stimulated in the presence of a vehicle (dimethylsulphoxide, DMSO), torin1 or CK2 inhibitor CX-4945 for 30 min. Both torin1 and CX-4945 reduced serum-induced phosphorylation of eIF2β in HEK293E cells, which was paralleled by increased eIF2α phosphorylation, relative to the control (Fig. 2b; compare lane 1 with lanes 2–4). In addition, both the inhibitors reduced 4E-BP1 phosphorylation (Fig. 2b; compare lane 1 with lanes 2–4), although higher concentration of CX-4945 (50 μM) was required for strong inhibition of eIF2β and 4E-BP1 phosphorylation and induction of eIF2α phosphorylation as compared with rpS6 phosphorylation (15 μM) (Fig. 2b; lane 3 versus 4). Consistent with inhibiting 4E-BP1 phosphorylation, CX-4945 impaired eIF4F assembly as illustrated by reduced eIF4E:eIF4G1 association and increased eIF4E:4E-BP1 binding relative to the control, and this effect was more pronounced when a higher concentration of CX-4945 (that is, 50 μM) was used (Fig. 2c, compare lane 1 with lanes 2–4). Equivalent results were observed in MCF7 cells, whereby torin1 and CX-4945 (15 and 50 μM) reduced eIF2β phosphorylation and disrupted eIF4F complex (Supplementary Fig. 1a). Moreover, similar effects of 50 μM CX-4945 on the mTORC1 signalling and eIF4F complex assembly were observed in HCT116 cells (Supplementary Fig. 1b). Collectively, these findings demonstrate that CK2 regulates not only eIF2β phosphorylation but also eIF4F complex assembly. Next, we sought to determine the relationship between CK2 and mTOR in regulating eIF2β phosphorylation and eIF4F complex assembly. We first employed U2OS cells in which CK2α-HA/Myc-β expression and consequently CK2 activity are increased by removal of doxycycline from the growth media^16^ (Fig. 2d). Induction of CK2α-HA/Myc-β expression increased eIF2β phosphorylation, while exhibiting minimal effects on 4E-BP1 and rpS6 phosphorylation, which was expected as these experiments were carried out under full serum conditions wherein mTORC1 activity is high (Fig. 2d; compare lane 1 versus 2 and 6 versus 7). In turn, CX-4945 decreased phosphorylation of eIF2β as well as 4E-BP1 and rpS6 after 2 and 7 h (Fig. 2d; compare lane 2 versus 3 and 7 versus 8). Increase in CK2 activity engendered by overexpression of its subunits did not conspicuously affect the ability of torin1 to inhibit phosphorylation of 4E-BP1 and rpS6 (Fig. 2d; compare lane 2 versus 4 and 7 versus 9). In contrast, the effects of torin1 on eIF2β phosphorylation were attenuated in cells in which CK2α-HA/Myc-β overexpression was induced, whereby torin1 inhibited eIF2β phosphorylation after 7 h but not after 2 h (Fig. 2d; compare lane 2 versus 4 and 7 versus 9). These findings suggest that CK2 directly phosphorylates eIF2β, while stimulating 4E-BP1 phosphorylation via mTORC1, whereby the effects of mTOR inhibitors on eIF2β phosphorylation are mitigated by CK2α-HA/Myc-β overexpression.

### mTORC1 phosphorylates eIF2β independently of CK2

**Figure 3:**
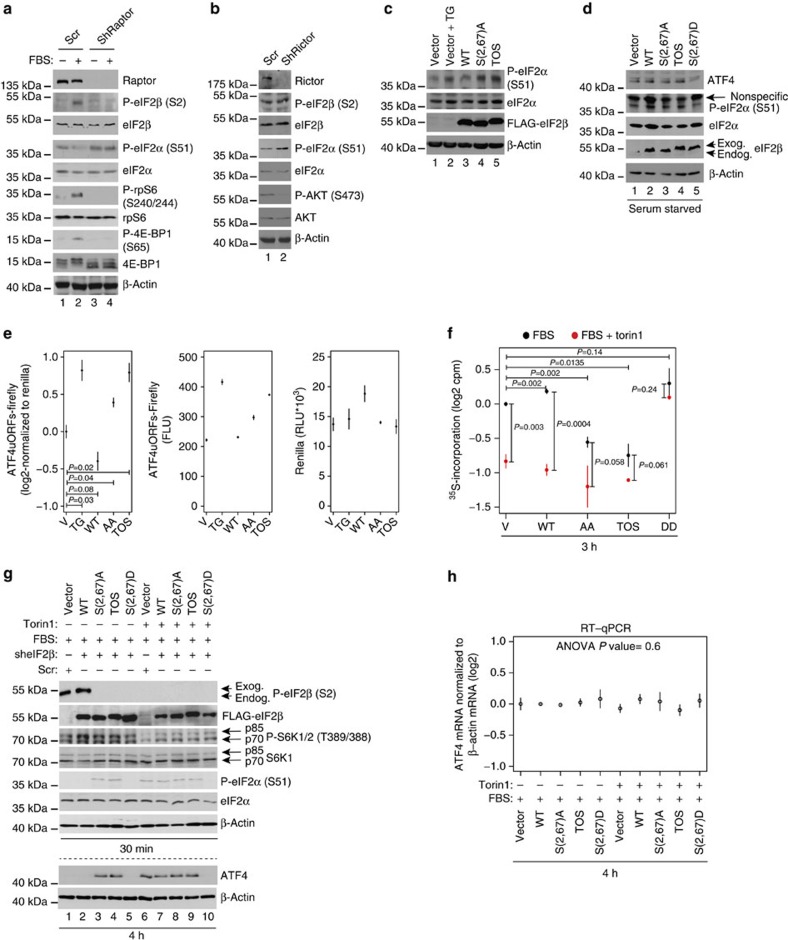
eIF2β phosphorylation by mTORC1 coincides with dephosphorylation of eIF2α. HEK293E cells infected with scrambled shRNA (Scr) (**a**,**b**), raptor (**a**) or rictor (**b**) shRNA were serum starved (16 h) and then stimulated with 10% serum (FBS; 30 min). (**c**,**d**) HEK293E cells were co-transfected with shown FLAG-eIF2β variants, p5′UTR ATF4-firefly (ATF4 uORFs) and renilla luciferase reporter constructs and treated with 1 μM thapsigargin (TG; 1 h) (**c**) or serum starved (**d**). (**e**) Luciferase activity in cells described in **c** and **d** was determined by chemiluminescence. Mean relative light units (RLU) of individual luciferase (middle and right panel) or ratio thereof (left panel) are shown. Data of three independent experiments were log2 transformed, normalized per replicate and to the mean of control condition, and shown as means and s.d.'s. *P* values from one-way analysis of variance (ANOVA) are indicated. (**f**) HEK293E cells were serum starved (16 h) and followed by 10% serum stimulation (FBS) in the presence of a vehicle (DMSO) or torin1 (250 nM) for 3 h. Global protein synthesis was monitored by ^35^S-Met/Cys incorporation. Data from three independent experiments were log2 transformed, normalized per replicate and to the mean of the control condition, and shown as means±s.d. *P* values from one-way ANOVAs are indicated. (**g**) HEK293E cells infected with empty vector+scrambled shRNA (control) or stably expressing indicated eIF2β variants and depleted of endogenous eIF2β (Supplementary Fig. 4b; Methods) were serum starved (16 h), followed by 10% serum (FBS) stimulation in the presence of a vehicle (DMSO) or torin1 (250 nM) for 30 min or 4 h. (**h**) Steady-state ATF4 mRNA levels after 4 h treatments as described in **g** were determined by RT–qPCR and normalized over β-actin values. Means from two independent experiments, each in technical triplicate were log2 transformed, normalized per replicate and to the control, and are shown as means with s.d.'s. The *P* value from a one-way ANOVA across all treatments is indicated. (**a**–**d**,**g**) Experiments were repeated at least two times independently and representative results are shown. (**a**–**d**,**g**) Levels and phosphorylation status of indicated proteins were monitored by western blotting. β-Actin was a loading control. Densitometry is shown in Supplementary Fig. 9. Endog, endogenous; exog, exogenous.

CK2 phosphorylates PTEN resulting in its inactivation and consequent upregulation of mTOR activity^17^,^18^. Therefore, to further assess the hierarchy of mTORC1 and CK2 in regulation of eIF2β phosphorylation, isogenic HCT116 PTEN^+/+^ and PTEN^−/−^ cells^19^ were treated with CX-4945 and torin1. Although the primary CK2 phospho-acceptor sites on PTEN were mapped to Ser370/385 (ref. 17), due to limited availability of corresponding antibodies, we monitored the effects of CX-4945 on phosphorylation of Ser380/Thr382/383, which are also CK2 responsive^20^. In PTEN^+/+^ cells, CX-4945 strongly reduced PTEN phosphorylation and mTOR signalling as judged by reduction in rpS6 and 4E-BP1 phosphorylation (Fig. 2e; compare lane 1 with lanes 2–4), whereas in PTEN^−/−^ cells, there was no apparent effect of CX-4945 on mTOR signalling (Fig. 2e; compare lane 5 with lanes 6–8). Whereas 1-h CX-4945 treatment reduced eIF2β phosphorylation and induced phospho-eIF2α levels in a dose-dependent manner in PTEN^+/+^ cells, these effects of CX-4945 were largely absent in PTEN^−/−^ cells (Fig. 2e; compare lanes 2–4 with 6–8). CX-4945 however suppressed eIF2β phosphorylation and increased phospho-eIF2α levels after 7 h, which was not accompanied by a reduction in mTOR signalling (Supplementary Fig. 1c). In contrast to eIF2β phosphorylation, PTEN status did not have a major impact on the effects of CX-4945 on another bona fide CK2 substrate eEF1δ inasmuch as CX-4945 inhibited phosphorylation of eEF1δ to a comparable extent in both PTEN^−/−^ and PTEN^+/+^ cells after 1 h (Fig. 2e; compare lanes 2–4 to 6–8). Conversely, torin1, which inhibits mTORC1 downstream of PTEN^21^, reduced phosphorylation of 4E-BP1 and rpS6, irrespective of the PTEN status in the cell (Fig. 2f; lanes 2 and 4 versus lanes 1 and 3). Moreover, in both cell lines, eIF2β phosphorylation was suppressed to a comparable extent and this coincided with increased eIF2α phosphorylation (Fig. 2f; lanes 2 and 4 versus lanes 1 and 3). In turn, torin1 did not elicit a major effect on the phosphorylation of eEF1δ (Fig. 2f; lanes 1–4). These findings indicate that in the absence of mTOR inhibition, the effects of CK2 on eIF2β are delayed. To further establish the role of mTOR signalling in phosphorylation of eIF2β, we used TSC2^−/−^ mouse embryonic fibroblasts (MEFs) wherein mTOR is activated by the loss of a negative upstream regulator TSC1/2 (ref. 22). Similarly to cells lacking PTEN, torin1, but not CX-4945, inhibited eIF2β phosphorylation on Ser2 (Supplementary Fig. 1d,e). These data suggest that analogous to the attenuation of the effects of mTOR inhibition on eIF2β phosphorylation observed in U2OS cells overexpressing CK2 (Fig. 2d), lack of ability of CX-4945 to suppress mTOR mitigates its inhibitory effects on eIF2β phosphorylation (Fig. 2e; Supplementary Fig. 1e). These results suggest that CK2 and mTOR may collaboratively regulate eIF2β phosphorylation on Ser2. Inability of CK2 inhibitors to downregulate mTOR signalling in PTEN^−/−^ and TSC^−/−^ cells appears to coincide with delayed inhibition of eIF2β phosphorylation. Moreover, in TSC^−/−^ MEFs, in which serum starvation does not result in a marked mTOR inhibition, removal of serum had much lesser effect on eIF2β phosphorylation as compared with TSC^+/+^ MEFs (Supplementary Fig. 1f). This shows that efficient suppression of eIF2β phosphorylation in response to CK2 inhibitors and serum withdrawal requires downregulation of mTOR signalling, thereby suggesting that at least under certain conditions mTOR may phosphorylate eIF2β independently of CK2. Although sequences surrounding Ser2 and Ser67 in eIF2β do not correspond to a presumed mTOR consensus^23^, eIF2β contains a putative TOS motif (Supplementary Fig. 2a,b). The TOS motif is required for binding to raptor and recruitment of mTORC1 substrates^24^. Indeed, mTORC1 phosphorylated eIF2β in *in vitro* kinase assay, and this effect was reduced when mTORC1 was inhibited (by serum starvation or torin1 treatment of cells from which mTORC1 was isolated), when Ser2 and Ser67 were substituted with alanines (eIF2β S(2,67)A), or when TOS motif was disrupted (phenylalanine 89 substituted with alanine; TOS mutant) (Supplementary Fig. 2c–e). Reduced phosphorylation of eIF2β mutants did not stem from the inadvertent effects of mutations on the secondary structure of the protein as illustrated by indistinguishable far-ultraviolet circular dichroism spectra of wild-type (WT) and mutant eIF2β proteins (Supplementary Fig. 2f). CK2 was not detected in the mTORC1 complex used in *in vitro* kinase assay (Supplementary Fig. 2g). This excluded the possibility that eIF2β was phosphorylated due to the contamination of mTORC1 *in vitro* kinase assay reactions with CK2. In HEK293E cells, eIF2β co-immunoprecipitated with raptor when mTOR was activated by serum stimulation (Supplementary Fig. 2h). Disruption of putative eIF2β TOS motif abolished raptor:eIF2β interaction (Supplementary Fig. 2i), but not eIF2β:CK2 association (Supplementary Fig. 2j). Notably, although the indistinguishable amounts of CK2α were immunoprecipitated by TOS mutant and other eIF2β variants, TOS mutant was not phosphorylated in HEK293E cells (see below and Fig. 3g; lane 4, Supplementary Fig. 4c). Taken together, these findings indicate that at least under certain conditions mTORC1 phosphorylates eIF2β independently of CK2. mTOR exists in two complexes, mTORC1 and mTORC2 that differ in their function and composition^3^. mTORC1 integrates signals from nutrients, hormones and growth factors (including insulin) to induce anabolic processes including protein synthesis, whereas mTORC2 regulates AGC kinases including AKT and has been implicated in cytoskeleton maintenance and co-translational protein degradation^3^. To confirm that eIF2β phosphorylation is conveyed via mTORC1, but not mTORC2, HEK293E cells were depleted of mTORC1-specific component raptor or mTORC2-specific factor rictor, respectively. While raptor depletion abolished eIF2β phosphorylation, decrease in rictor expression did not exert a significant effect on phospho-eIF2β levels, but as expected decreased phosphorylation of the mTORC2 target AKT as compared with control (Fig. 3a,b). Either raptor or rictor depletion induced phospho-eIF2α (Fig. 3a,b), thus indicating that mTORC1 and mTORC2 influence eIF2α phosphorylation by distinct mechanisms likely as a response to different stimuli (see discussion).

### Phospho-eIF2β suppresses translation of ATF4 mRNA

We next determined whether eIF2β phosphorylation plays a role in translation regulation. Although initial reports suggested that eIF2β may be dispensable for translation^25^,^26^, subsequent studies demonstrated that eIF2β facilitates tRNA binding to eIF2 (ref. 27). Overexpression of non-phosphorylatable S(2,67)A and TOS eIF2β mutants increased eIF2α phosphorylation relative to WT eIF2β even in serum-fed cells and this effect was comparable with endoplasmic reticulum stress inducer thapsigargin (Fig. 3c; compare lanes 2, 4 and 5 with lanes 1 and 3). Induction of ATF4 protein levels by serum starvation was mitigated in S(2,67)D eIF2β mutant-expressing cells, as compared with control cells and cells expressing WT, S(2,67)A or TOS eIF2β mutants (Fig. 3d; compare lane 5 with lanes 1–4). Similarly to thapsigargin, S(2,67)A or TOS eIF2β mutants induced expression of a luciferase reporter mRNA bearing the 5′UTR of human ATF4 (p5′UTR ATF4-firefly luciferase)^28^ (Fig. 3e). Moreover, addition of recombinant eIF2β WT, but not S(2,67)A or TOS mutants, increased mRNA translation in rabbit reticulocyte lysate (RRL) until amounts of eIF2β exceeding levels of eIF2α and eIF2γ were reached (Supplementary Fig. 3a). To confirm these results, RRL was depleted of eIF2 and reconstituted by adding equimolar amounts of eIF2α/eIF2γ and increasing amounts of recombinant eIF2β WT, S(2,67)A or TOS mutants (Supplementary Fig. 3b). Although all three eIF2β variants were capable of supporting basal levels of mRNA translation as illustrated by higher translational activity in all reconstituted RRLs, as compared with eIF2-depleted RRL, only WT eIF2β increased mRNA translation in a concentration-dependent manner (Supplementary Fig. 3c). Importantly, recombinant WT, but not S(2,67)A or TOS eIF2β mutants, were phosphorylated on Ser2 in RRL (Supplementary Fig. 3d), which is consistent with previous reports showing CK2 activity in RRL^29^. Cells expressing S(2,67)A or TOS eIF2β mutants exhibited lower global protein synthesis relative to WT or phosphomimetic S(2,67)D eIF2β mutant, as monitored by ^35^S-labelling (Fig. 3f). Monitoring absorbance profiles (254 nm) of cytosolic HEK293E extracts separated by ultracentrifugation on a 15–35% sucrose gradient further confirmed that global translation is reduced under conditions where eIF2β cannot be phosphorylated, as evidenced by an increase in 80S monosome peak in S(2,67)A and TOS eIF2β mutant- versus WT-expressing cells (Supplementary Fig. 3e). These data show that eIF2β phosphorylation correlates with decreased translation of mRNAs harbouring uORFs (for example, ATF4) while bolstering global protein synthesis.

### Phospho-eIF2β bolsters TC recycling and protein synthesis

**Figure 4:**
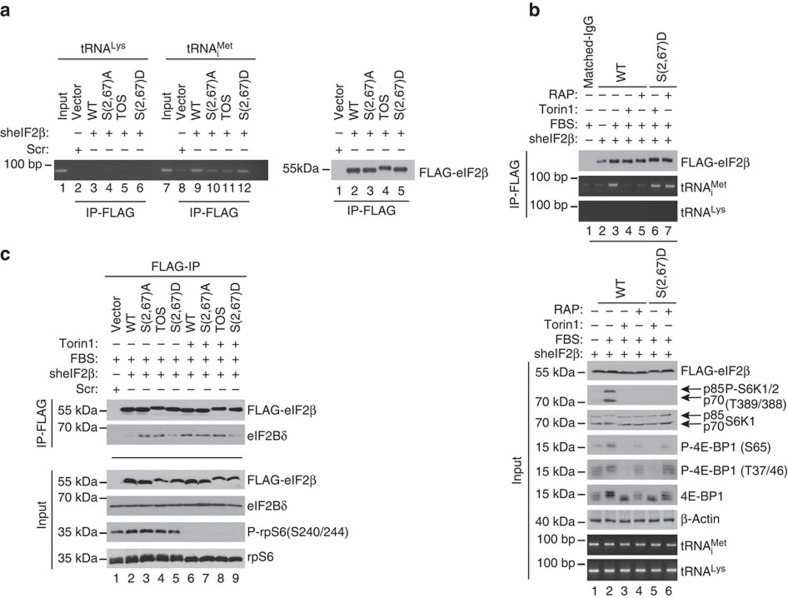
eIF2β phosphorylation stimulates TC recycling. (**a**) HEK293E cells that stably express exogenous WT or indicated FLAG-eIF2β mutants in which endogenous eIF2β was depleted by shRNA (Supplementary Fig. 4b; Methods) were serum starved for 16 h, stimulated with serum (10%) for 30 min and subjected to FLAG immunoprecipitation (IP). Inputs (10%; Supplementary Fig. 4c) and quantity of immunoprecipitated proteins (25%) was determined by western blotting. The amount of tRNA_i_^Met^ in the immunoprecipitated material was monitored by semi-quantitative reverse transcriptase–PCR (sqRT–PCR). tRNA^Lys^ was used as a negative control. (**b**) Cells described in **a** that express WT or S(2,67)D FLAG-eIF2β were serum starved for 16 h and then stimulated with 10% serum (fetal bovine serum, FBS) in the presence of a vehicle (DMSO), rapamycin (50 nM) or torin1 (250 nM) for 30 min. Lysates were immunoprecipitated with an anti-FLAG antibody. The quantity of tRNA_i_^Met^ and tRNA^Lys^ in immunoprecipitates and input (10%) was analysed by sqRT–PCR, whereas the levels of indicated protein were determined by western blotting. (**c**) Cells described in (**a**) were serum starved for 16 h and then stimulated with 10% serum (FBS) for 30 min in the presence of a vehicle (DMSO) or torin1 (250 nM). Immunoprecipitations were carried out as described in **a**. The amount of indicated proteins in FLAG-eIF2β immunoprecipitated material and inputs (10%) was monitored by western blotting. (**b**,**c**) Western blotting experiments were performed in independent duplicates and the representative results are shown. sqRT–PCR (*n*=3) results were independently confirmed using quantitative RT–PCR (Supplementary Fig. 4d,e).

We next investigated whether eIF2β mediates effects of mTORC1 on eIF2α phosphorylation and mRNA translation. To avoid interference of the endogenous eIF2β, and since depletion of eIF2β reduced cell viability^30^ (Supplementary Fig. 4a), we first overexpressed FLAG-tagged WT, S(2,67)A, TOS and S(2,67)D eIF2β mutants in HEK293E cells and then depleted endogenous eIF2β by short hairpin RNA (shRNA; Supplementary Fig. 4b). In serum-stimulated cells, eIF2β phosphorylation was detected in vector/scrambled shRNA-infected control cells, which still express endogenous eIF2β and cells expressing exogenous WT eIF2β, but not in cells expressing exogenous mutant forms of eIF2β with mutated phospho-acceptor sites to generate non-phosphorylatable (S(2,67)A) or phosphomimetic (S(2,67)D) mutants or disrupted TOS motif (TOS; Fig. 3g; compare lanes 1–2 versus 3–5; Supplementary Fig. 4c). Phospho-eIF2α and ATF4 protein levels were higher in non-phosphorylatable S(2,67)A and TOS eIF2β mutants, as compared with control, WT and phosphomimetic S(2,67)D eIF2β mutant-expressing cells (Fig. 3g; compare lanes 3–4 versus 1–2 and 5). Although mTOR activity was comparably reduced by torin1 across all cell lines as monitored by inhibition of S6K phosphorylation (Fig. 3g; compare lanes 2–5 with lanes 6–10), only S(2,67)D eIF2β mutant attenuated induction of eIF2α phosphorylation and dampened increase in ATF4 expression (Fig. 3g; compare lanes 6–9 with 10). Neither serum-stimulated nor torin1-treated cells (4 h) exhibited major differences in steady-state ATF4 mRNA levels (Fig. 3h), thereby suggesting that the observed effects on ATF4 protein levels after 4 h treatments most likely occur at the level of translation. These results demonstrate that eIF2β phosphorylation plays a major role in mediating mTOR-dependent downregulation of ATF4 protein synthesis and stimulation of global mRNA translation. Since we observed that phosphorylation status of eIF2β appears to affect eIF2α phosphorylation, we set out to determine the role of eIF2β phosphorylation in TC recycling using HEK293E cells, which express eIF2β variants and are depleted of endogenous eIF2β (Supplementary Fig. 4b). After 30-min serum stimulation, S(2,67)A and TOS eIF2β mutants immunoprecipitated drastically lower amounts of tRNA_i_^Met^ as compared with WT and S(2,67)D eIF2β mutant (Fig. 4a; compare lanes 10–11 with lanes 9 and 12; Supplementary Fig. 4d). In turn, rapamycin and torin1 prevented serum-induced increase in tRNA_i_^Met^ binding to WT eIF2β, while not exerting a major effect on the amount of tRNA_i_^Met^ in immunoprecipitates from S(2,67)D eIF2β-expressing cells (Fig. 4b; compare lanes 4–5 and 6–7 in the top panel, Supplementary Fig. 4e). eIF2α phosphorylation inhibits GEF activity of eIF2B and eIF2:eIF2B dissociation^2^. We therefore investigated whether eIF2β phosphorylation affects eIF2:eIF2B association by monitoring the amount of eIF2Bδ, which mediates recruitment of eIF2B to eIF2 (ref. 31) by immunoprecipitating cell lysates with an anti-FLAG antibody. In serum-stimulated cells, the amount of eIF2Bδ was higher in immunoprecipitated material from S(2,67)A and TOS eIF2β mutant-expressing cells, as compared with those expressing WT and S(2,67)D eIF2β mutant (Fig. 4c). In turn, torin1 increased WT eIF2β:eIF2Bδ, but not S(2,67)D eIF2β:eIF2Bδ association, to the levels observed in S(2,67)A and TOS eIF2β mutant-expressing cells (Fig. 4c; compare lane 2 with lane 6 versus lane 5 with lane 9). Expression of exogenous eIF2β variants did not conspicuously affect mTORC1 signalling (for example, Fig. 3g), thereby indicating that these effects were mediated by eIF2β and not other mTORC1 substrates. Altogether, these findings show that eIF2β phosphorylation decreases eIF2α phosphorylation and increases eIF2:tRNA_i_^Met^ binding while stimulating dissociation of eIF2B from eIF2. This suggests that phospho-eIF2β stimulates TC recycling. Phosphorylation status of eIF2α is determined via the action of eIF2α kinases (for example, PKR and PERK) and phosphatases (for example, protein phosphatase 1 (PP1))^4^. Notwithstanding that our results show that phosphorylation status of eIF2β affects phospho-eIF2α levels (Fig. 3c,d,g; Supplementary Figs 4c and 5a); PERK and PKR activation status as monitored by Thr981 and Thr446 appeared to be largely unaffected by eIF2β WT or mutant proteins (Supplementary Fig. 5a). Moreover, although concentration-dependent induction of PERK phosphorylation by thapsigargin was comparable between WT and S(2,67)D eIF2β mutant, corresponding induction of ATF4 protein expression was seemingly reduced in S(2,67)D eIF2β mutant, relative to WT eIF2β-expressing cells (Supplementary Fig. 5b). These observations suggest that the effects of eIF2β on eIF2α phosphorylation are not likely to be mediated by PERK or PKR.

### Phospho-eIF2β recruits NCK1 to eIF2

**Figure 5:**
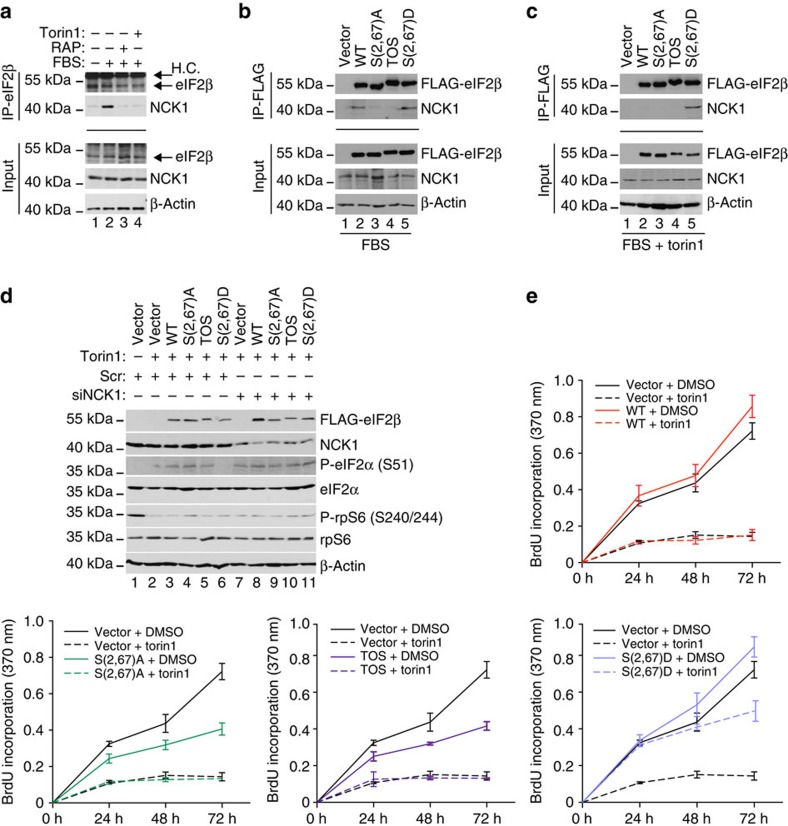
Phospho-eIF2β recruits NCK1 to eIF2 and mediates the effects of mTORC1 on proliferation. (**a**) HEK293E cells were depleted of serum for 16 h and then stimulated with 10% serum (fetal bovine serum, FBS) in the presence of a vehicle (DMSO), rapamycin (50 nM) or torin1 (250 nM) for 30 min. Anti-eIF2β antibody immunoprecipitates (25%) and corresponding inputs (10%) were analysed by western blotting using indicated antibodies. H.C., heavy chains (**b**,**c**) HEK293E cells were transfected with the indicated eIF2β constructs, serum starved for 16 h and stimulated with 10% serum (FBS) in combination with a vehicle (DMSO) (**b**) or torin1 (250 nM) (**c**). Anti-FLAG antibody immunoprecipitates (25%) and corresponding inputs (10%) were analysed by western blotting using indicated antibodies. (**d**) HEK293E cells expressing indicated eIF2β constructs were transfected with a control, scrambled siRNA (Scr) or siRNA targeting NCK1 (siNCK1), serum starved and then stimulated with 10% serum in the presence of a vehicle (DMSO) or torin1 (250 nM) for 30 min. Expression and phosphorylation status of indicated proteins were monitored by western blotting. β-Actin served as a loading control. Experiments in **a**–**d** were carried out two times independently. (**e**) HEK293E cells expressing indicated eIF2β variants in which endogenous eIF2β was depleted by shRNA (Supplementary Fig. 4b) were treated with a vehicle (DMSO) or torin1 (250 nM) for indicated times. Proliferation was measured by 5-bromo-2′-deoxyuridine (BrdU) incorporation, and the results are represented as mean absorbance at 370 nm±s.d. from three independent experiments, each consisting of a technical duplicate.

eIF2β binds the non-catalytic region of tyrosine kinase adaptor protein 1 (NCK1), which has been implicated in recruitment of PP1 to eIF2 leading to eIF2α dephosphorylation^32^. Consistently, we observed that the levels of NCK1 are reduced in eIF2-depleted RRL (Supplementary Fig. 3b, left panel), and although it has been suggested that eIF2β phosphorylation may negatively regulate its binding to NCK1 using *in vitro* binding assay^33^, insulin, which we show strongly increases phosphorylation of eIF2β, stimulates recruitment of NCK1 to ribosomes^34^. To address the potential role of NCK1 in mediating the effects of phospho-eIF2β on eIF2α phosphorylation, immunoprecipitations were carried out in parental HEK293E cells or in HEK293E cells expressing WT or eIF2β mutants. Endogenous eIF2β and NCK1 were co-immunoprecipitated in serum-stimulated cells, but not in serum-starved cells (Fig. 5a; compare lanes 1 and 2), wherein eIF2β:NCK1 interaction was disrupted by mTOR inhibitors (Fig. 5a; lanes 3–4). In serum-stimulated cells, the amount of NCK1 in the material immunoprecipitated with eIF2β WT and S(2,67)D mutants was higher relative to the amounts of NCK1 co-immunoprecipitated with S(2,67)A and TOS eIF2β mutants (Fig. 5b; lanes 2 and 5 versus lanes 3 and 4). Torin1 decreased NCK1:WT eIF2β but not NCK1:S(2,67)D eIF2β co-immunoprecipitation (Fig. 5c; lane 2 versus 5). We showed that the loss of PTEN attenuates the effects of CK2 inhibition on eIF2β phosphorylation (Fig. 2e). Consistently, CX-4945 reduced WT eIF2β phosphorylation and NCK1:WT eIF2β association after 1 h in HCT116 PTEN^+/+^ cells, whereas 1-h exposure to CX-4945 was insufficient to decrease NCK1:WT eIF2β association and WT eIF2β phosphorylation in HCT116 PTEN^−/−^ cells (Supplementary Fig. 5c,d). In turn, 7-h CX-4945 treatment reduced NCK1:WT eIF2β association that was paralleled by decreased exogenous WT eIF2β phosphorylation (Supplementary Fig. 5d). These findings show that, at least in part, the effects of phospho-eIF2β on phospho-eIF2α status are mediated via recruitment of NCK1 to eIF2. We observed that S(2,67)D eIF2β mutant antagonizes torin1-induced upregulation in phopsho-eIF2α levels compared with all other eIF2β variants (Fig. 3g, lane 10 versus lanes 6–9). Therefore, to further confirm that NCK1 mediates the effects of phopsho-eIF2β on eIF2α phosphorylation, we investigated whether downregulation of NCK1 will abolish the ability of S(2,67)D eIF2β mutant to antagonize increase in eIF2α phosphorylation induced by torin1. Indeed, although expression of S(2,67)D eIF2β was sufficient to antagonize induction of eIF2α phosphorylation by torin1 in control, scrambled short interfering RNA (siRNA)-transfected cells, this effect was lost in cells in which NCK1 levels were reduced by siRNA (Fig. 5d; lane 6 versus 11). Collectively, these data suggest a model, whereby eIF2β phosphorylation stimulates NCK1 recruitment to eIF2, which leads to decrease in phopsho-eIF2α levels.

### eIF2β mediates effects of mTOR and CK2 on proliferation

Our results indicate that eIF2β phosphorylation mediates the effects of mTORC1 and CK2 on TC formation and global protein synthesis. Since global protein synthesis rates closely correlate with proliferation rates^35^, we investigated whether eIF2β acts as a mediator of the effects of mTORC1 and CK2 on proliferation of cells expressing WT, S(2,67)A, S(2,67)D or TOS eIF2β mutants, wherein endogenous eIF2β was depleted (Supplementary Fig. 4b). In 10% serum, cells expressing S(2,67)A and TOS eIF2β mutants proliferated slower than those expressing WT eIF2β, whereas S(2,67)D eIF2β mutant-expressing cell exhibited slightly increased proliferation relative to WT eIF2β (Fig. 5e). Anti-proliferative effects of torin1, serum depletion or amino-acid deprivation were markedly attenuated by S(2,67)D eIF2β mutant as compared with WT eIF2β-expressing cells (Fig. 5e; Supplementary Fig. 6a–c). In addition, CX-4945 strongly reduced proliferation of WT eIF2β-expressing cells while having much lesser effect on the proliferation of cells expressing the S(2,67)D eIF2β mutant (Supplementary Fig. 7a). These findings demonstrate that eIF2β phosphorylation plays a major role in mediating the effects of mTORC1 and CK2 on cell proliferation.

NCK1 is thought to stimulate dephosphorylation of eIF2α via recruitment of PP1 (ref. 32). Thus, we treated cells with salubrinal, which inhibits eIF2α dephosphorylation by PP1 (ref. 36). In contrast to inhibition of mTORC1 or CK2 signalling, S(2,67)D eIF2β mutant failed to attenuate anti-proliferative effects of salubrinal (Supplementary Fig. 7b). This was paralleled by the inability of S(2,67)D eIF2β mutant to impede salubrinal-induced eIF2α phosphorylation (Supplementary Fig. 7c). Moreover, induction of expression of non-phosporylatable S51A eIF2α mutant in HT1080 cells^37^ abolished the stimulatory effects of S(2,67)D eIF2β on proliferation (Supplementary Fig. 8a–c). Taken together, these findings show that the effects of eIF2β on cellular proliferation are mediated by eIF2α phosphorylation and put forward a model whereby CK2 and mTORC1 bolster TC formation via eIF2β phosphorylation, followed by the NCK1-dependent dephosphorylation of eIF2α. However, additional roles of NCK1 that lead to the inhibition of eIF2α phosphorylation, such as NCK1-dependent inhibition of PERK^38^ may also play a role in this process. Moreover, eIF2β associates with tRNA_i_^Met^ (ref. 39), as well as the additional translation initiation factors including eIF1 (ref. 40), eIF1A^39^, eIF2B^41^ and eIF5 (ref. 42). Therefore, it is plausible that at least some of the effects of eIF2β phosphorylation on TC recycling, protein synthesis and proliferation are mediated via eIF2α-independent mechanisms including direct modulation of eIF2β:tRNA_i_^Met^ interaction or alteration of eIF2β interaction with translation initiation factors other than eIF2 subunits.

## Discussion

**Figure 6:**
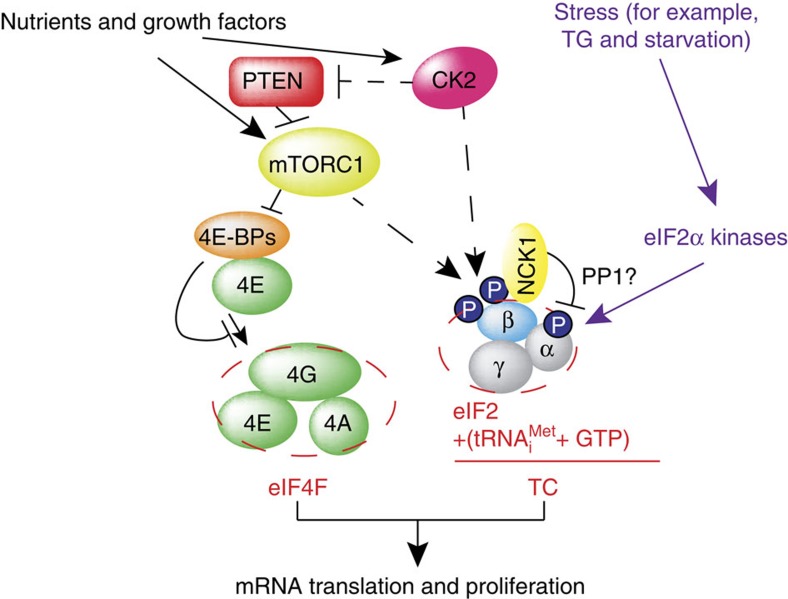
Schematic representation of the proposed model for the coordination of ternary complex (TC) and eIF4F assembly by mTOR and CK2. On acute stimulation with nutrients, growth factors and insulin, CK2 appears to bolster mTORC1 activity, which is likely mediated via inhibition of PTEN. This leads to phosphorylation and inactivation of 4E-BPs, thereby facilitating eIF4F complex assembly. Simultaneously, CK2 bolsters TC formation by phosphorylating eIF2β. Phosphorylation of eIF2β results in the recruitment of NCK1 to eIF2, which correlates with eIF2α dephosphorylation, likely mediated by PP1. mTORC1 also stimulates eIF2β phosphorylation independently of CK2, which along with its established role in inducing eIF4F levels, demonstrates that mTORC1 also may coordinate TC and eIF4F assembly. In response to chronic increase in protein synthesis or stress (for example, thapsigargin (TG); shown in purple), activation of eIF2α kinases overcomes the effects of eIF2β phosphorylation on TC recycling, thereby allowing cells to fine-tune protein synthesis levels and energy consumption and/or adapt to stress. Broken lines represent uncertainties, such as precise hierarchy of the effects of CK2 and mTORC1 on eIF2β (Ser2) phosphorylation.

It has been shown that in yeast, inhibition of TOR signalling leads to eIF2α phosphorylation via GCN2 kinase^43^,^44^,^45^. In mammals, the PI3K/AKT/mTOR and eIF2α signalling have been linked by multiple mechanisms that appear to be dependent on a stressor, length of exposure to stress or proliferation status of the cell^4^. These mechanisms include inhibition of PERK and GCN2 via the mTORC2/AKT pathway and PKR via PTEN^46^,^47^,^48^. eIF2α phosphorylation and mTORC1 have also been linked via the catalytic subunit of protein phosphatase 6 (PP6C)^49^. Our study unravels a hitherto unprecedented mechanism of translational regulation, whereby acute activation of mTORC1 bolsters TC formation by inducing phosphorylation of eIF2β, which in turn stimulates the recruitment of NCK1 to eIF2, thereby leading to eIF2α dephosphorylation (Fig. 6). In turn, constitutive activation of mTORC1 leads to a decrease in AKT activity via activation of S6K1-dependent negative feedback mechanism and induction of endoplasmic reticulum stress due to chronically elevated protein synthesis that results in induction of eIF2α kinases and eIF2α phosphorylation^50^,^51^. Collectively, these findings suggest a fine-tuning mechanism, whereby stimulation of mTORC1 initially leads to upregulation in TC levels to allow induction of translation, whereas prolonged mTOR-dependent translational activation is compensated by downregulation of TC recycling, thereby preserving protein and energy homeostasis. Moreover, although induction of ATF4 expression by thapsigargin was attenuated by phosphomimetic eIF2β mutant as compared with those expressing WT eIF2β, higher thapsigargin concentrations were sufficient to increased ATF4 levels even in phosphomimetic S(2,67)D eIF2β mutant-expressing cells (Supplementary Fig. 5b). This suggests that induction of stress above a certain threshold is likely to override the inhibitory effects of eIF2β phosphorylation on ATF4 expression, thereby antagonizing stimulatory effects of the mTORC1/eIF2β axis on translation and proliferation and allowing cells to rapidly adapt to stress.

We show that acute inhibition of mTORC1 in addition to reducing translation of TOP and ‘eIF4E-sensitive' mRNAs via LARP1- and 4E-BP-dependent mechanisms, respectively^52^,^53^,^54^,^55^,^56^, also upregulates translation of ‘eIF2α-sensitive' mRNAs (for example, ATF4) that appears to be mediated via reduction of eIF2β phosphorylation. In contrast to endoplasmic reticulum stressors such as thapsigargin^57^, acute inhibition of the mTORC1/4E-BP/eIF4E axis has a lesser effect on global protein synthesis and is therefore expected to only marginally effect on translation of housekeeping mRNAs (for example, β-actin)^58^. Therefore, although mTOR inhibitors and inducers of eIF2α kinases increase eIF2α phosphorylation, their impact on the translatome appears to be different. Notwithstanding that mTOR and eIF2α kinase employ a range of distinct effectors, these findings further corroborate a tenet that translation regulation under acute stress or stress recovery is achieved via the cross-talk between the mTOR and eIF2α kinase pathways. Moreover, the complexity of cross-talk between mTOR and eIF2α kinases is further illustrated by studies showing that although we observe translational activation of ATF4 mRNA after 4 h, it is known that prolonged treatment with mTOR inhibitors (>12 h) suppresses ATF4 expression at the level of transcription^59^. Future studies are however required to decipher molecular underpinnings of mTOR/eIF2α kinase cross-talk and its role in stress response.

CK2 and mTORC1 appear to phosphorylate overlapping sites on human eIF2β, namely, Ser2 and Ser67. Strikingly, the effects of mTORC1 on phosphorylation of eIF2β on Ser2 are affected by the changes in CK2 activity and vice versa CK2 stimulation of Ser2 eIF2β phosphorylation is attenuated in cells wherein CK2 signalling is uncoupled from mTOR. These observations suggest a complex relationship between CK2 and mTOR in regulating eIF2β phosphorylation. Intriguingly, inspection of publicly available mass spectrometry data indicates the existence of additional phospho-sites on eIF2β that appear to be targeted by mTORC1 and likely CK2 (Ser105 and Thr111)^60^,^61^,^62^. These additional phospho-acceptor sites on eIF2β may therefore act as priming sites for Ser2 phosphorylation. This may explain delayed effects of mTOR and CK2 inhibitors on Ser2 eIF2β phosphorylation in cells in which CK2 is overexpressed, or wherein CK2 is uncoupled from mTORC1, respectively. However, future studies are required to determine the precise hierarchy of mTORC1- and CK2-dependent eIF2β phosphorylation.

Our results show that eIF2β phosphorylation-dependent stimulation of TC formation is synchronized with eIF4F complex assembly, which is mediated by CK2 and mTORC1. Inactivation of 4E-BPs by mTORC1-dependent phosphorylation stimulates eIF4F complex assembly^1^, whereas mTORC1-mediated phosphorylation of eIF2β appears to simultaneously facilitate TC recycling. CK2 also appears to bolster TC assembly via direct phosphorylation of eIF2β, which is consistent with previous findings showing that decreased CK2 activity coincides with increased eIF2α phosphorylation and elevated CHOP expression^63^. At the same time, CK2 bolsters 4E-BP phosphorylation by activating mTORC1, which at least in part is mediated via inhibition of PTEN. Since dysregulated mTORC1 and CK2 signalling underpin a number of diseases characterized by aberrant protein synthesis and proliferation including cancer, our findings suggest that eIF2β phosphorylation may also play a prominent role in these pathologies.

## Methods

### Cell culture and compounds

HEK293E and MCF7 cells were obtained from American Type Culture Collection (ATCC) and maintained in DMEM and RPMI-1640, respectively, supplemented with 10% fetal bovine serum, 1% penicillin/streptomycin and 1% L-glutamine (all from Wisent Bio Products). HCT116 PTEN^+/+^ and PTEN^−/−^ cells were obtained from Dr Waldman^19^ and maintained in DMEM supplemented with 10% fetal bovine serum, 1% penicillin/streptomycin and 1% L-glutamine. U2OS cells that overexpress CK2α/β under the control of TetOFF system^16^ were maintained in DMEM supplemented with 10% fetal bovine serum, 1% penicillin/streptomycin, 1% L-glutamine and 1.5 μg ml^−1^ tetracycline (Sigma-Aldrich) to suppress CK2α/β expression. CK2α/β expression was induced by washing the cells three times in 1 × PBS, and incubating them in tetracycline-free media for 16 h before treating cells as indicated in figure legends. HT1080 cells expressing eIF2α WT and HA-eIF2α KI^37^ were maintained in DMEM supplemented with 10% fetal bovine serum, 1% penicillin/streptomycin and 1% L-glutamine in the presence of 2 μg ml^−1^ puromycin (Sigma-Aldrich). TSC WT and knockout MEFs were maintained in DMEM supplemented with 10% fetal bovine serum, 1% penicillin/streptomycin and 1% L-glutamine. All culture media and reagents were obtained from Wisent Inc, unless specified. All cell lines were maintained in 5% CO_2_ at 37 °C. Micoplasma contamination was excluded using Mycoplasma PCR Detection Kit (Applied Biological Materials Inc). Where indicated, cells were treated with 250 nM torin1 (Tocris), 50 nM rapamycin (Calbiochem), 3 μM Ku-0063794 (Tocris), 10–50 μM CX-4945 (Adooq Bioscience), 1 μM thapsigargin (Sigma-Aldrich) or 1 μM salubrinal (Tocris). All drugs were stored in aliquots at −80 °C in DMSO for no longer than 6 months, and equal volume of DMSO was used as a vehicle control. HEK293E cells were freshly obtained from ATCC before the experiments (293 c18 (ATCC CRL-10852)), whereas the rest of the cell lines used were verified by profiling 17 short tandem repeat (STR) and 1 gender determining loci using ATCC Cell Line Authentication Service and the results were as following:

HCT116 (94% match to ATCC HCT116 # CCL-247): D5S818 (10, 11 versus 10, 11); D13S317 (10, 12 versus 10, 12); D7S820 (11, 12 versus 11, 12); D16S539 (11, 12, 13, 14 versus 11, 13); vWA (17, 21, 22, 23 versus 17, 22); THO1 (8, 9 versus 8,9); AMEL (X versus XY); TPOX (8, 9 versus 8, 9), CSF1PO (7, 10 versus 7, 10).

MCF7 (100% match to ATCC MCF7 cells # HTB-22): D5S818 (11, 12 versus 11, 12); D13S317 (11 versus 11); D7S820 (8, 9 versus 8, 9); D16S539 (11, 12 versus 11, 12); vWA (14, 15 versus 14, 15); THO1 (6 versus 6); AMEL (X versus X); TPOX (9, 12 versus 9, 12), CSF1PO (10 versus 10).

U2OS (93% match to ATCC U2OS cells # HTB-96): D5S818 (8, 11 versus 11); D13S317 (13 versus 13); D7S820 (11, 12 versus 11, 12); D16S539 (11 versus 11, 12); vWA (14, 18 versus 14, 18); THO1 (6, 9.3 versus 6, 9.3); AMEL (X versus X); TPOX (11, 12 versus 11, 12), CSF1PO (12, 13 versus 13).

HT1080 (93% match to ATCC HT1080 cells # CCL-121): D5S818 (11, 13 versus 11, 13); D13S317 (14 versus 12, 14); D7S820 (9, 10 versus 9, 10); D16S539 (9, 12 versus 9, 12); vWA (14, 19 versus 14, 19); THO1 (6 versus 6); AMEL (X, Y versus X, Y); TPOX (8 versus 8), CSF1PO (12 versus 12).

TSC2 WT and knockout MEFs were generated by Kwiatkowski's group and obtained from Sonenberg's lab.

### Constructs and recombinant proteins

Human eIF2β was subcloned from pOTB7 vector (provided by Dr Kimchi) in pEF-FLAG vector (generously provided by Dr Ronai) using SalI and BamHI enzymes. Ser2 and Ser67 were mutated into an alanine (A) or aspartate (D) and phenylalanine 89 into an alanine (TOS mutant) using Quick-Change Site-Directed Mutagenesis (Agilent Technologies). For stable expression in mammalian cells, FLAG-eIF2β variants were subcloned in pWPI-GFP vector (Addgene) using PmeI enzyme. To produce the recombinant proteins, eIF2β was subcloned in pGEX-6p-1 vector (GE Healthcare Life Sciences) using BamHI and EcoRI. Recombinant proteins were purified as follows: 50 ml of BL21 (DE3) competent *E. coli* transformed by heat shock with ∼300 ng of pGEX-6p-1-eIF2β constructs (encoding WT or S(2,67)A, TOS and S(2,67)D eIF2β mutants) was induced with isopropylthiogalactoside for 3 h and then lysed in lysis buffer (50 mM Tris HCl (pH 7.5), 300 mM NaCl, 10 μM TCEP (tris(2-carboxyethyl)phosphine; Sigma-Aldrich)). Lysates were incubated with 20 μl of Glutathione Sepharose 4B (GE Healthcare Life Sciences) for 1 h at 4 °C and then washed twice with lysis buffer supplemented with 5% glycerol and 0.1% NP-40, and 700 mM NaCl. Recombinant proteins were then washed once in buffer B (20 mM Tris HCl pH 7.5, 100 mM KCl, 0.1 mM EDTA, 5% glycerol). For *in vitro* translation and circular dichroism experiments, glutathione *S*-transferase (GST) tag was removed with PreScission protease (GE Healthcare Life Sciences). GST tag was not removed for the *in vitro* kinase assay.

### siRNA, lentiviral shRNA and generation of cell lines expressing eIF2β variants depleted of endogenous eIF2β

Scrambled control (NS1) and siRNA targeting human NCK1 (5′-AACAUCCAUUACAUCUCCUUUCUCGAA-3′) were obtained from Integrated DNA Technologies. siRNA were transfected using Lipofectamine 2000 (Invitrogen) according to the manufacturer's instructions at the final concentration of 10 nM. Cells were lysed 72 h post transfection in RIPA buffer and analysed by western blotting. Lentiviral vectors carrying shRNA targeting human raptor (plasmid 1857), human rictor (plasmid 1854) and the non-target shRNA control (plasmid 1864) were from Addgene. shRNAs targeting human eIF2β (TRCN0000291996) and scrambled control (SHC002) were from Sigma-Aldrich (Mission collection). Vectors encoding shRNAs (7 μg) were co-transfected into 5 × 10^6^ HEK293T cells with 7 μg of each lentivirus packaging plasmids PLP1, PLP2 and PLP-VSVG (Invitrogen). Viral supernatant was collected 48 h post transfection, filtered through a Mixed Cellulose Ester filter (0.45 μm, Fisher Scientific), mixed in 1:1 ratio with growth media and added to cells for 24 h. Infection was carried out in the presence of 8 μg ml^−1^ of polybrene (Sigma-Aldrich). Forty-eight hours post infection, cells were selected and maintained in full growth media supplemented with 5 μg ml^−1^ puromycin. To generate cells lines expressing FLAG-eIF2β variants, viruses were generated and HEK293E cells were infected as described above. Expression of shRNA-insensitive eIF2β variants was monitored 72 h post infection by western blotting and cells were then infected with viruses carrying shRNA targeting eIF2β or scrambled control. Forty-eight hours post infection, cells were selected with 2 μg ml^−1^ puromycin as described above and expression of indicated exogenous eIF2β variants as well as depletion of endogenous eIF2β was determined by western blotting (Supplementary Fig. 4b).

### Antibodies and western blotting

For western blotting, cells were scraped in 1 × PBS (pH 7.4), centrifuged and lysed in RIPA buffer (10 mM Tris (pH 7.3), 1% (w/v) Na-deoxycholate, 1% (v/v) Triton, 150 mM NaCl, 1 mM EDTA, 50 mM NaF, 10 mM β-glycerophosphate and protease inhibitors) supplemented with complete protease inhibitors (Roche). Whole-cell protein extracts (10–60 μg) were analysed by SDS–polyacrylamide gel electrophoresis (SDS–PAGE; 6–15% polyacrylamide gels were used depending on molecular weight of analysed proteins) and transferred to nitrocellulose membrane (Bio-Rad) using wet transfer apparatus (Cleaver). Following antibodies were diluted as indicated in 5% (w/v) bovine serum albumin (Sigma-Aldrich) in 1 × TBS-Tween 20 and incubated overnight at 4 °C: anti-eIF2α (L57A5) #2103 (1:1,000), anti-p-eIF2α (S51) #9721 (1:1,000), anti-4E-BP1(53H11) #9644 (1:2000), anti-p-4E-BP1/2 (S37/46)(236B4) #2855 (1:1,000), anti-p-4E-BP1(S65)(174A9) #9456 (1:1,000), anti-p70 S6 kinase #9202 (1:1,000), anti-p-p70 S6K1/2 (T389/388) #9205 (1:1,000), anti-p-rpS6 (S240/244) #2215 (1:1,000), anti-p-AKT (S473) #9271 (1:1,000), anti-AKT (pan) (C67E7) #4691 (1:1,000), anti-NCK1 (15B9) #2319 (1:1,000), anti-raptor (24C12) #2280 dilution (1:1,000), anti-rictor #2140 (1:1,000); anti-PTEN (138G6) #9559 (1:1,000), anti-mTOR (7C10) #2983 (1:1,000), anti-PRAS40 #2610 (1:1,000), anti-p-PTEN (S380;T382/383) (44A7) #9549 (1:1,000), anti-PERK # 5683 (1:1,000), anti-p-PKR (T446) # 3076 (1:1,000), anti-eIF4G1 #2858 (1:1,000) and anti-Myc-Tag #2276 (1:1,000) all from Cell Signaling Technologies; anti-TSC2 sc-893 (1:500), anti-eIF2β (P-3) #sc-9978 (1:1,000), anti-ATF4 (CREB-2) C-20 #sc-200 (1:1,000), rpS6 (C-8) #sc-74459 (1:2,000) and anti-p-PERK (T981) sc-32577 (1:500) all from Santa Cruz Biotechnologies; anti-eEF1δ #A301–685A (1:2,000) and eIF2Bδ #A302–982A (1:1,000) both from Bethyl Labs; anti-FLAG(M2) #F3165 (1:5,000) and anti-β-actin (AC15) #A1978 (1:5,000) both from Sigma; anti-eIF4E #610269 (1:1,000) from BD Biosciences; anti- #ab9110 (1:2,000) from Abcam; anti-PKR (F9)^64^ (1:1,000), anti-p-eIF2β (S2) (1:10000), anti-p-eEF1δ (1:10,000)^65^ and anti-CK2α (1:1000) were generated in Litchfield's lab. Secondary antibodies (Amersham) were used at 1:10,000, and signals were revealed by chemiluminescence (ECL, GE Healthcare). Where possible, membranes were stripped and reprobed with indicated antibodies. In the cases where this was not possible (for example, wherein the phospho and total antibodies or antibodies recognizing same proteins were used and significant signal remained on the membrane after striping), same lysates were ran simultaneously on duplicate gels, and probed with phospho and total antibodies. Each experiment was performed at least twice independently and the representative data are shown. X-ray films and/or ECL scans of whole membranes are shown in Supplementary Fig. 10. Although we trust that western blotting should be used for qualitative rather than quantitative measurements, as requested by reviewers, we performed densitometric analysis using ImageJ (W. S. Rasband, ImageJ; National Institutes of Health, Bethesda, MD). The resulting data were log2 transformed, normalized per replicate and to the mean of the control, and analysed using analysis of variance in R (r-project.org) (Supplementary Fig. 9).

### *In vitro* kinase assay

For *in vitro* kinase assay, HEK293E cells were transfected in a 10-cm Petri dish with 7 μg of HA-Raptor using Lipofectamine 2000 (Invitrogen) according to manufacturer's instruction. HA-immunoprecipitation was performed in CHAPS buffer (40 mM HEPES KOH (pH 7.4), 2 mM EDTA, 10 mM sodium pyrophosphate, 0.1 M NaCl, 0.3% CHAPS), washed twice with CHAPS buffer and once in CHAPS buffer supplemented with 0.4 M NaCl. Beads were then washed twice in kinase buffer (25 mM HEPES KOH (pH 7.5), 50 mM KCl, 10 mM MgCl_2_) and resuspended in 40 μl of kinase buffer. One-sixth of the immunoporecipitated material and 1 μg of recombinant GST-eIF2β variants were used per reaction. Reaction was carried out at 30 °C for 30 min in a final volume of 50 μl, in the presence of 100 μM ATP, 0.5 μl of 10 μCi ml^−1^ of ^32^P-γATP (Perkin Elmer) and 1 × of kinase buffer. Reactions were stopped with 4 × Laemmli Sample Buffer and samples were loaded on a 10% SDS–PAGE gel. SDS–PAGE gel was then stained with Coomassie brilliant blue R-250, dried for 2 h at 80 °C and the phosphorylation was measured by ^32^P incorporation using Storm 860 Molecular Imager. Each experiment was carried out at least in two independent replicates.

### Reporter translation assays

*In vitro* translation assay and *in vitro* transcription of the reporter renilla mRNA were performed by depleting eIF2 using anti-eIF2β antibody. eIF2β was first linked to 10 μl of protein G agarose beads in 1 × at 4 °C for 2 h. Beads and antibody were then washed twice in PBS and twice in buffer D (25 mM HEPES KOH (pH 7.3), 50 mM KCl, 75 mM KOAc, 2 mM MgCl_2_). After the last wash, buffer D was removed and the antibody-conjugated beads were incubated with nuclease-treated RRL for 2 h at 4 °C. Purified eIF2α and eIF2γ lacking the eIF2β subunit were kindly provided by Yuri Svitkin from Sonenberg's laboratory. For translation reaction, 50 ng of the reporter mRNA was added to 7 μl of RLL in a final volume of 10 μl. Titration experiments were performed adding increasing amount of eIF2 β recombinant proteins (40, 80, 160 and 270 ng). In eIF2-depleted RRL, eIF2 was reconstituted by adding 37.5 or 75 ng of purified eIF2α/γ subunits and 37.5 or 75 ng of recombinant eIF2β. Translation reaction was carried out at 30 °C for 30 min and stopped adding equal volume of 2 × PBS. Renilla expression was measured in 3 μl of RRL with 50 μl of Renilla Luciferase Assay system (Promega).

Translation of the reporter mRNAs harbouring 5′UTR of human ATF4 (ref. 28). was monitored by dual-luciferase assay. HEK293E cells expressing eIF2β variants were seeded in a 10-cm Petri dish and transfected with a mixture of 600 ng of pRenilla (provided by Dr Sonenberg) and 1.2 μg of p5′UTR ATF4-firefly luciferase reporter vectors^28^ using Lipofectamin 2000 (Invitrogen) according to manufacturer's instruction. Twenty-four hours post transfection, 9 × 10^5^ cells were seeded in a six-well plate in triplicate and the luciferase assay was performed at 48 h post transfection with cells at 80% confluency. Cells were collected in Passive Lysis Buffer, and firefly and renilla luciferase activity was measured using the Dual-luciferase Reporter Assay kit (Promega) according to the manufacturer's instructions. Experiments were repeated three times independently (*n*=3), whereby each biological replicate consisted of a technical duplicate.

### Genome-wide polysome profiling and RT–qPCR

Polysome profiling^6^ was performed in four independent biological replicates. MCF7 cells were seeded in a 15-cm Petri dish, serum starved for 16 h (‘control') and then treated with 4.2 nM of recombinant human insulin (Sigma-Aldrich) for 4 h alone (‘insulin') or in combination with 250 nM torin1 (‘insulin+torin1'). Cells were collected at 80% confluency and lysed in hypotonic lysis buffer (5 mM Tris HCl pH (7.5), 2.5 mM MgCl_2_, 1.5 mM KCl, 100 μg ml^−1^ cycloheximide, 2 mM dithiothreitol (DTT), 0.5% Triton, 0.5% sodium deoxycholate). Ten per cent of the lysates was saved to isolate cytoplasmic mRNA. The amount of RNA in each lysate was measured at 254 nm and 12 ODs were loaded on 5–50% sucrose gradients generated using Gradient Master (Biocomp) and subjected to ultracentrifugation (SW41 rotor; Beckman 36,000, 2 h and 4 °C). Sucrose gradients were fractionated by displacement by 60% sucrose/0.01% bromphenol blue, using ISCO Foxy fraction collector (35 s for each fraction=750 μl per fraction) equipped with a ultraviolet lamp for continuous absorbance monitoring. Fractions were flash-frozen immediately after fractionation and stored at −80 °C. RNA was isolated with Trizol (Thermo Fisher Scientific) according to the manufacturer's instruction. For microarray analysis, fractions corresponding to heavy polysomes (more than three ribsomes) were pooled (Fig. 1a). RNA was submitted to the Bioinformatics and Expression analysis core facility at Karolinska Institutet. RNA quality was assessed using an Agilent 2100 Bioanalyzer (Agilent Technologies) and complementary DNA was generated and hybridized onto the Affymetrix Human Gene 1.1 ST Array. The oligo package version 1.30.0 was used to summarize and normalize expression data using robust multiarray average (rma) in R version 3.1.1 (www.r-project.org) and bioconductor version 3.0. We used updated probe set definitions^66^ as these showed improved precision and accuracy^67^. We assessed the reproducibility using principal component analysis. Samples clustered according to RNA (cytosolic or polysome associated) and experimental group (control, insulin or insulin+torin1) indicating good reproducibility. We obtained mean expression levels for each treatment and RNA combination. These were then used to calculate mean log_2_ fold changes between insulin+torin1 versus insulin and insulin versus control for each RNA separately (polysome associated or cytosolic). Two-sided Wilcoxon rank-sum test was used to compare differences in fold changes between mRNAs whose translation was stimulated by phosphorylation of eIF2α^5^ to those that were not using data from polysome-associated mRNA or cytoplasmic mRNA separately. Data are displayed in Fig. 1. Raw and processed data are available at the Gene Expression Omnibus (GSE76766). For reverse transcriptase–quantitative PCRs (RT–qPCRs), RNA was extracted using Trizol according to the manufacturer's instructions. RT–qPCRs were performed using SuperScript III Reverse Transcriptase, followed by Fast SYBR Green Mastermix (both from Invitrogen), according to the manufacturer's instructions. Analyses were carried out using relative standard curve method as described in http://www3.appliedbiosystems.com/cms/groups/mcb_support/documents/generaldocuments/cms_040980.pdf. Experiments were performed at least in independent duplicates (*n*=2), whereby every sample was analysed in a technical triplicate. Primers were designed using NCBI Primer-BLAST (http://www.ncbi.nlm.nih.gov/tools/primer-blast/) such that *T*_m_ was between 57 and 63 °C, *T*_m_ difference was <3 °C and that primer pairs were separated by at least one intron. Primers were obtained from Integrated DNA Technologies, and their sequences and size of the amplicons are listed below:

ATF4 (NM_001675.4, NM_182810.2); Amplicon=226 nt

Human ATF4-Forward 5′-TCAAACCTCATGGGTTCTCC-3′

Human ATF4-Reverse 5′-GTGTCATCCAACGTGGTCAG-3′

Note: these primers recognize *Homo sapiens* ATF4, transcript variant 1, mRNA (NM_001675.4) and *Homo sapiens* ATF4, transcript variant 2, mRNA (NM_182810.2), both of which contain inhibitory uORFs and are translationally activated when eIF2α phosphorylation is induced and encode identical protein^68^,^69^. PCR product is of same size irrespective which variant is amplified.

β-Actin (ACTB; NM_001101.3); Amplicon=163 nt

Actin HF 5′- ACCACACCTTCTACAATGAGC-3′

Actin HR 5′- GATAGCACAGCCTGGATAGC-3′

### Metabolic (^35^S-methionine/cysteine) labelling

For ^35^S-methionine/cysteine labelling, HEK293E cells were seeded in six-well plates, serum starved for 16 h and then deprived of methionine and cysteine for an additional 2 h using DMEM without the above amino acids (Gibco). Cells were then treated with 10% dialysed serum containing 10 μCi ml^−1^ of ^35^S-Met/Cys (Perkin Elmer) and 250 nM of torin1 were indicated. Cells were lysed in RIPA buffer without SDS and 10 μl of the protein extract measured by LS6500 Multi Purpose Scintillation Counter (Beckman Coulter). Experiments were performed in independent triplicates (*n*=3) each of which was performed in a technical duplicate.

### Cap (m^7^GTP) pull-down assay

MCF7 and HCT116 cells were treated as described in Supplementary Fig. 1a, and lysed in two volumes of buffer B (50 mM MOPS KOH (pH 7.4), 100 mM NaCl, 50 mM NaF 2 mM EDTA, 2 mM EGTA, 1% NP-40, 1% sodium deoxycholate, 7 mM β-mercaptoethanol, protease inhibitors and phosphatase inhibitor cocktail 1 (Sigma-Aldrich)) on ice for 15 min with sporadic vortexing. Extracts were cleared by centrifugation (16,100*g* for 10 min at 4 °C). m^7^GTP-Agarose beads (γ-aminohexyl-m7GTP-agarose; Jena Biosciences, Jena, Germany) were equilibrated in buffer C (50 mM MOPS KOH (pH 7.4), 100 mM NaCl, 50 mM NaF, 0.5 mM EDTA, 0.5 mM EGTA, 7 mM β-mercaptoethanol, 2 mM benzamidine or 0.5 mM PMSF, 1 mM Na_3_VO_4_ and 0.1 mM GTP (Sigma-Aldrich)). After equilibration, lysates were diluted (∼500 μg of total cell protein) to 1 ml with buffer C (in a 2-ml tube) and incubated with equilibrated m^7^GTP-Agarose beads (∼50 μl of 50% slurry) for 20 min at 4 °C end-over-end rotation. Ten per cent of the lysate was used as the input. The beads were collected by centrifugation (500*g* for 5 min at 4 °C) and washed four times with 1.5 ml of buffer C. Bound proteins were eluted with 0.2 mM m^7^GTP, resuspended in SDS–PAGE loading buffer and analysed by western blotting along with the inputs. Experiments were performed in independent duplicate.

### Co-immunoprecipitations, sqRT–PCR and qRT–PCR analyses

Raptor immunopreciptations were carried out using an anti-raptor antibody (Millipore). HEK293E cells were serum starved overnight and then treated as described in figure legends. Cells were collected in CHAPS buffer (0.2% CHAPS, 40 mM HEPES (pH 7.4), 120 mM NaCl, 1 mM EDTA, 10 mM pyrophosphate, 10 mM β-glycerophosphate, 50 mM NaF, 1 mM DTT). Immunoprecipitation was performed in the presence of 0.5 mg ml^−1^ reversible crosslinker 3,3′-dithiobis (sulfosuccinimidylpropionate) DTTSP (Life Technologies). Two microlitres of anti-raptor antibody (Millipore, 1:500) and 30 μl of protein G-sepharose 50% slurry (Millipore) were equilibrated in CHAPS buffer and incubated in 100 μl of CHAPS buffer for 30 min at 4 °C, washed followed by incubation with 1 mg of the lysates for 4 h at 4 °C, with end-to-end rotation. Beads were washed four times with 1 ml of ice-cold CHAPS buffer and collected by centrifugation (500*g* for 2 min at 4 °C). Fifty microlitres of beads were resuspended in the sample buffer, boiled and analysed by western blotting.

For endogenous eIF2β immunoprecipitations, cells were collected by scraping, washed three times in ice-cold PBS (1,200 r.p.m. for 5 min at 4 °C) and lysed in NET-2 buffer (50 mM Tris (pH 7.4), 150 mM NaCl, 2 mM MgOAc, 0.1% NP-40, 1 mM DTT, 1 × EDTA-free protease inhibitors (Roche)) by 3 × 10-s bursts using microtip at power 6 on ice. The lysates were spun for 10 min/16,100*g* at 4 °C. An amount of 1 mg ml^−1^ of the protein was incubated with 40 μl of the NET-2 buffer equilibrated protein A sepharose beads (Millipore; 30 min at 4 ° C; end-over-end rotation) on which the supernatant was split in two, set at 500 μg ml^−1^ and incubated for 2 h at 4 ° C (end-over-end rotation) with either eIF2β antibody (P-3) #sc-9978 from Santa Cruz Biotechnologies or the appropriate IgG1 control (#M5284; Sigma; 2 μg of the antibody/IgG per 100–500 μg of total cell protein). On the incubation with the antibody, protein A sepharose beads were added (∼30 μl μg^−1^ of antibody), and the incubation was carried out for the additional 2 h at 4 °C. After the immunoprecipitated beads were washed one time with NET-2 buffer containing 300 mM NaCl and five times with NET-2 buffer containing 150 mM NaCl. Beads were eluted in Laemmli buffer by boiling and eluates were analysed by western blotting.

Anti-FLAG immunopreciptiations were carried out using anti-FLAG(M2) #F3165 and the same concentration of isotype-matched mouse IgG1 antibody #M5284 as a control (both from Sigma). Two micrograms of the antibody/IgG per 100–500 μg of total cell protein were used. HEK293E cells were seeded in a 15-cm Petri dish and collected at 80% confluency in 1.5 ml eIF2-IP buffer (20 mM Tris HCl (pH 7.5), 100 mM KCl, 5 mM MgCl_2_, 0.1 mM EDTA, 5 mM EGTA, 1 mM DTT, 20 mM NaF, 0.1 mM Na_3_VO_4_, 20 mM β-glycerophosphate, 0.3% CHAPS). FLAG-eIF2β variants were immunoprecipitated using 10 μl FLAG-agarose beads (Sigma-Aldrich), previously equilibrated in eIF2-IP buffer, for 90 min at 4 °C. Immunoprecipitated material was washed twice in 1 ml of eIF2-IP buffer (4,000 r.p.m. for 1 min at 4 °C). FLAG-eIF2β was eluted three times with FLAG-eluting peptide at 4 °C according to the manufacturer's instructions. To monitor eIF2:tRNA binding, half of the immunoprecipitated material was analysed by western blotting and half was used to extract RNA using Trizol. Analysis of tRNA_i_^Met^ and tRNA^Lys^ was performed by semi-quantitative reverse transcription–PCR (sqRT–PCR) using OneStep RT–PCR kit (Qiagen) and quantitative reverse transcription–PCR (qRT–PCR) using Power SYBR Green RNA-to-Ct 1-Step Kit (Applied Bioscience) according to the manufacturer's instruction. Following primers were used:

tRNA_i_^Met^_; Amplicon: 71

tRNA_i_^Met^_Fwd: 5′-AGCAGAGTGGCGCAGCGGAAGCGTGCT-3′

tRNA_i_^Met^_Rev: 5′-TAGCAGAGGATGGTTTAGATCCATC-3′

tRNA^Lys^_; Amplicon: 72

tRNA^Lys^_Fwd: 5′-GCCCGGATAGCTCAGTCGGTAGA-3′

tRNA^Lys^_Rev: 5′-CGCCCGAACAGGGACTTGAACC-3′

sqRT–PCR reactions were carried out using the following conditions: annealing *T*=60 °C for 30 s, denaturation *T*=94 ° C for 1 min and elongation *T*=72 °C for 1 min for 30 cycles. qRT–PCR reactions were carried out under the following conditions: reverse transcription: 48 °C for 30 min; activation of DNA polymerase: 95 °C for 10 min; 40 cycles: denature 95 °C for 15 s; anneal/extend 60 °C for 1 min. All immunoprecipitation/PCR experiments were carried out at least in independent duplicates.

### Proliferation assay

HEK293E cells (1 × 10^4^) expressing eIF2β variants, in which endogenous eIF2β was depleted by shRNA (Supplementary Fig. 4b), were seeded in a 96-well plate and treated as indicated in figure legends. For amino-acid deprivation experiments, 5 × 10^4^ cells were seeded in a 96-well plate and maintained in DMEM without amino acids (Gibco) supplemented with 10% dialysed serum for 6 and 18 h (Invitrogen). Proliferation rate was determined using Cell Proliferation ELISA BrdU kit (Roche). Absorbance at 370 nm (reference wavelength 492 nm) was measured using a Benchmark Plus microplate reader (Bio-Rad). The experiments were performed in independent triplicate (*n*=3), with four technical replicates per biological replicate.

### Circular dichroism

Far-ultraviolet circular dichroism spectra of the eIF2β WT and S(2,67)A, TOS and S(2,67)D eIF2β mutants (4 μg in 100 μl of 10 mM phosphate buffer (pH 7.2), 50 mM NaCl) purified as described in the ‘Constructs and recombinant proteins' section were continuously detected from 197 to 250 nm using Chirascan CD Spectrometer (Applied Photophysics). The measurements were carried out in using a 1-mm path-length cuvette (Hellma) at room temperature using a 1-nm bandwidth. For each sample, two spectra were collected and averaged. The spectral contribution of the buffer was corrected for by subtraction.

### Identification of TOS motifs

TOS motifs (F E/D/V M/I/L D/E/V I/E/L) were identified in RefSeq proteins using pattern matching^70^ in both mouse and human proteins. For genes encoding for proteins with multiple isoforms containing a TOS motif, only one isoform was reported. Human proteins with TOS motifs were reported with a comparison with TOS motifs in mouse proteins (human and mouse proteins were linked using gene symbols).

### Separation of cellular extracts on 15–35% sucrose gradients

For ribosome profiles shown in Supplementary Fig. 3e, 80% confluent HEK293E cells that stably express exogenous FLAG-eIF2β variants and are depleted of endogenous eIF2β (Supplementary Fig. 4B) were collected in buffer containing 30 mM Tris HCl (pH 7.5), 100 mM NaCl, 2.5 mM MgCl_2_, 0.1% Na-deoxycholate, 100 μg ml^−1^ cycloheximide, 1 mM DTT, 20 mM NaF, 0.1 mM Na_3_VO_4_ and 20 mM β-glycerophosphate. Five ODs (at 254 nm) were loaded onto 15–35% sucrose gradients buffered with 20 mM HEPES (pH 7.6), 100 mM KCl, 5 mM MgCl_2_, 100 μg ml^−1^ cycloheximide, 1 × protease inhibitor cocktail (EDTA free), 100 U ml^−1^ RNase inhibitor (Ambion) and subjected to ultracentrifugation (SW41 rotor; Beckman 39,000, 4 h 30 min at 4 °C). Absorbance profiles were detected as described in the section entitled ‘Genome-wide polysome profiling and qRT–PCR'. Area under the monosome (80S) peak areas were integrated using ImageJ, and presented as 80S vector control versus indicated eIF2β variants.

## Additional information

**Accession codes:** Raw and processed data are available at the Gene Expression Omnibus (GEO; GSE76766).

**How to cite this article:** Gandin, V. *et al*. mTORC1 and CK2 coordinate ternary and eIF4F complex assembly. *Nat. Commun.* 7:11127 doi: 10.1038/ncomms11127 (2016).

## Supplementary Material

Supplementary Information Supplementary Figures 1-10
