# Supplementary material for: mTORC1 and CK2 coordinate ternary and eIF4F complex assembly
Source: Nat Commun. 2016 Apr 4;7:11127. doi: 10.1038/ncomms11127 (PMC4822005; doi:10.1038/ncomms11127)
Supplement: Supplementary Information — Supplementary Figures 1-10 [file ncomms11127-s1.pdf]

mTORC1 and CK2 coordinate ternary and eIF4F complex assembly

Valentina Gandin[1,2,3,4]*, Laia Masvidal[5]*, Marie Cargnello[1,2,3,4]*, Laszlo Gyenis[6], Shannon McLaughlan[1,2,3,4], Yutian Cai[1,2,3,4], Clara Tenkerian[1,6], Masahiro Morita[3], Preetika Balanathan[7], Olivier Jean-Jean[8], Vuk Stambolic[9], Matthias Trost[10], Luc Furic[7], Louise Larose[11], Antonis E. Koromilas[1,4,6], Katsura Asano[12], David Litchfield[6], Ola Larsson[5] and Ivan Topisirovic[1,2,3,4]****

Supplementary material containing **Supplementary figures 1-10**.

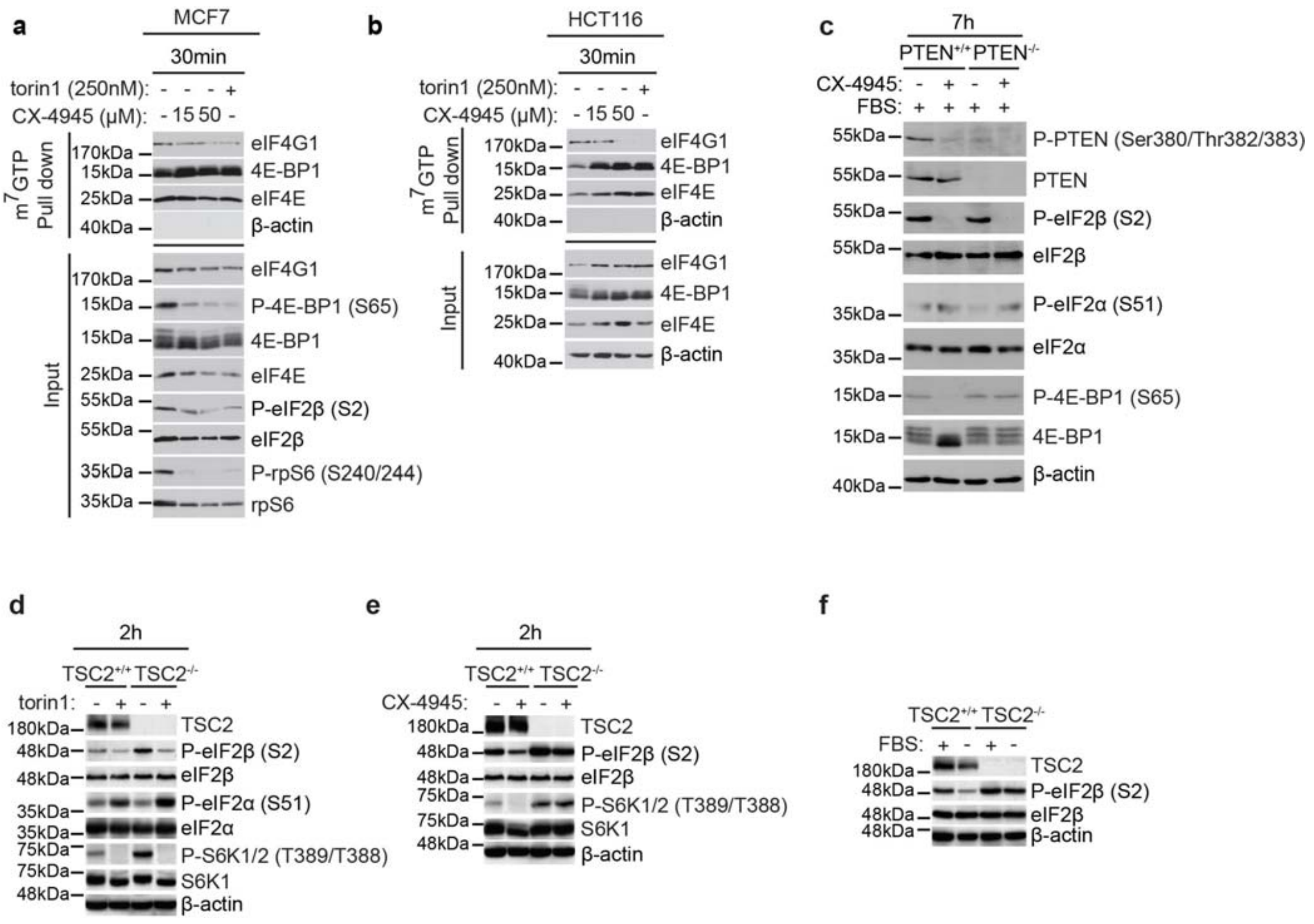

Supplementary Fig. 1 CK2 inhibition impairs the eIF4F complex assembly, and reduces eIF2β phosphorylation, whereas loss of upstream negative regulators of mTOR attenuates the effects of CK2 inhibitors on eIF2β phosphorylation (a-b) MCF7 **(a)** or HCT116 **(b)** cells were treated with indicated concentrations of CX-4945 and torin1 for 30 minutes, after which cells were lysed and lysates were subjected to a m^7GTP cap-pull down assay. Levels and phosphorylation status of indicated proteins in the pulled down material and inputs (10%) were determined by Western blotting. β-actin served as a loading control for the inputs and to exclude possible contamination of m^7GTP cap-pull downs. **(c)** HCT116 $PTEN^{+/+}$ and $PTEN^{-/-}$ maintained in 10% serum (FBS) were treated with a vehicle (DMSO) or 50μM CX-4945 for 7 hours. (**d-f**) $TSC2^{+/+}$ and $TSC2^{-/-}$ mouse embryonic fibroblasts (MEFs) were starved for 24 hours in 0.1% serum (FBS) and stimulated with 10% FBS in the presence of a vehicle (DMSO), 250 nM torin1 **(d)**, or 50μM CX-4945 **(e)** for 2 hours. In addition, cells were maintained in 10% or 0.1% serum (FBS) for 24 hours without treatments (**f**). Phosphorylation status and expression of indicated proteins were monitored by Western blotting. β-actin served as a loading control for the inputs**.** Experiments are representative of at least 2 independent experiments. Quantification was performed using densitometry (**Suppl. Fig 9**).

a

Human Symbol	Human RefSeq	Human SeqPos	Mouse Symbol	Mouse RefSeq	Mouse SeqPos
EIF2S2	NP_003899	FDIDE [89,93]	Eif2s2	NP_080306	FDIDE [89,93]
RPS6KB2	NP_003943	FDLDL [5,9]	RpS6kb2	NP_067460	FDLDL [5,9]
EIF4A1	NP_001407	FVLDE [179,183]	Eif4a1	NP_001152847	FVLDE [179,183]
EIF3C	NP_001186071	FELDL [811,815]	Eif3c	NP_666312	FELDL [809,813]
EEF1G	NP_001395	FVLDE [288,292]	Eef1g	NP_080283	FVLDE [288,292]
EIF4A2	NP_001958	FVLDE [180,184]	Eif4a2	NP_001116510	FVLDE [180,184]
EIF4EBP1	NP_004086	FEMDI [114,118]	Eif4ebp1	NP_031944	FEMDI [113,117]
EIF4EBP2	NP_004087	FEMDI [116,120]	Eif4ebp2	NP_034254	FEMDI [116,120]
EIF4EBP3	NP_003723	FEMDI [96,100]			
RPS6KB1	NP_003152	FDIDL [28,32]	Rps6kb1	NP_082535	FDIDL [28,32]

b

Human_(S2-p) 1 MsGDEMIFD 9
Mouse_(S2-p) 1 MsGDEMIFD 9
Rat_(S2-p) 1 MsGDEMIFD 9
Rabbit_(S2-p) 1 MsGDEMIFD 9

Human_(S67-p) 60 DTRKKDAsDDLDDLN 74
Mouse_(S67-p) 60 DSRKKDAsDDLDDLN 74
Rat_(S67-p) 60 DSRKKDAsDDLDDLN 74
Rabbit_(S67-p) 60 DSRKKDAsDDLDDLN 74

Human_TOS 89 FDIDE 93
Mouse_TOS 89 FDIDE 93
Rat_TOS 89 FDIDE 93
Rabbit_TOS 89 FDIDE 93

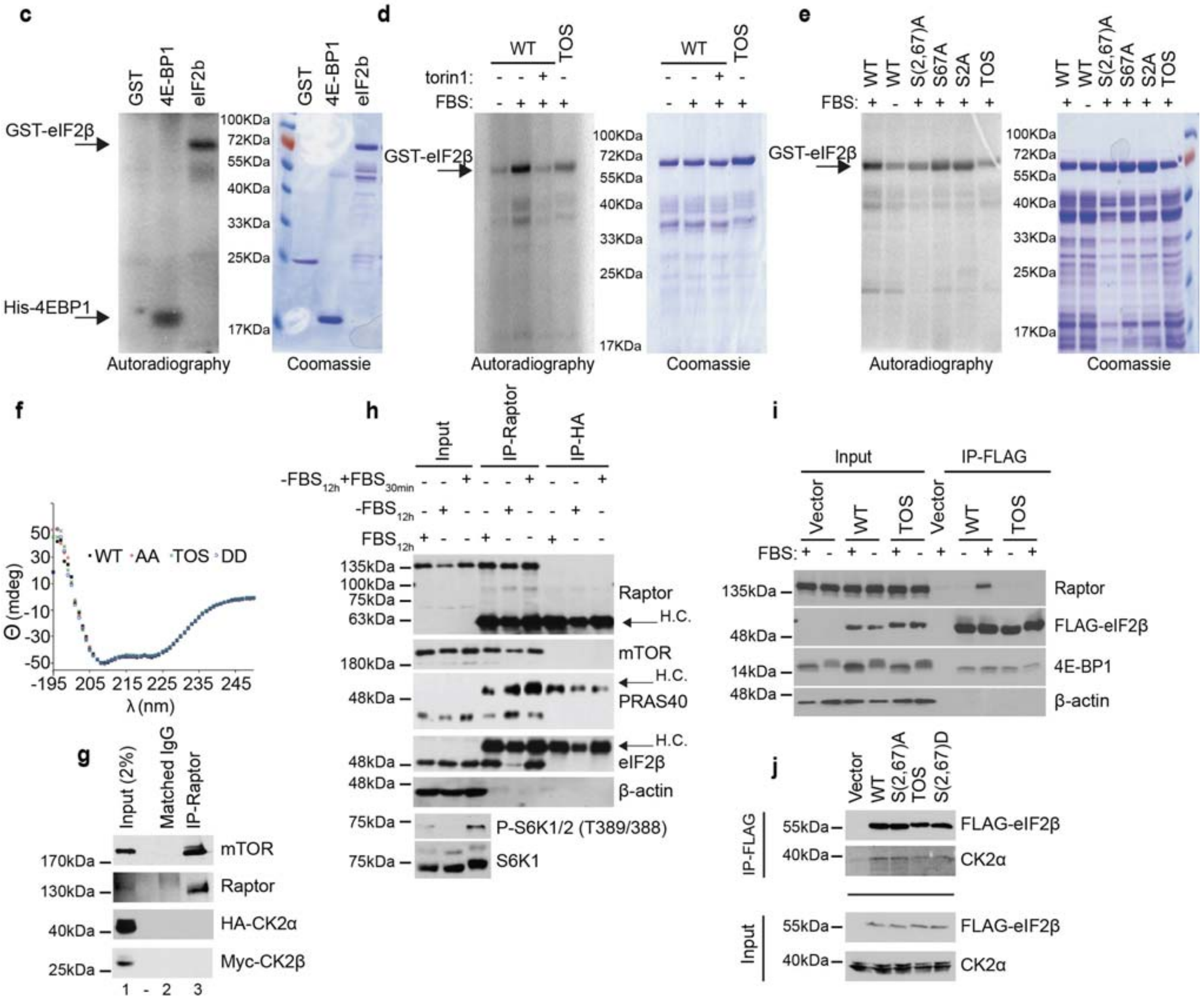

Supplementary Fig 2 (legend below)

Supplementary Fig. 2 mTORC1 phosphorylates eIF2β on serines 2 and 67 (a) Putative TOS motifs of eIF2β and known mTORC1 substrates and their position in the proteins are indicated in the table. (**b**) Alignment of eIF2β regions containing S2 and S67 phospho-acceptor sites [(S2-p) and (S67-p); shown in red] and TOS motif in indicated species. **(c-e)** *In vitro* mTORC1 kinase assay (left panel) was carried out using recombinant wild type (WT) or indicated eIF2β mutants. mTORC1 was isolated by HA-immunoprecipitation from HEK293E cells transfected with HA-Raptor (see methods) that were serum starved for 16h and then stimulated with 10% serum in the presence of a vehicle (DMSO) or torin1 (250 nM) for 30 minutes. Coomassie staining indicates the quantity of eIF2β variants used in mTORC1 kinase assay. **(f)** Far-UV circular dichroism (CD) spectra of indicated recombinant eIF2β proteins. Θ (ellipticity in millidegrees); λ (wavelength in nanometers). **(g)** U20S cells overexpressing HA-CK2a and myc-CK2b were serum starved for 16h and stimulated with serum (10%) for 30 min followed by immunoprecipitation with an anti-raptor antibody. (**h**) HEK293E cells were grown in the absence ($-FBS_{12h}$) or presence ($+FBS_{12h}$) of 10% fetal bovine serum (FBS) for 12h. In addition, serum starved cells were stimulated with 10% FBS for 30 minutes ($-FBS_{12h}+FBS_{30min}$). Lysates were immunoprecipitated using an anti-raptor antibody. Isotype matched anti-HA antibody served as a negative control. Immunoprecipitated material (25%) and inputs (10%) were analyzed by Western blotting using indicated antibodies. β-actin served as a loading control for the inputs and to exclude possible contamination due to non-specific interactions in the immunoprecipitation reactions, whereas S6K1/2 phosphorylation in the inputs was used to determine mTOR activity. H.C.- heavy chains. **(i)** HEK293E cells were transfected with the indicated constructs, serum starved for 16h and stimulated with 10% serum (FBS) for 30min before FLAG-immunoprecipitation. Immunoprecipitated material (25%) and inputs (10%) were analyzed by Western blotting using the indicated antibodies. To capture native mTOR complexes, immunoprecipitations in **(h, i)** were carried out in the presence of the cross-linker 3,3′-dithiobis (sulfosuccinimidylpropionate) DTTSP (see methods). **(j)** HEK293E cells expressing indicated eIF2β constructs were immunoprecipitated with an anti-CK2α antibody and the levels of indicated proteins in inputs (10%) and immunoprecipitates (25%) were determined by Western blotting. Experiments in this panel were repeated at least 2 times independently, and representative data are shown.

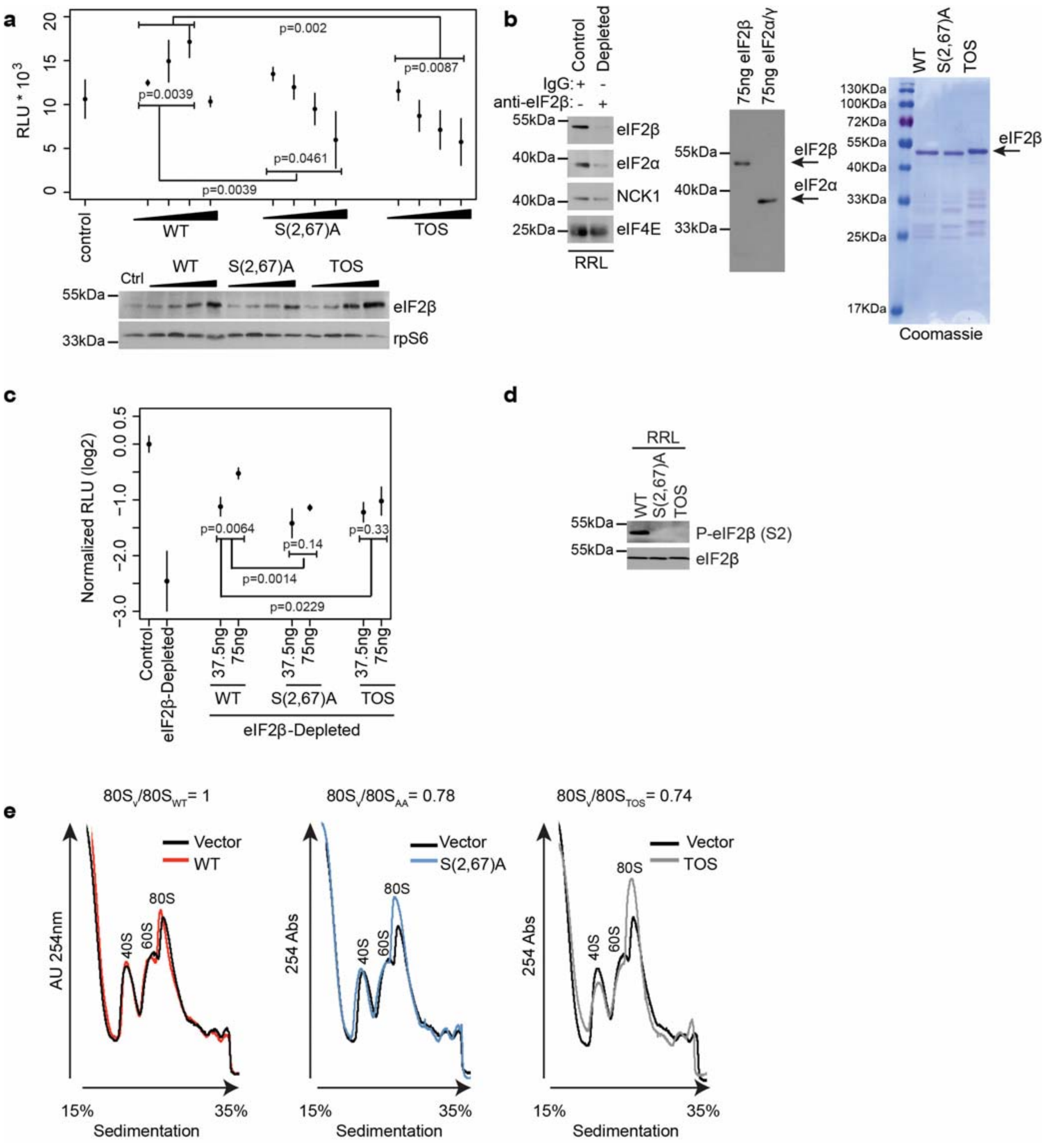

Supplementary Fig. 3. (legend below)

Suppl. Fig. 3. eIF2β phosphorylation stimulates mRNA translation. (a-c) Translation of m^7GpppG-Renilla luciferase-poly(A) mRNA (50 ng) was monitored in RRL supplemented with increasing amounts of eIF2β WT, S(2,67)A or TOS mutant (**a**) or in RRLs in which eIF2 was immunodepleted using an anti-eIF2β antibody and rescued with recombinant eIF2α/γ and equimolar amounts of recombinant eIF2β WT, S(2,67)A or TOS mutant **(b-c)**. Translation of m^7GpppG-Renilla luciferase-poly(A) mRNA was monitored by luciferase assay **(a, c)**. Experiments were performed in independent triplicate and data are presented as log2 transformed and normalized (per replicate and the mean of the control) means and SDs. P-values from a 1-way ANOVA for an amount effect (for WT, S(2,67)A or TOS mutant separately) and from 2-way ANOVAs comparing treatments are indicated. Levels of endogenous and recombinant proteins were monitored by Western blotting using indicated antibodies. rpS6 (**a**) and eIF4E **(b)** were used as loading control. Coomassie staining was used to determine the levels and purity of recombinant eIF2β proteins **(b)**. **(d)** Phosphorylation status and levels of indicated recombinant eIF2β variants in rabbit reticulocyte lysates (RRL) were monitored by Western blotting**. (e)** HEK293E cells expressing indicated eIF2β constructs were serum starved for 16h, and then serum stimulated for 4h after which cells were lysed and cytosolic extracts were sedimented on 15-35% sucrose by ultracentrifugation. Position of 40S and 60S ribosomal subunits as well as 80S monosome are denoted in the absorbance profiles (254nm). 80S ratios between cells expressing indicated eIF2β constructs and control cells were calculated by dividing areas under corresponding 80S peaks.

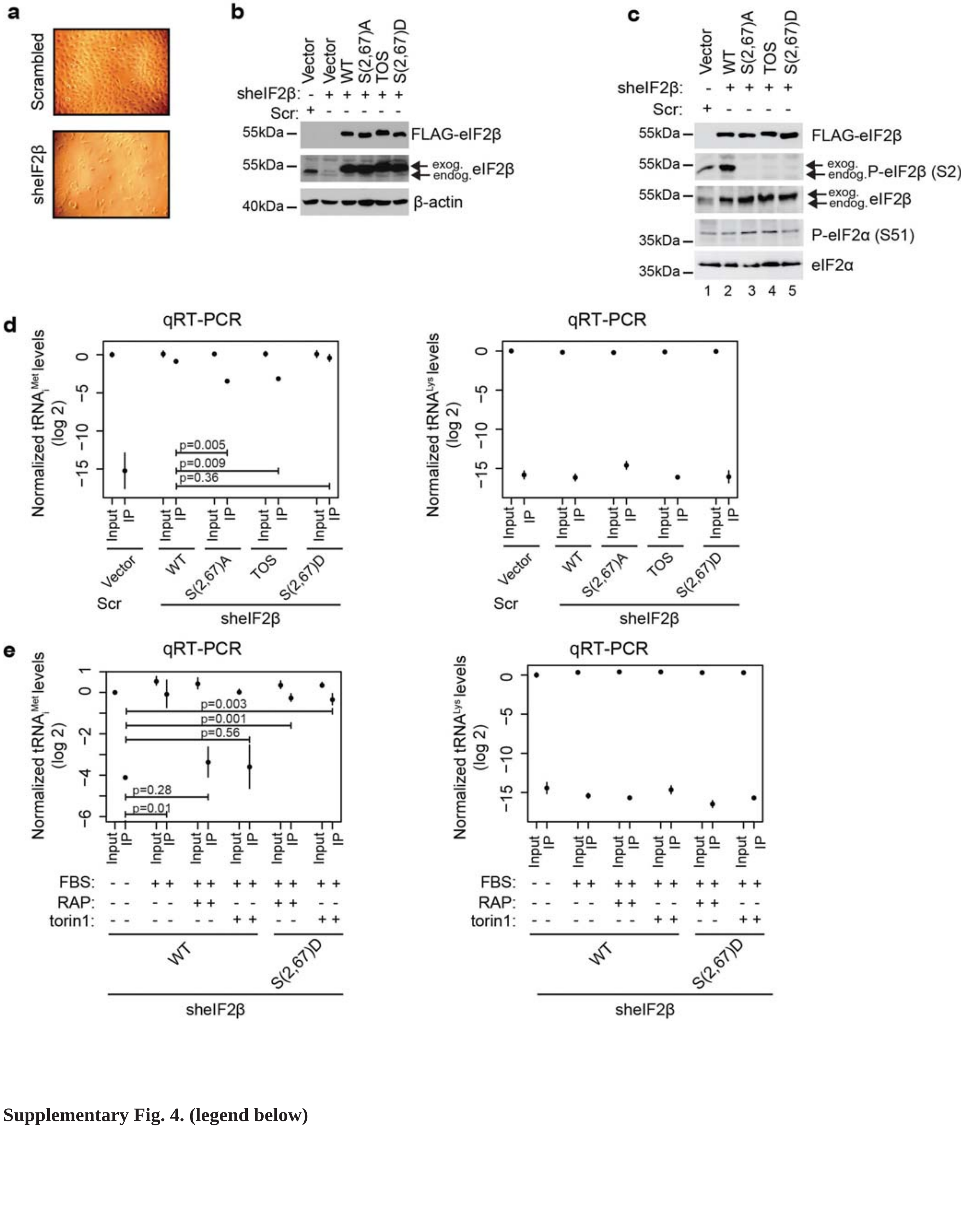

Supplementary Fig. 4. (legend below)

Supplementary Fig. 4. eIF2β depletion has deleterious effects on HEK293E cells, while eIF2β phosphorylation increases eIF2:tRNA$_i^{Met}$ association (a) HEK293E cells were depleted of eIF2β or infected with scrambled control shRNA. Phase/contrast images show that upon 48h of eIF2β depletion the vast majority of the cells were dead. **(b)** To overcome cell toxicity caused by eIF2β depletion, HEK293E cells were first infected with empty vector or indicated eIF2β constructs and 72 hours later with scrambled control (Scr) or eIF2β shRNA (shRNAeIF2β), respectively. In this way cells in lanes 3-6 express indicated exogenous FLAG-tagged eIF2β variants and are depleted of endogenous eIF2β, whereas control cells (infected with vector+scrambled shRNA) express endogenous eIF2β. Levels of indicated proteins were determined by Western blotting. β-actin served as a loading control. **(c)** Levels and the phosphorylation status of indicated proteins in cells described in **(b)** were monitored by Western blotting. Total eIF2α levels served as a loading control. Experiments were carried out 3 times independently and representative data are shown. Were appropriate densitometry was performed (**Suppl. Fig. 9**). **(b-c)** Endogenous (endog.) and exogenous (exog.) variants of eIF2β proteins detected by an anti-eIF2β or anti-phopsho Ser2 eIF2β antibody are shown **(d)** Cells described in **(b)** were serum starved for 16h, stimulated with serum (10%) for 30 min and subjected to FLAG-immunoprecipitation as in **Fig 4a**. **(e)** Cells expressing WT or S(2,67)D eIF2β mutant were serum starved for 16h and then stimulated with 10% serum (FBS) in the presence of a vehicle (DMSO), rapamycin (50 nM) or torin1 (250 nM) for 30 min as in **Fig. 4b**. Lysates were immunoprecipitated with an anti-FLAG antibody. **(d-e)** The amount of tRNA$_i^{Met}$ in the immunoprecipitated material was monitored by quantitative reverse-transcription PCR (qRT-PCR). tRNA^{Lys} was used as a negative control. Experiments were carried out in independent duplicate consisting of 3 technical replicates each. Means from technical replicates were log2 transformed, normalized per replicate and to the mean of the control, and are shown as mean and standard deviations. P-values from 1-way ANOVAs are indicated.

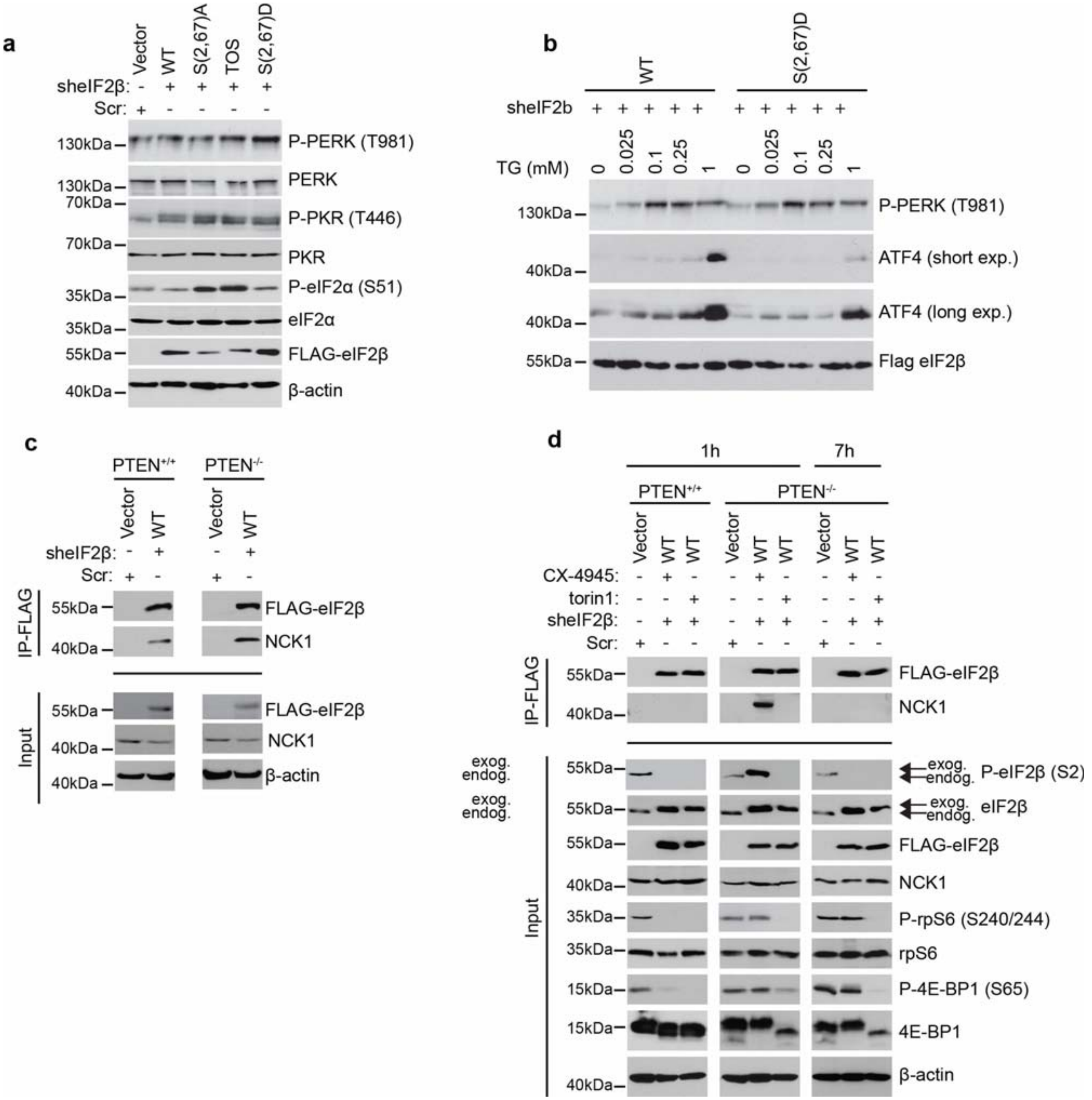

Supplementary Fig. 5 PERK and PKR do not appear to act as major mediators of the effects of eIF2β on eIF2α phosphorylation, whereas the effects of CK2 inhibition on eIF2β phosphorylation and eIF2β:NCK1 association are attenuated in the cells lacking PTEN. (a) Expression and phosphorylation status of indicated proteins in HEK293E cells expressing eIF2β constructs that were depleted of endogenous eIF2β by shRNA (sheIF2β) and HEK293E cells infected with empty vector and scrambled (Scr) shRNA (control) **(Supplementary Fig. 4b)** were monitored by Western blotting using indicated antibodies. β-actin served as a loading control. **(b)** HEK293E cells expressing WT and S(2,67)D eIF2β mutant in which endogenous eIF2β was depleted by shRNA were treated with increasing concentrations of thapsigargin for 4 hours and the expression and phosphorylation status of indicated proteins were determined by Western blotting. **(c)** HCT116 $PTEN^{+/+}$ and $PTEN^{-/-}$ cells transfected with an empty vector/scrambled (Scr) shRNA (control) or eIF2β shRNA and shRNA-insensitive FLAG-WT eIF2β were immunoprecipitated with an anti-FLAG antibody and the amount of indicated proteins in the inputs (10%) and immunoprecipitates (25%) were determined by Western blotting. **(d)** Cells described in **(c)** were treated with torin1 (250nM) or CX-4945 (50μM) for the indicated time periods, after which anti-FLAG antibody immunoprecipitations were carried out. **(c-d)** β-actin served as a loading control in the inputs. Levels and the phosphorylation status of indicated proteins in the inputs (10%) and immunoprecipitates were determined by Western blotting. Endogenous (endog.) and exogenous (exog.) variants of eIF2β proteins detected by an anti-eIF2β or anti-phospho Ser2 eIF2β antibody are indicated. Experiments were repeated twice independently and representative results are shown.

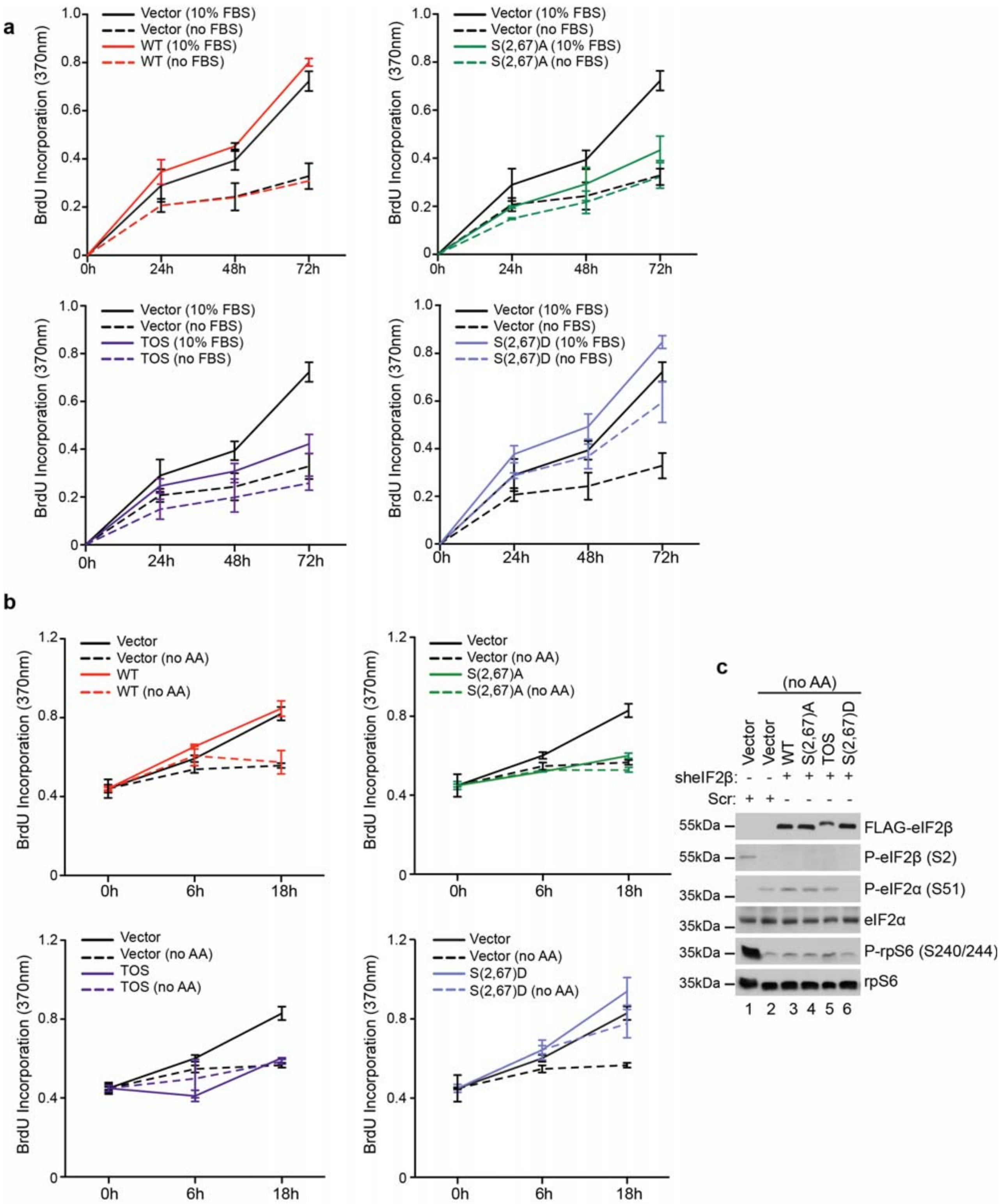

Supplementary Fig. 6. eIF2β phosphorylation promotes cell proliferation under conditions when mTOR signaling is inhibited. (a) HEK293E cells expressing eIF2β constructs in which endogenous eIF2β was depleted by shRNA (**Supplementary Fig 4b**) were maintained in 10% or 0.5% serum (FBS) for indicated time and proliferation was monitored by BrdU incorporation. Results are presented as mean absorbance at 370nm +/- SD from 3 independent experiments **(b)** Cells described in **(a)** were maintained in absence of amino acids for indicated time points and proliferation was measured as in **(a)**. **(c)** Effects of amino acid deprivation on the expression and phosphorylation status of indicated proteins were determined by Western blotting. Total rpS6 was used as loading control. Experiments were repeated 2 times independently, and representative results are shown.

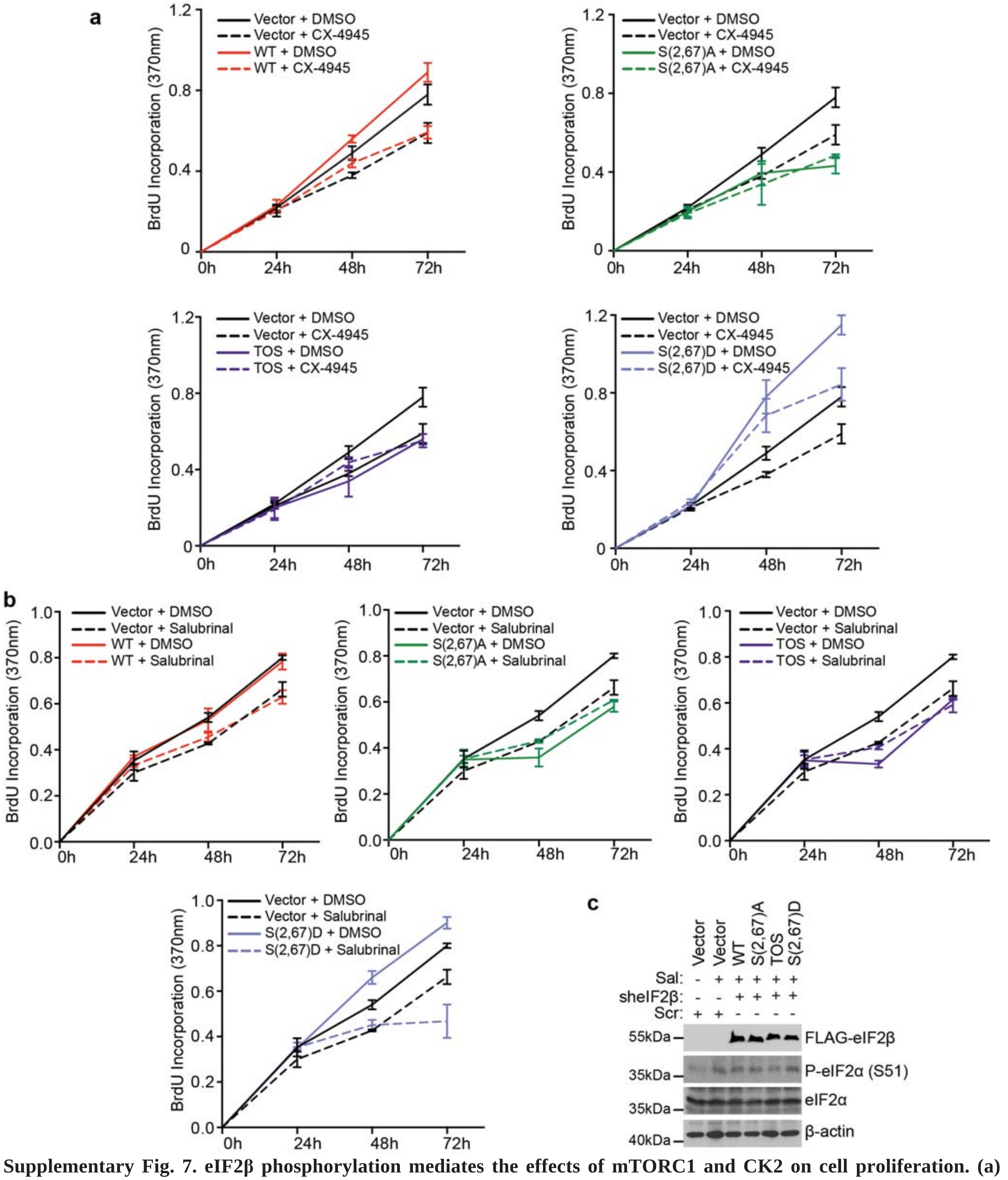

Supplementary Fig. 7. eIF2β phosphorylation mediates the effects of mTORC1 and CK2 on cell proliferation. (a) HEK293E cells expressing the indicated eIF2β variants in which endogenous eIF2β was depleted by shRNA (**Supplementary Fig. 4b**) were treated with a vehicle (DMSO) or CX-4945 (10mM) for indicated times. **(b)** Cells described in **(a)** were incubated with a vehicle (DMSO) or salubrinal (1μM) for indicated times. **(a-b)** Proliferation was measured by BrdU incorporation, and the results are presented as mean absorbance +/- SD from triplicate experiments. **(c)** Expression and the phosphorylation status of indicated proteins were measured by Western blotting. β-actin served as a loading control. Representative blots of 2 independent experiments are shown.

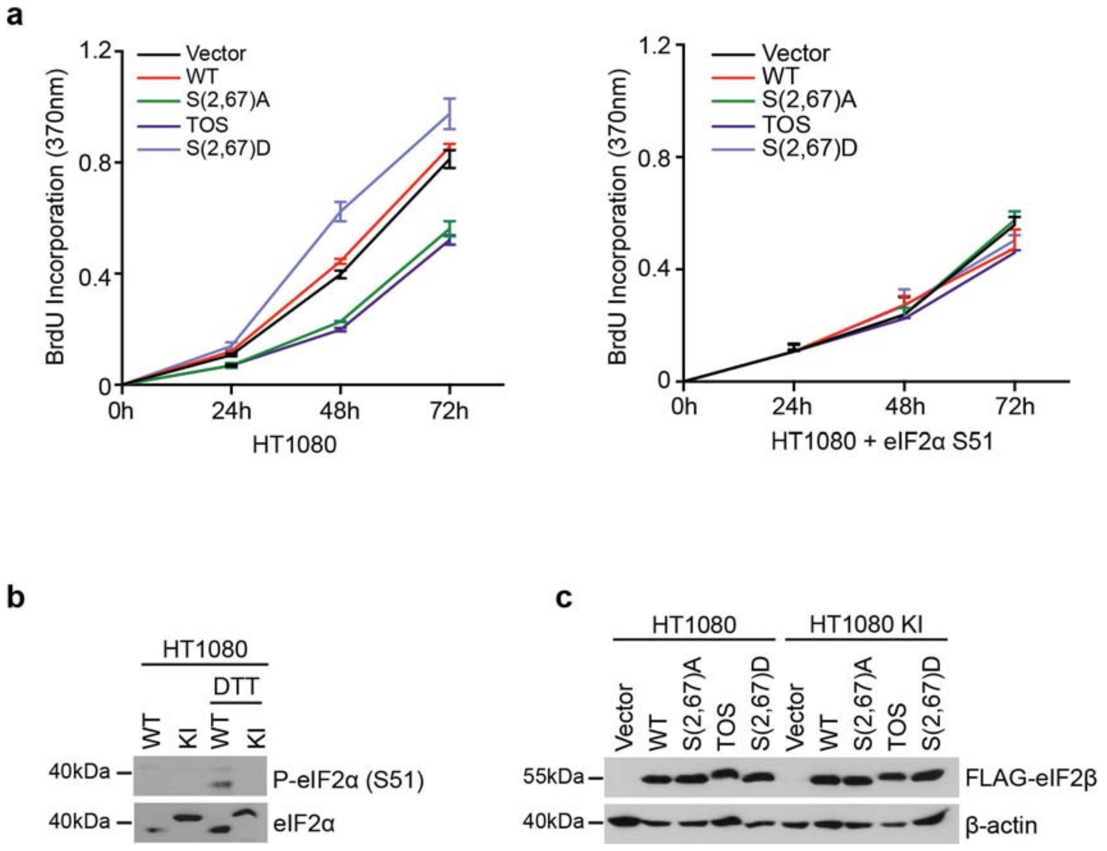

Suppl. Fig. 8. eIF2α phosphorylation mediates the effects of eIF2β phosphorylation on cell proliferation (a) HT1080 WT or HT1080 eIF2α S51A mutant (KI) cells expressing the indicated eIF2β constructs were maintained in 10% serum and proliferation was measured at indicated time points by BrdU incorporation. Results are represented as mean absorbance +/- SD from independent triplicate experiments. **(b-c)** Expression levels and phosphorylation status of indicated proteins in HT1080 WT or HT1080 KI cells were monitored by Western blotting. As a control, cells were treated with DTT (2mM) for 1h to induce eIF2α phosphorylation. Representative blots of 2 independent experiments are presented.

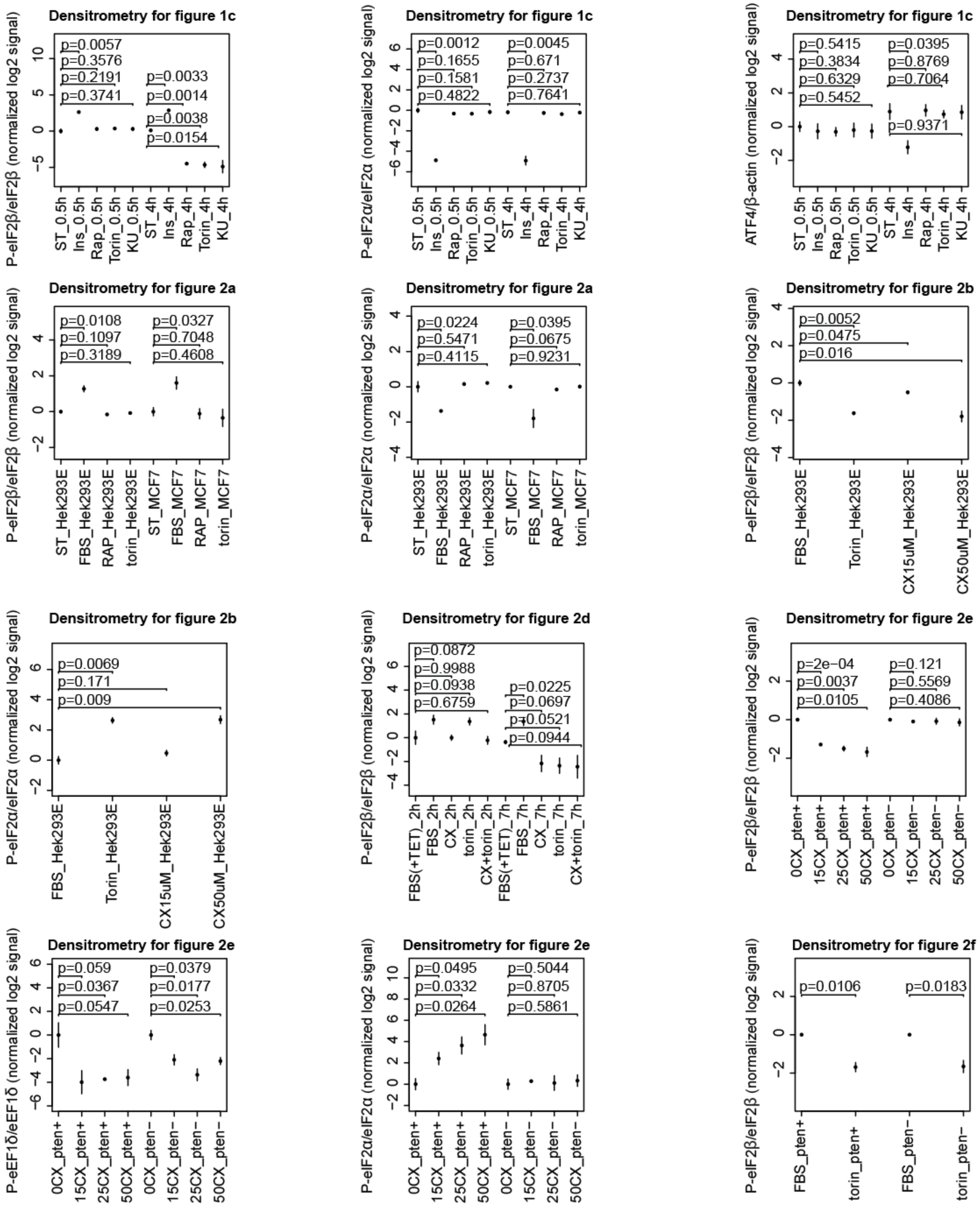

Supplementary Fig. 9. (continued below; legend on page 15)

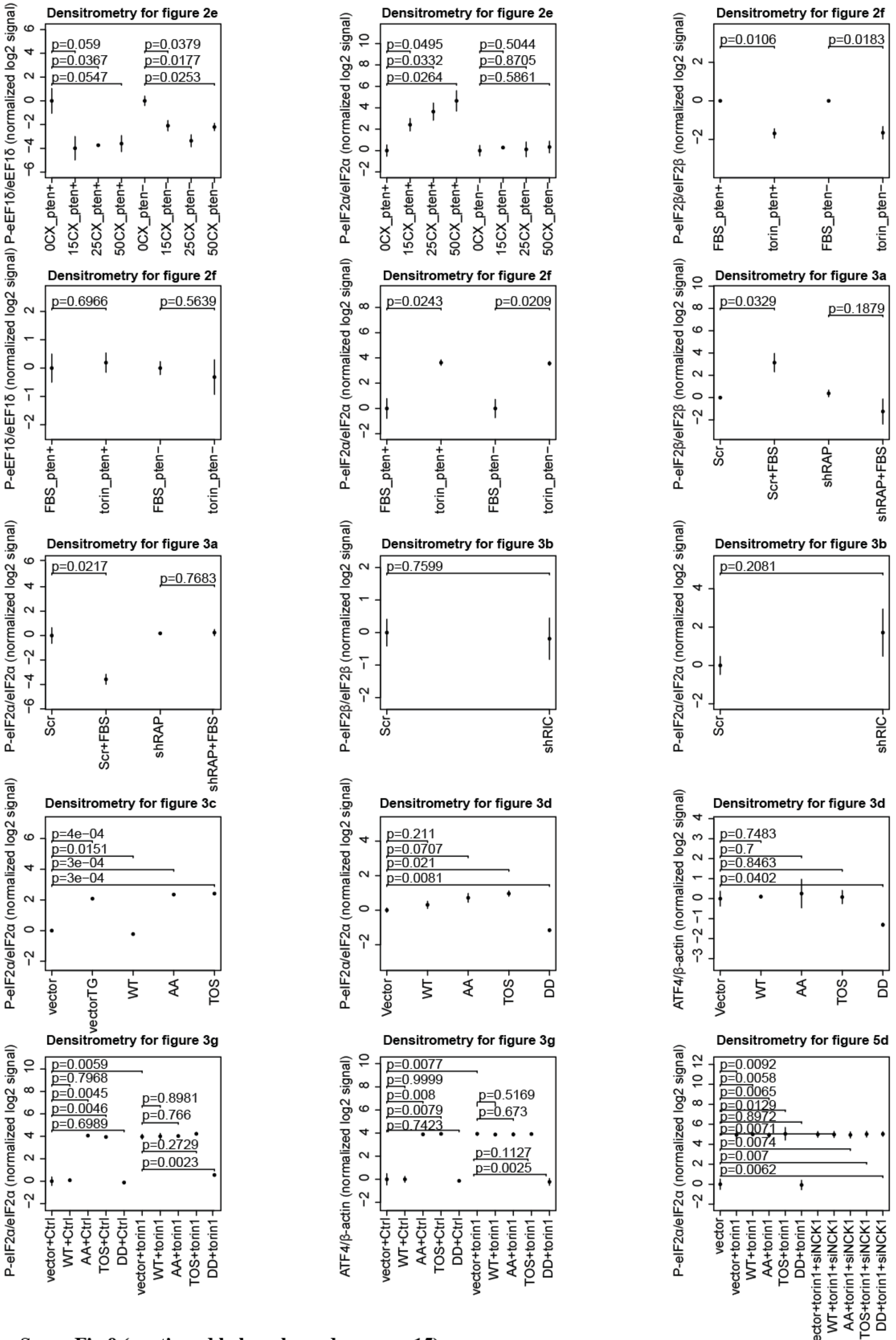

Supp. Fig 9 (continued below; legend on page 15)

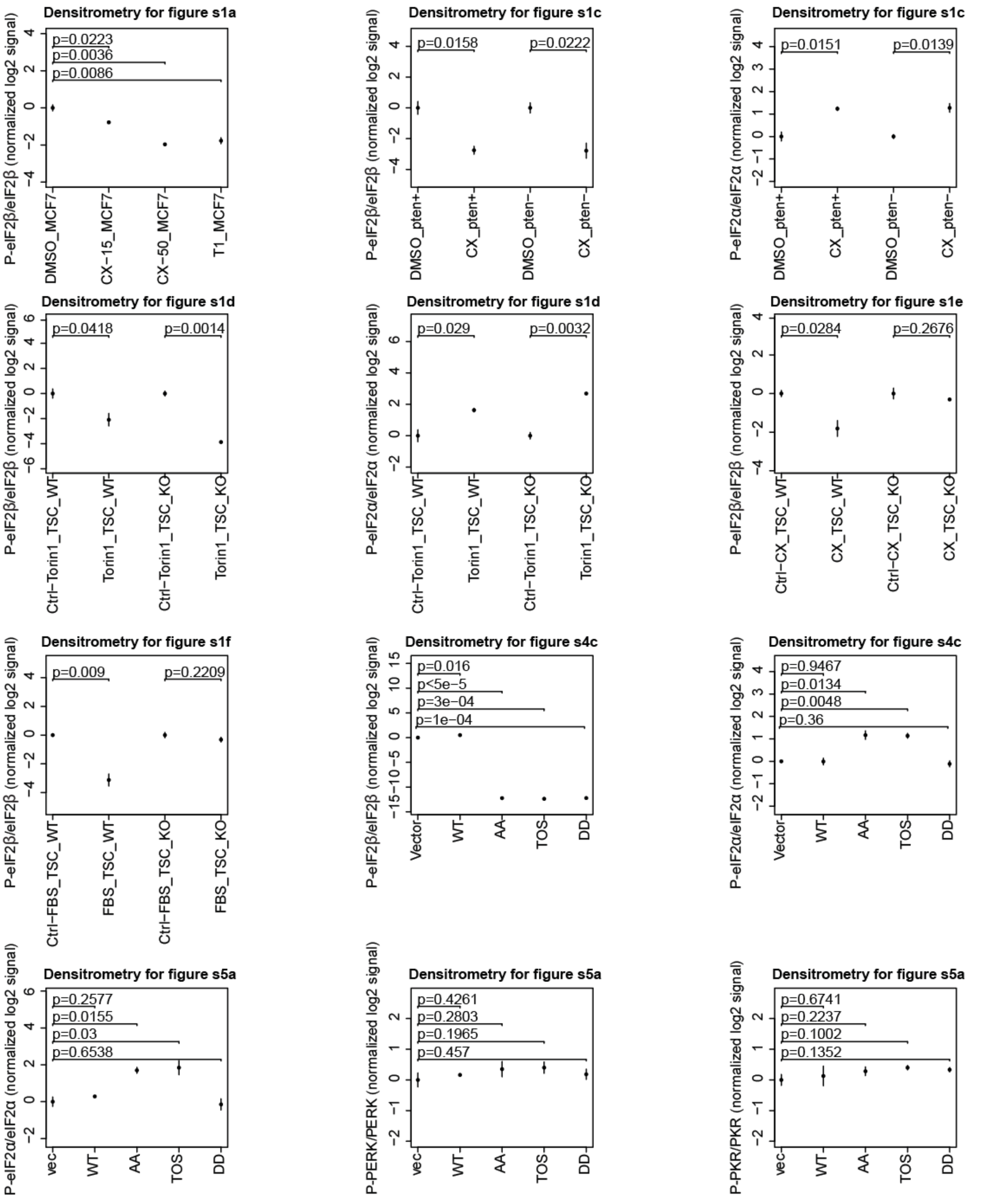

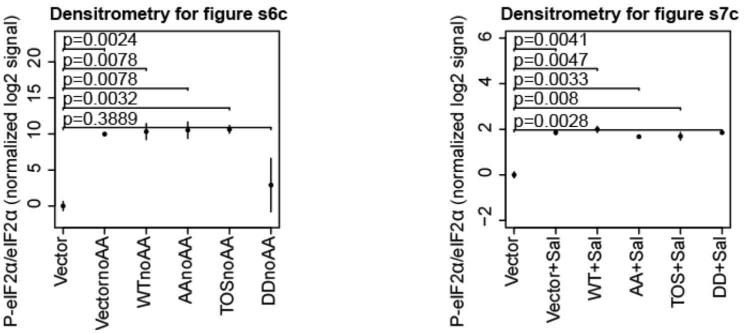

Supp. Fig. 9. Densitometry for Western blotting and related statistical analysis. Shown are log2 transformed and normalized (per replicate and to the mean of a selected “control” under each experiment) densitometry signals (as determined by ImageJ) for the indicated figures and protein ratios. P-values from 1-way ANOVAs are indicated. Western blotting experiments were repeated at least in independent duplicates.

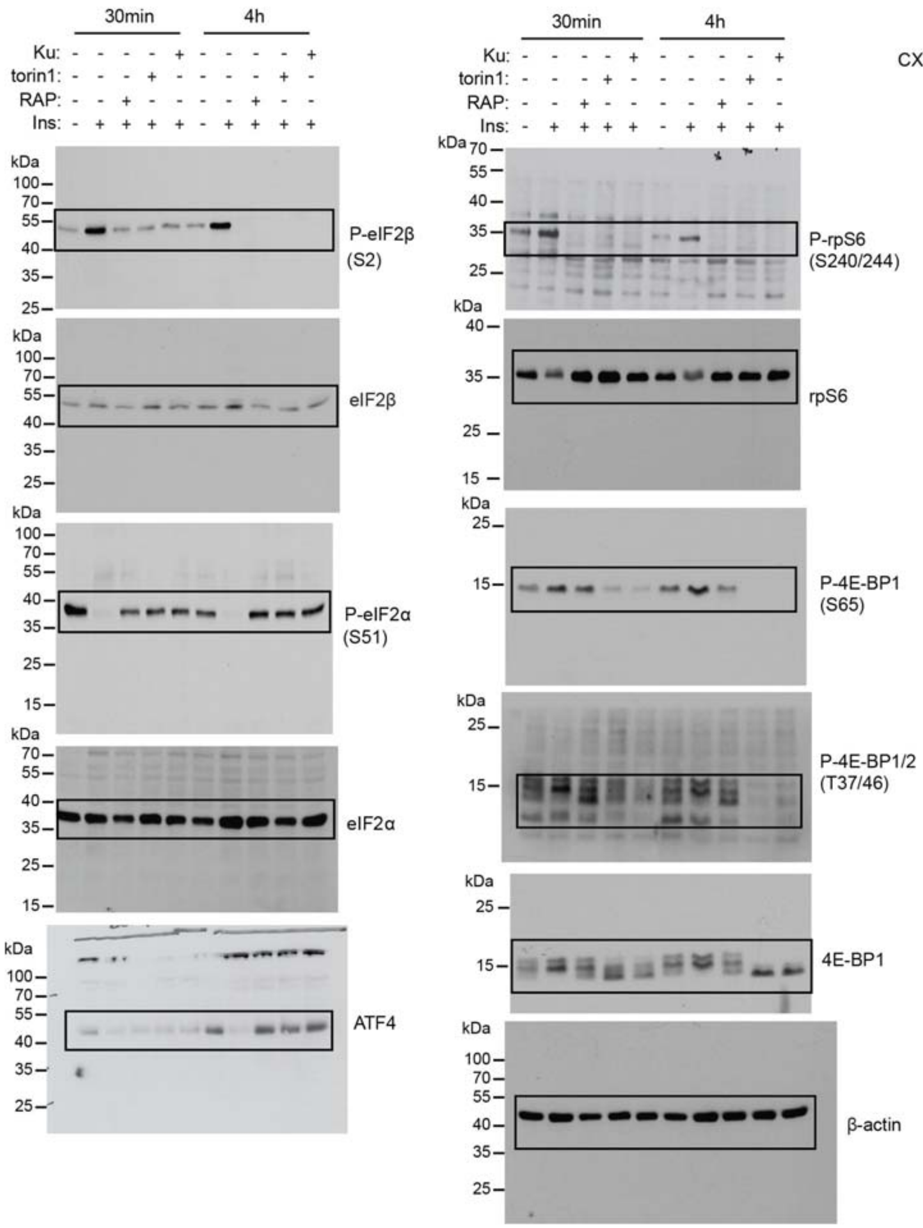

Fig.1c

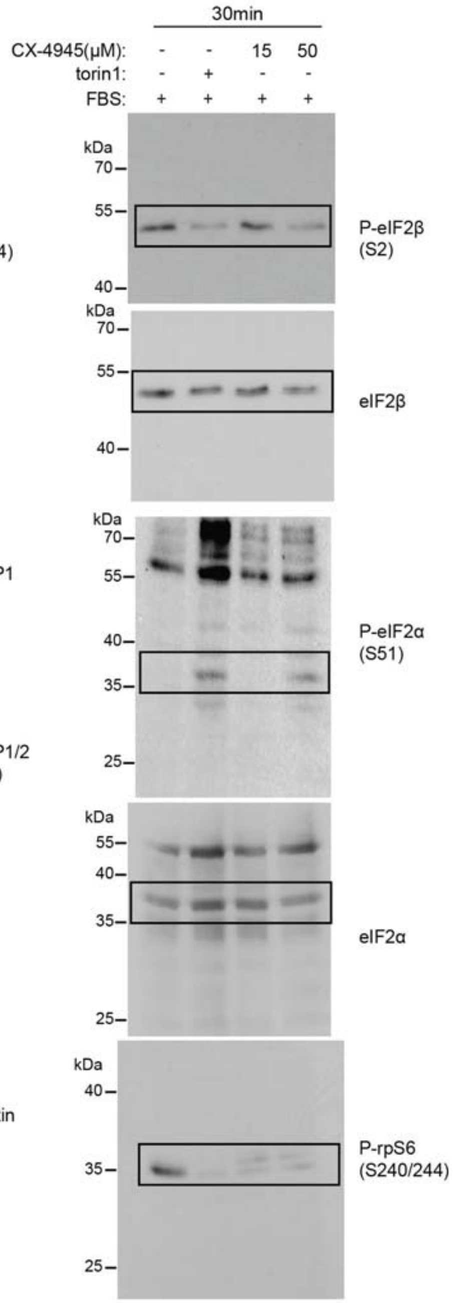

Fig 2b

Supplementary Fig. 10 (continued below; legend on page 32)

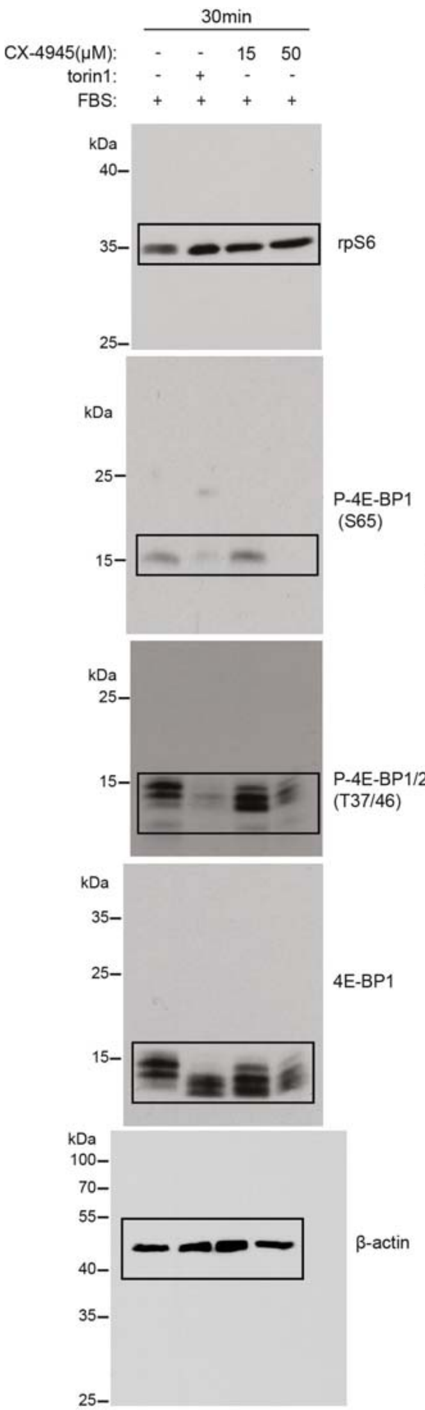

Fig 2b.Cont

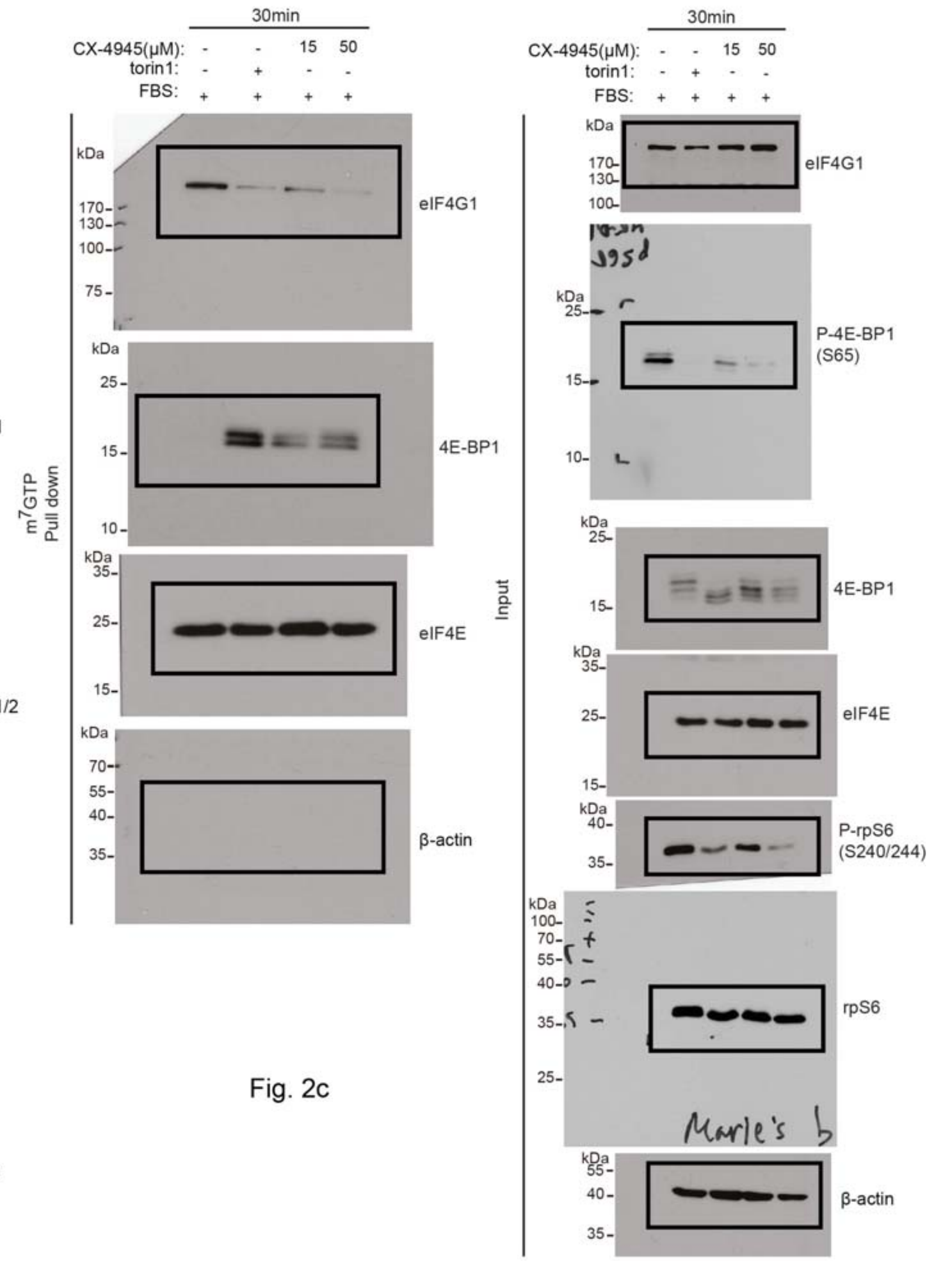

Fig. 2c

Supplementary Fig. 10 (continued below; legend on page 32)

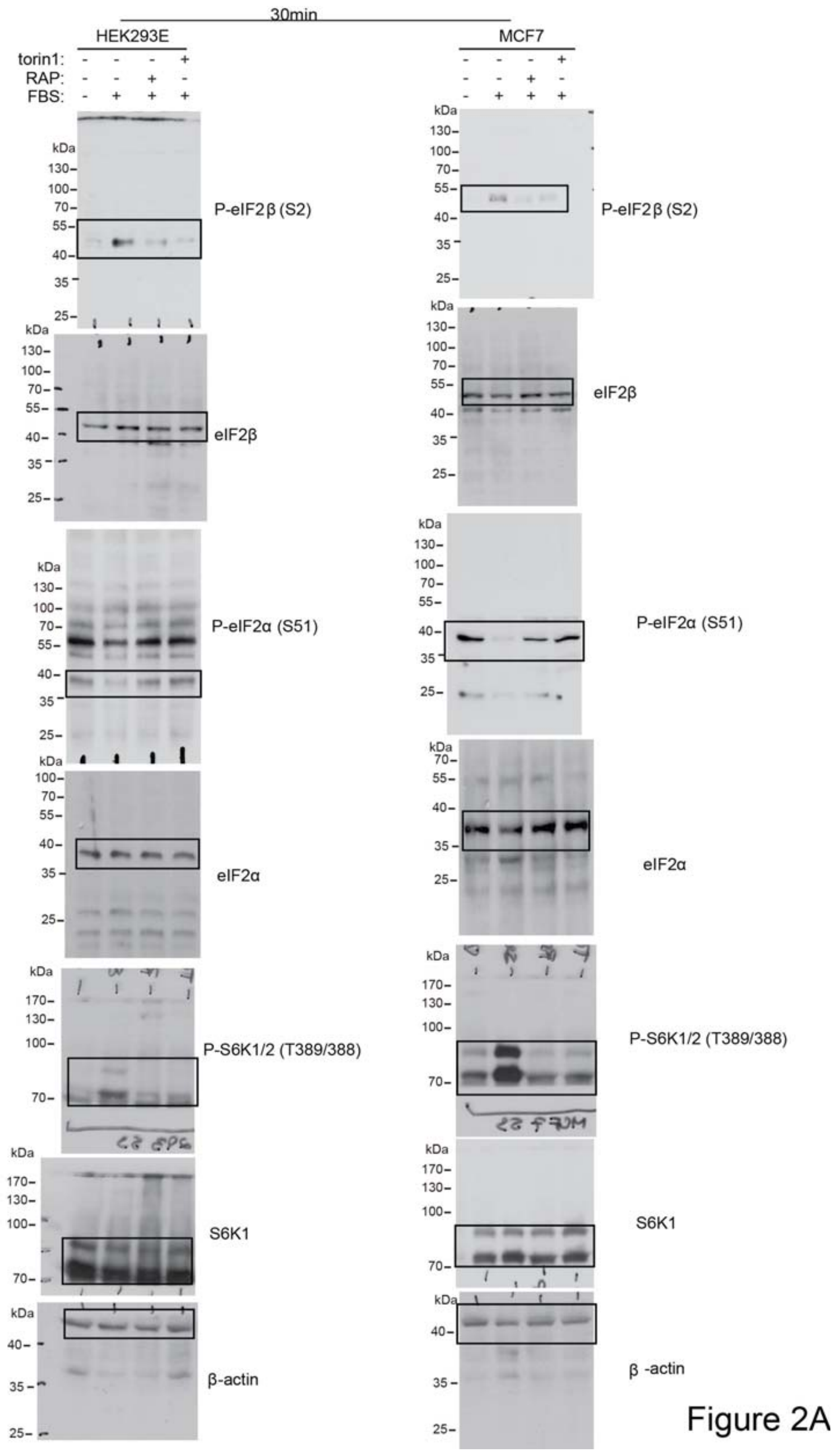

Supplementary Fig. 10 (continued below; legend on page 32)

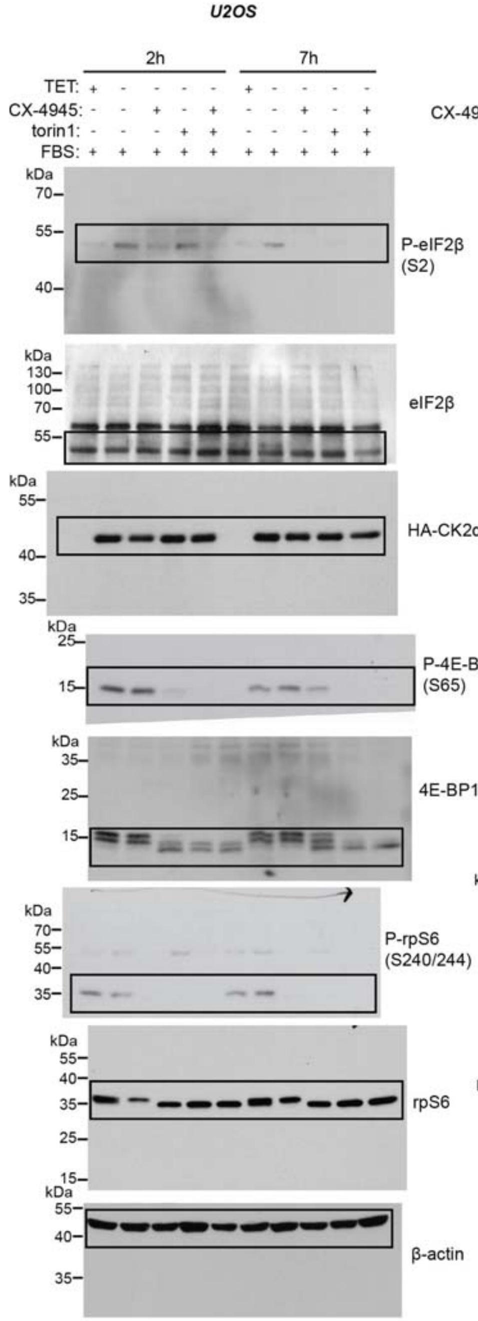

Fig. 2d

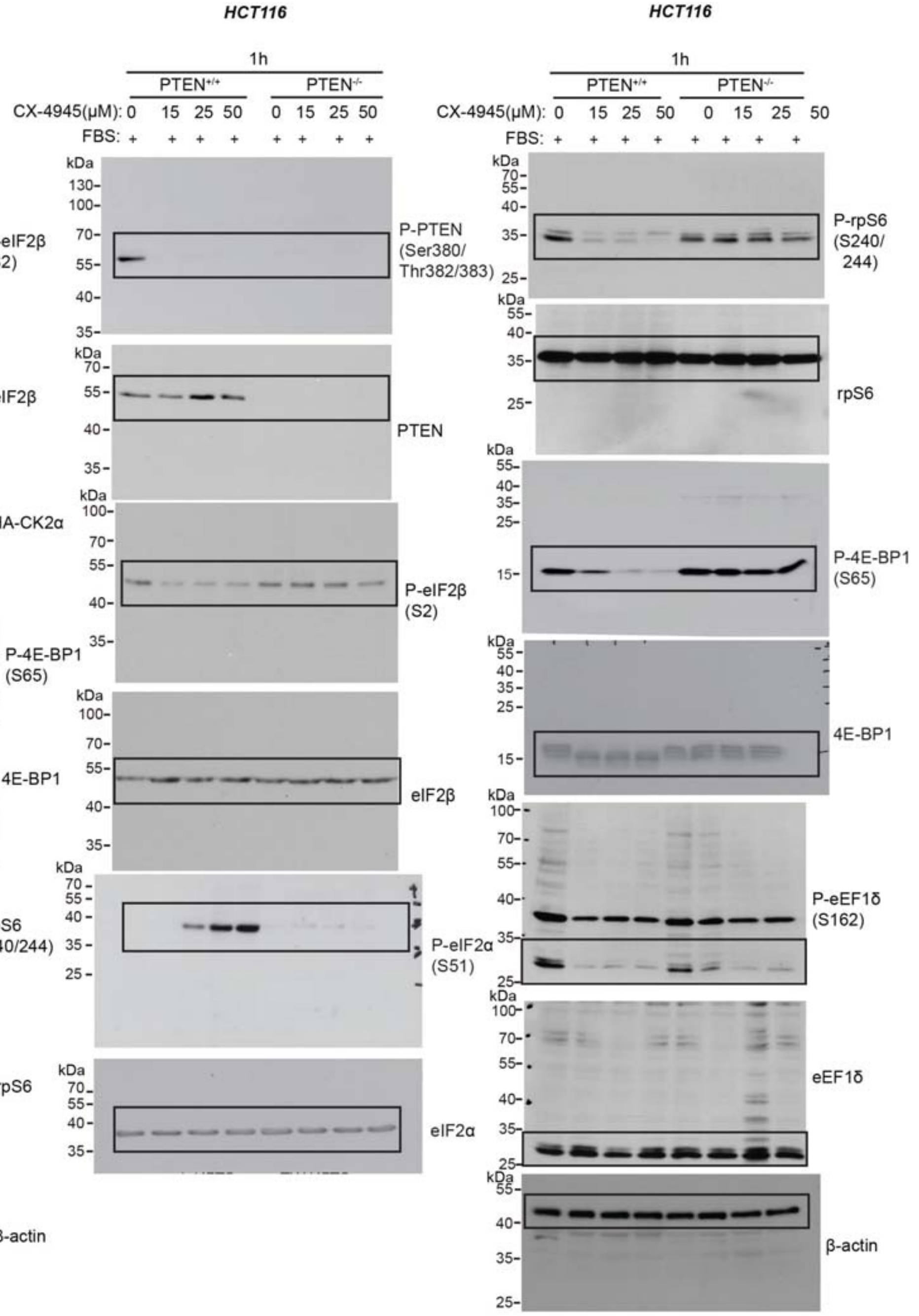

Fig. 2e

Supplementary Fig. 10 (continued below; legend on page 32)

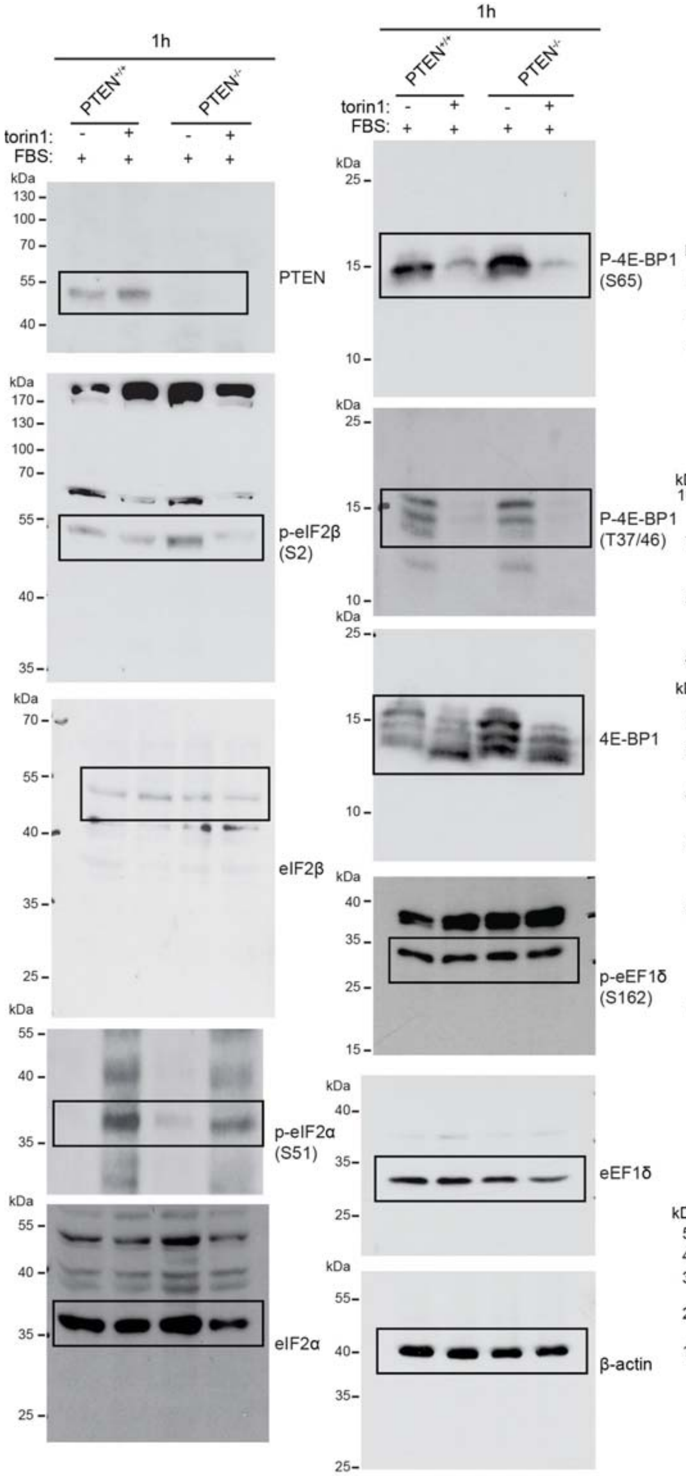
1h
PTEN+/+
PTEN-/-
torin1:
FBS:
kDa
PTEN
p-eIF2β (S2)
eIF2β
p-eIF2α (S51)
eIF2α
P-4E-BP1 (S65)
P-4E-BP1 (T37/46)
4E-BP1
p-eEF1δ (S162)
eEF1δ
β-actin

Fig. 2f

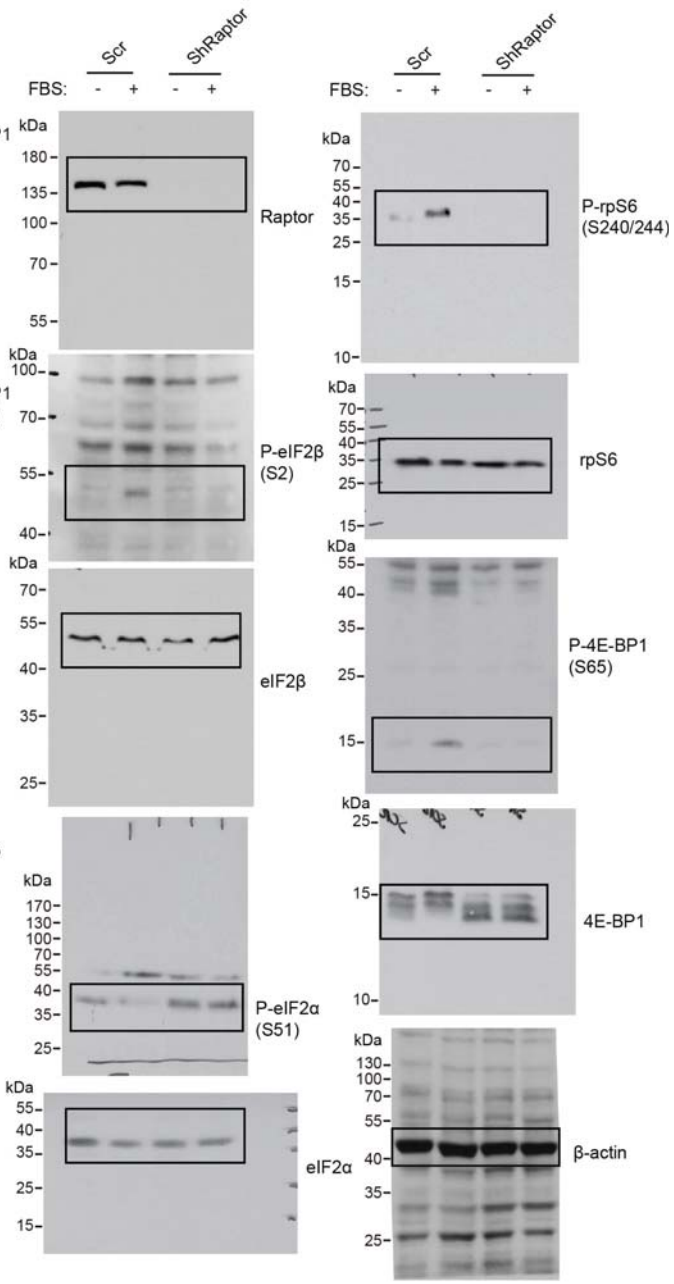
Scr
ShRaptor
FBS:
kDa
Raptor
P-eIF2β (S2)
eIF2β
P-eIF2α (S51)
eIF2α
P-rpS6 (S240/244)
rpS6
P-4E-BP1 (S65)
4E-BP1
β-actin

Fig. 3a

Supplementary Fig. 10 (continued below; legend on page 32)

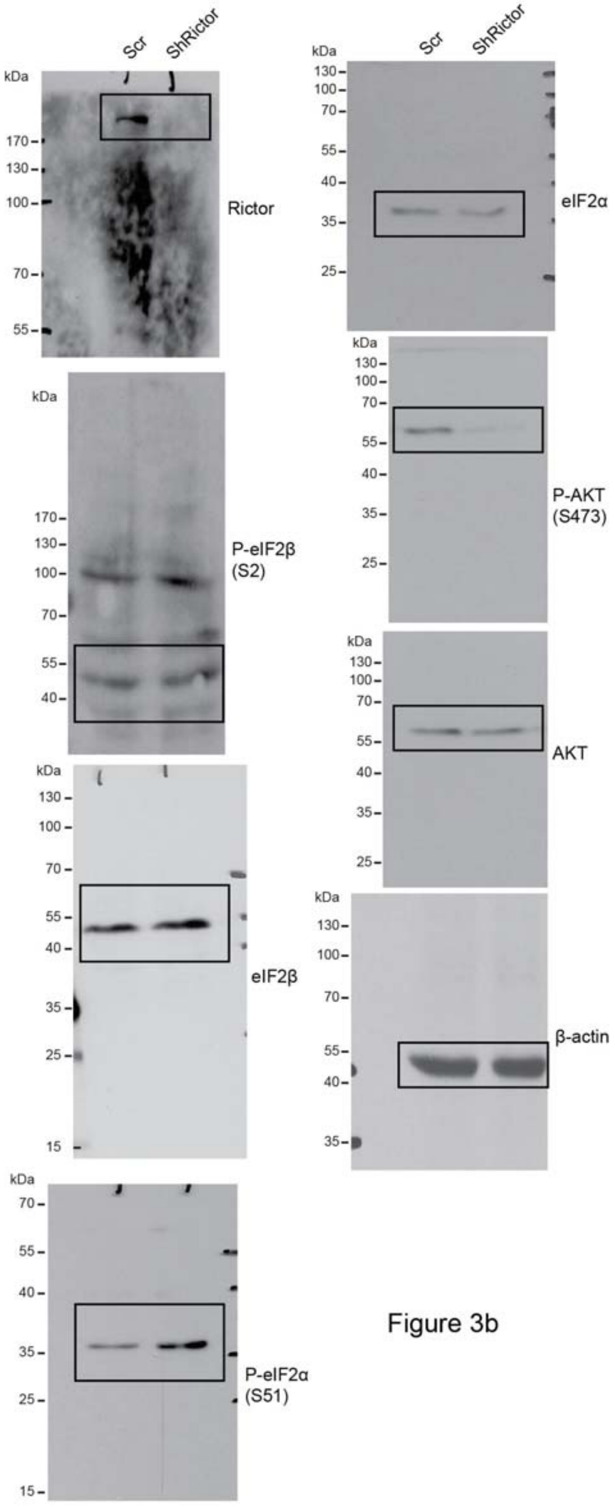

Figure 3b

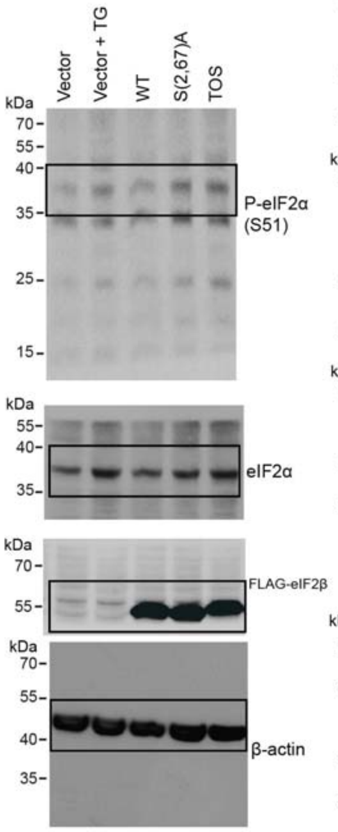

Figure 3c

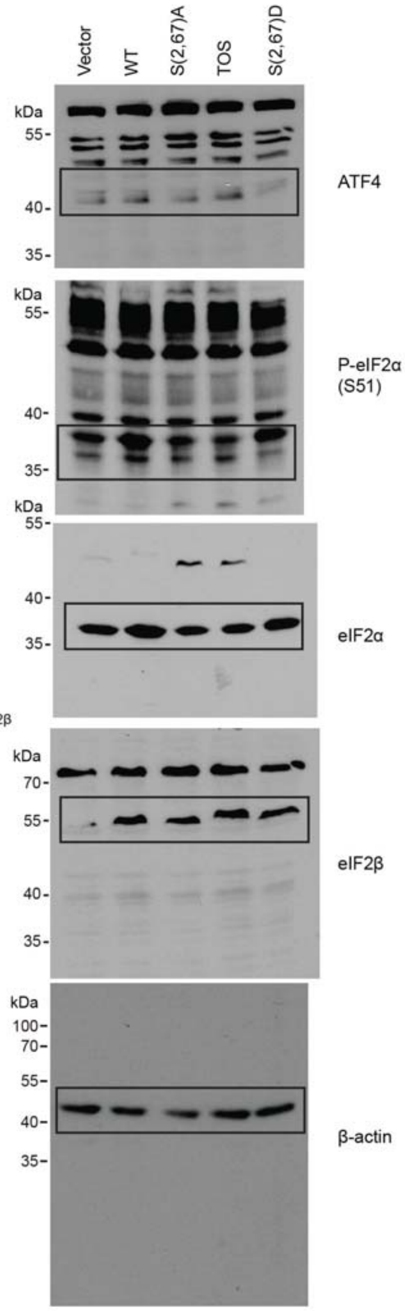

Figure 3d

Supplementary Fig. 10 (continued below; legend on page 32)

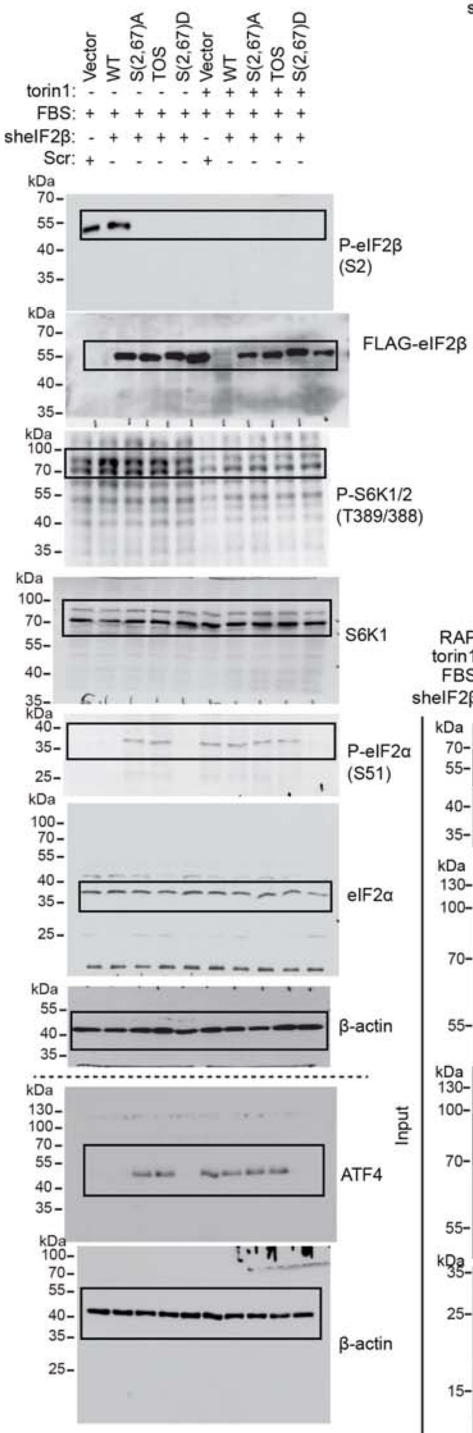

Fig. 3g

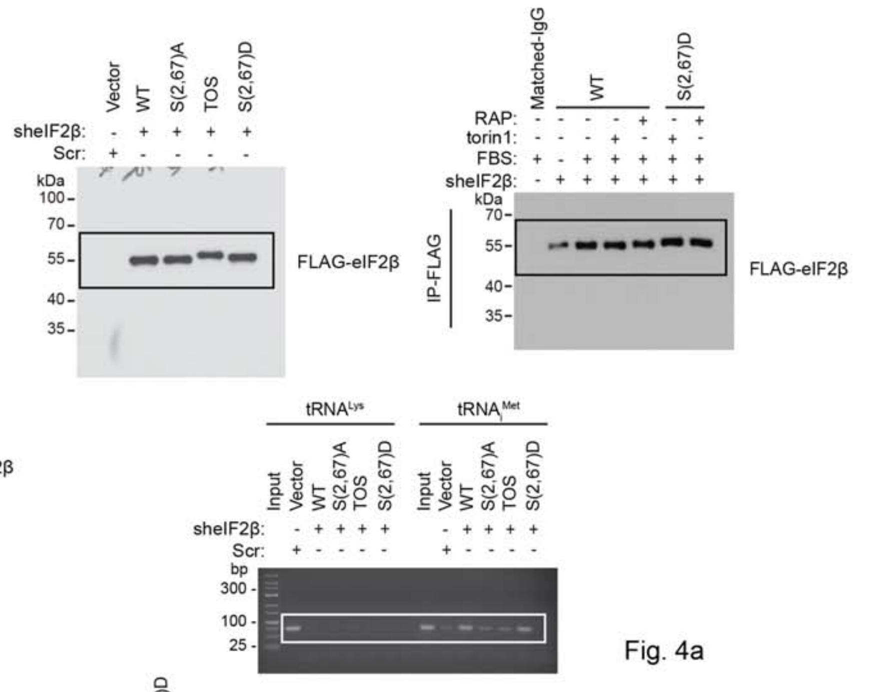

Fig. 4a

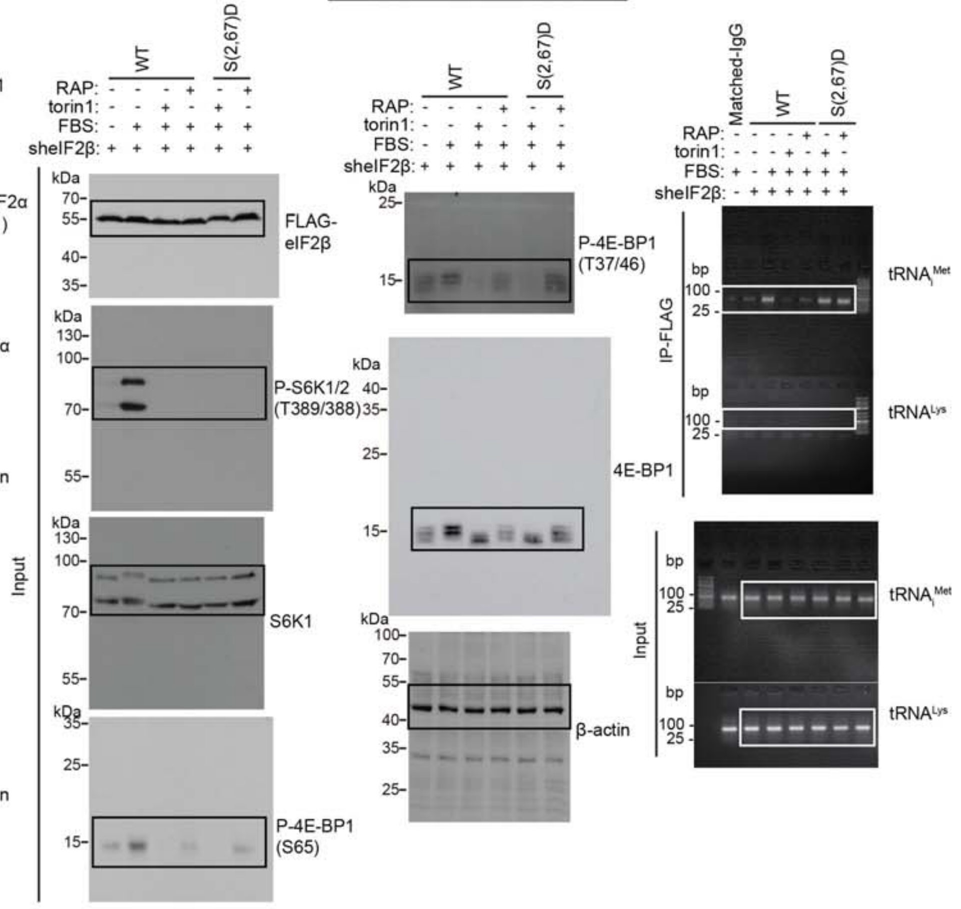

Fig. 4b

Supplementary Fig. 10 (continued below; legend on page 32)

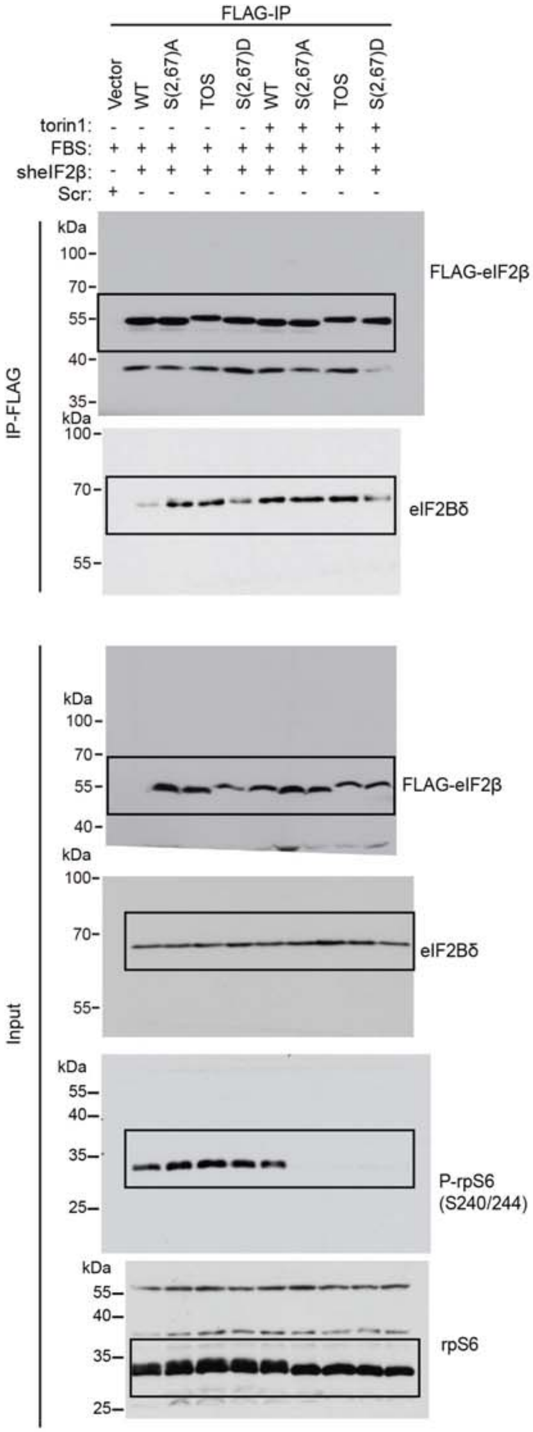

Fig. 4c

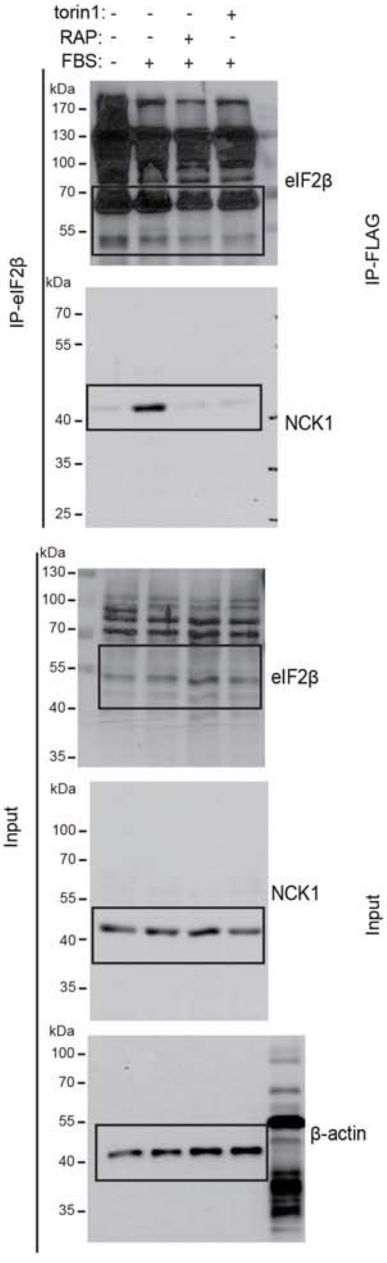

Fig. 5a

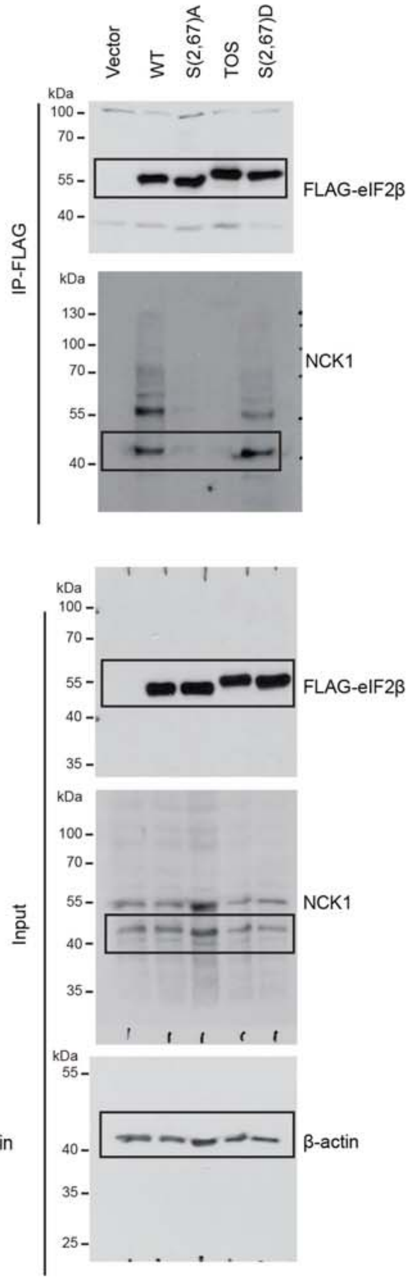

Fig. 5b

Supplementary Fig. 10 (continued below; legend on page 32)

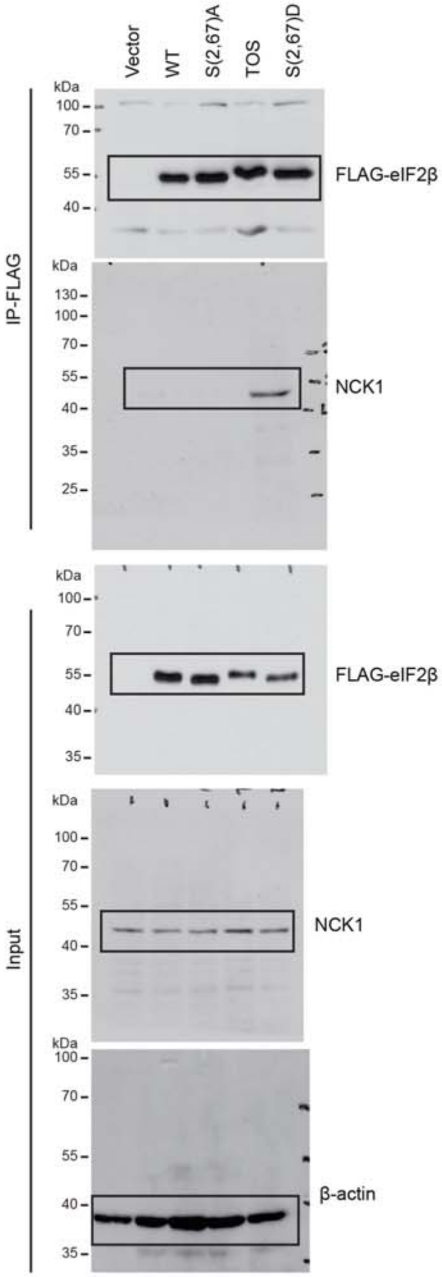

Fig. 5c

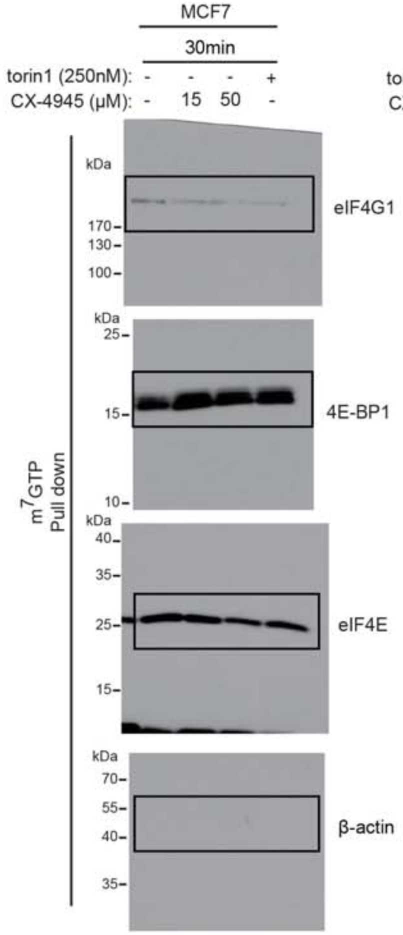

Fig. S1a

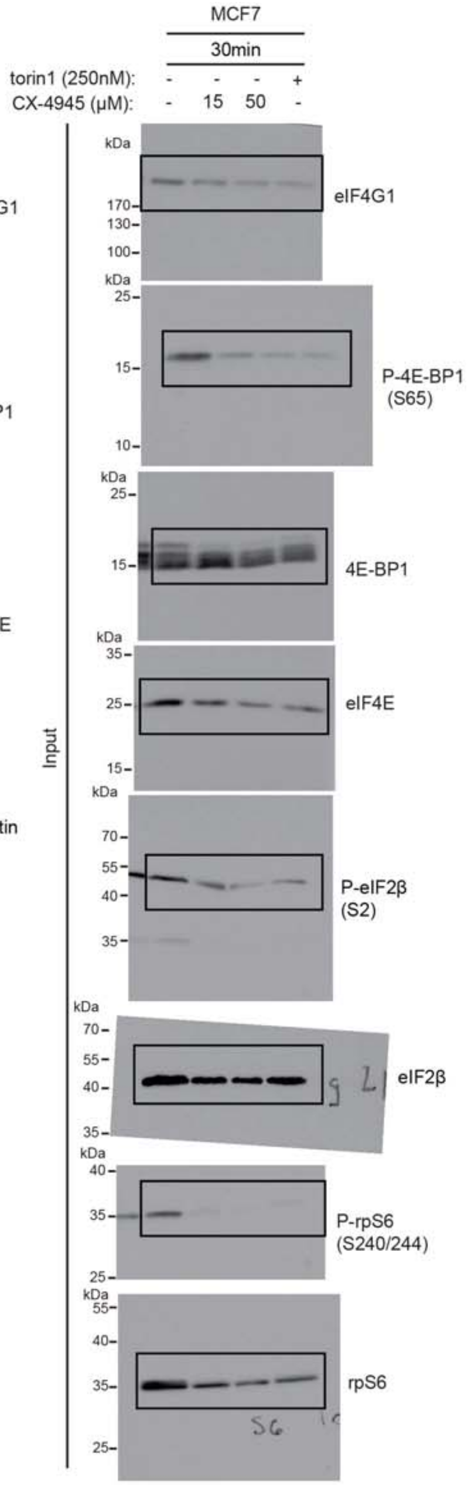

Fig. S1a

Supplementary Fig. 10 (continued below; legend on page 32)

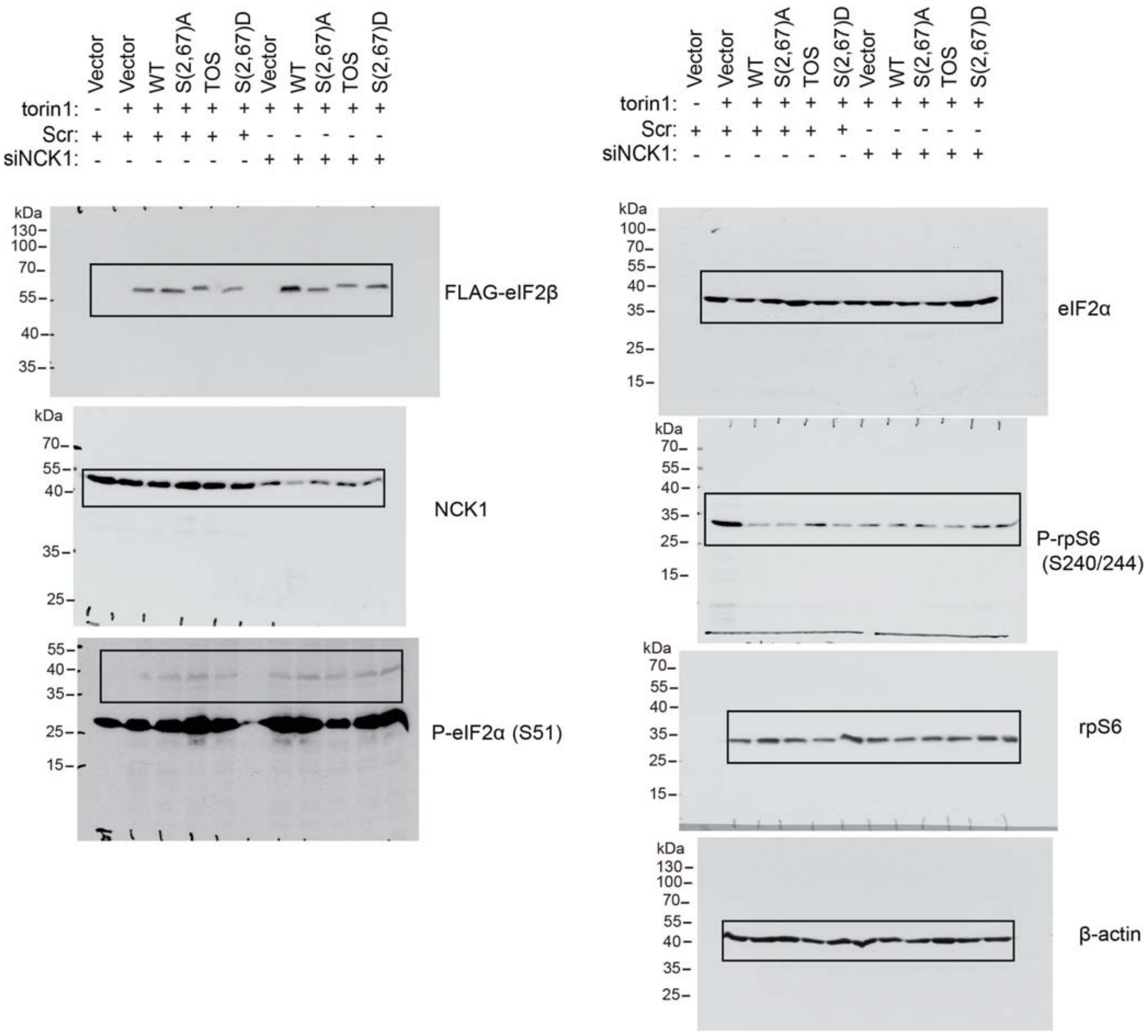

Figure 5d

Supplementary Fig. 10 (continued below; legend on page 32)

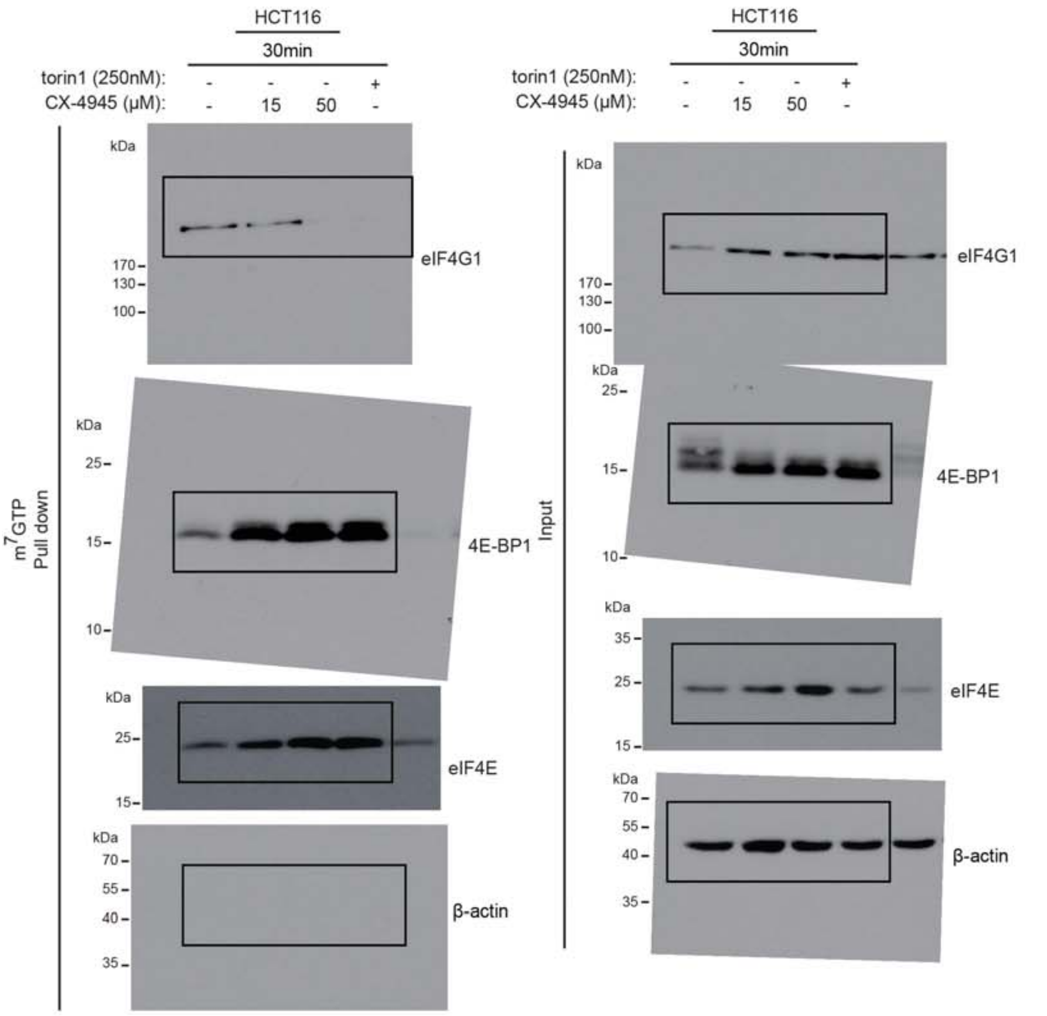

Fig. S1b

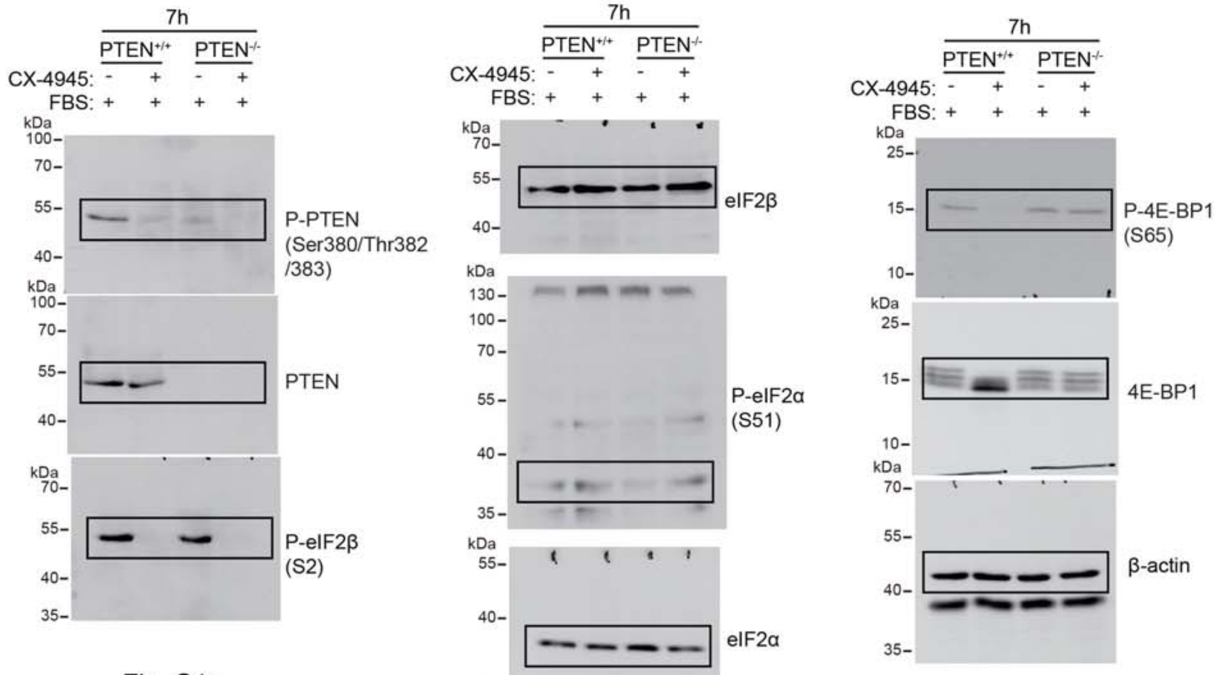

Fig. S1c

Supplementary Fig. 10 (continued below; legend on page 32)

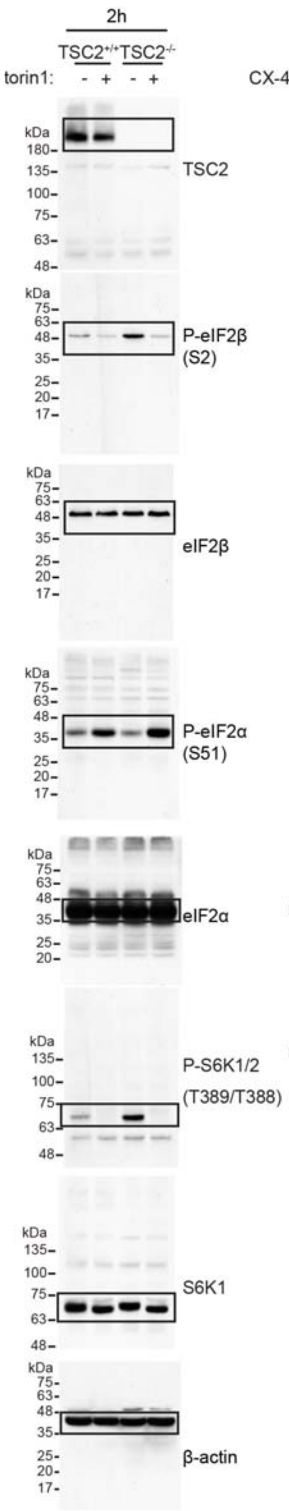

Fig. S1d

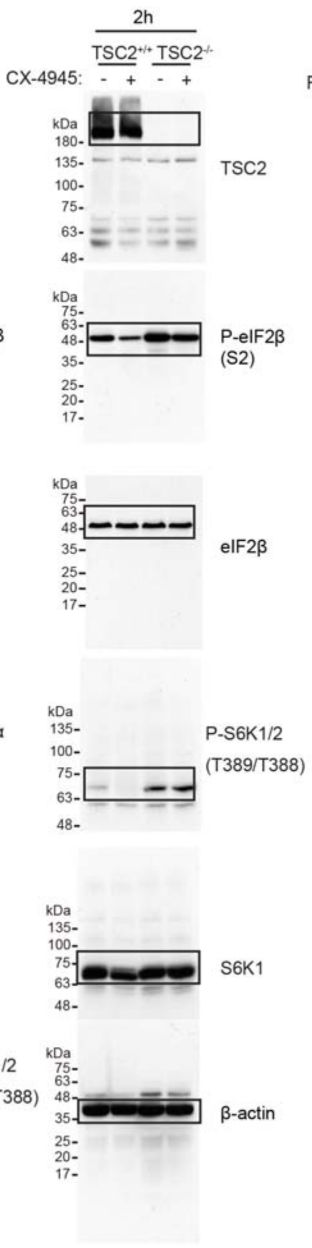

Fig. S1e

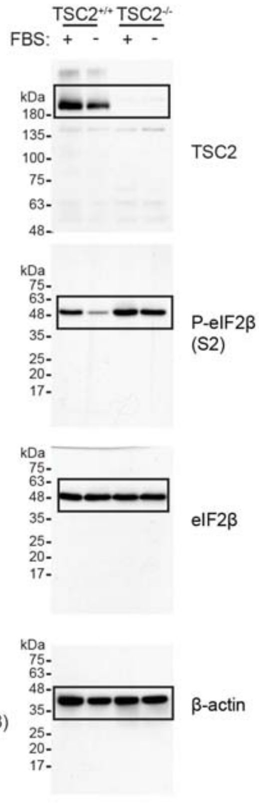

Fig. S1f

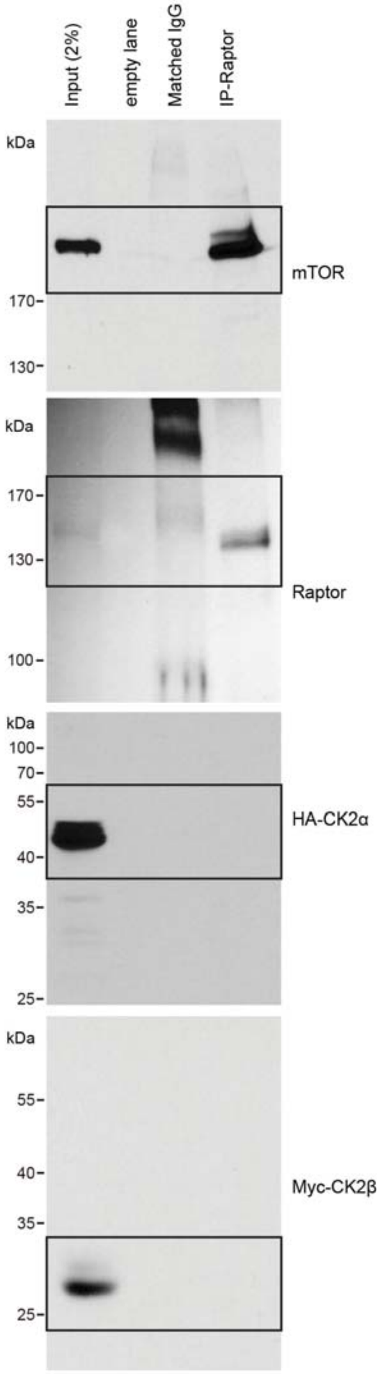

Fig. S2g

Supplementary Fig. 10 (continued below; legend on page 32)

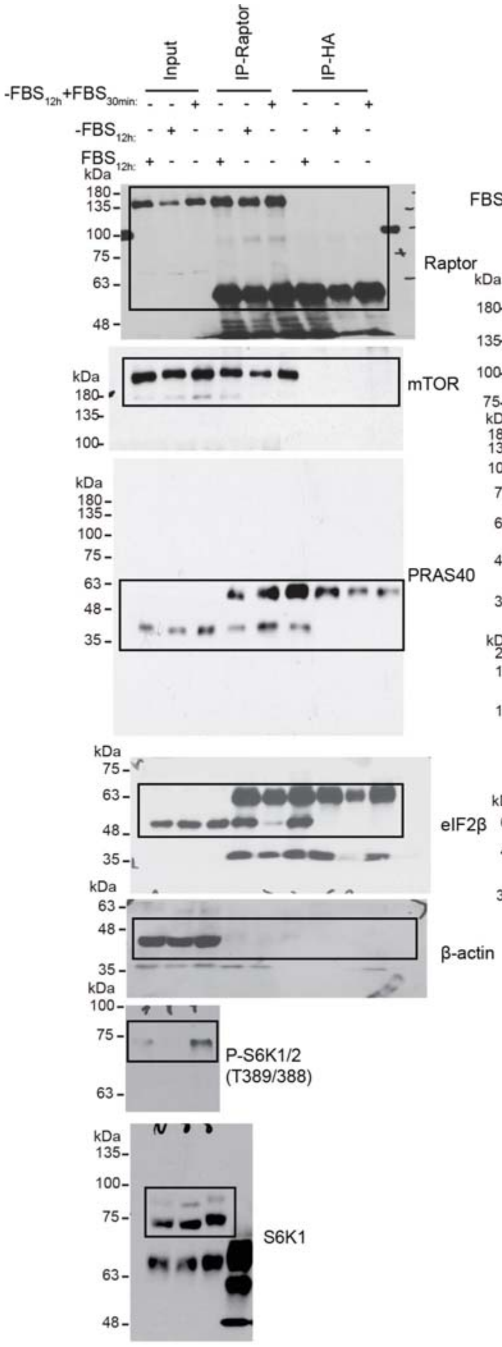

Fig. S2h

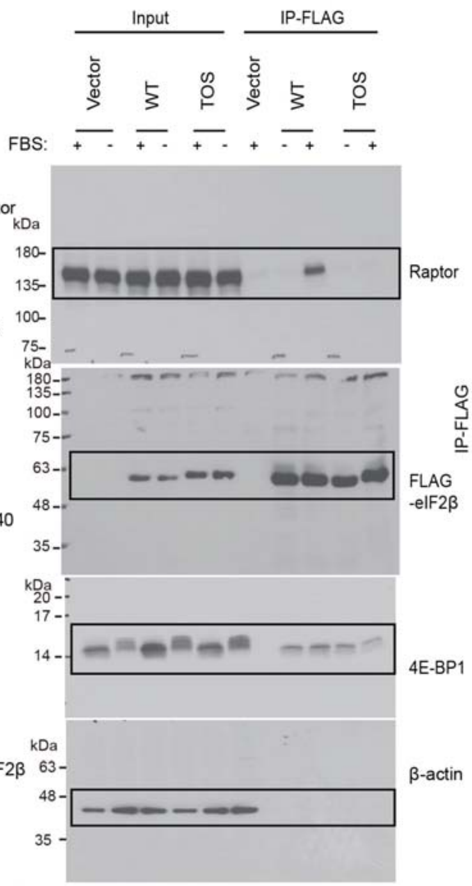

Fig. S2i

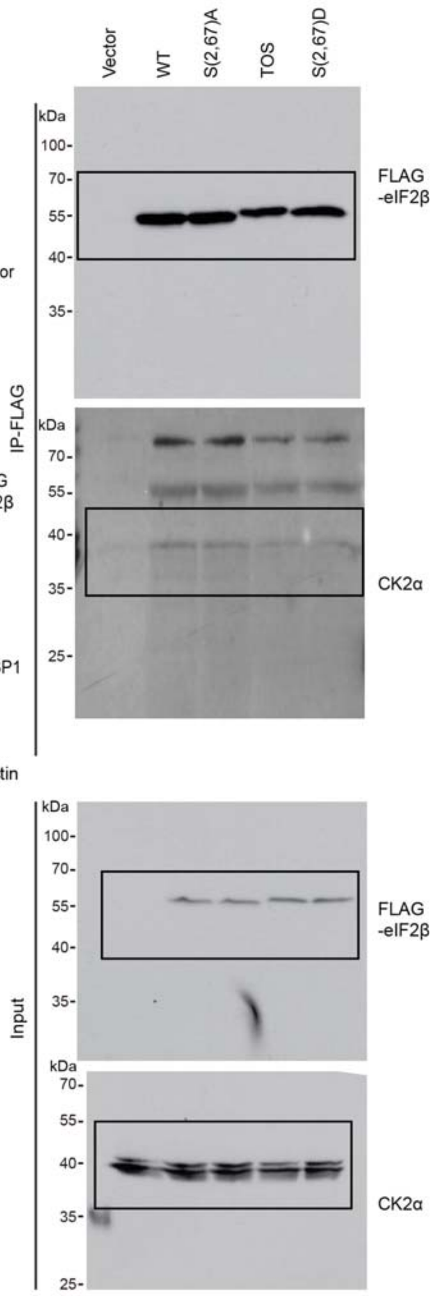

Fig.S2j

Supplementary Fig. 10 (continued below; legend on page 32)

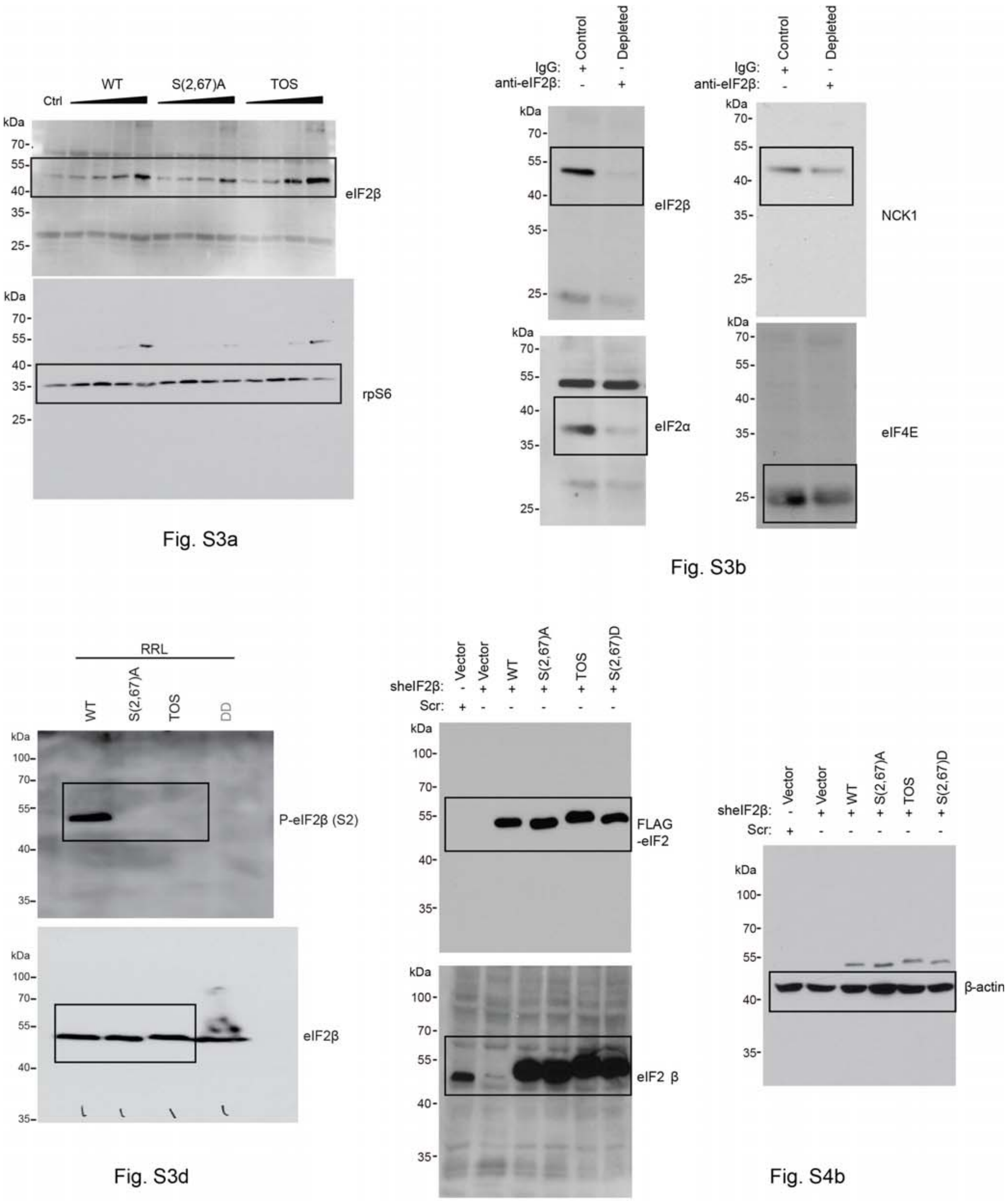

Supplementary Fig. 10 (continued below; legend on page 32)

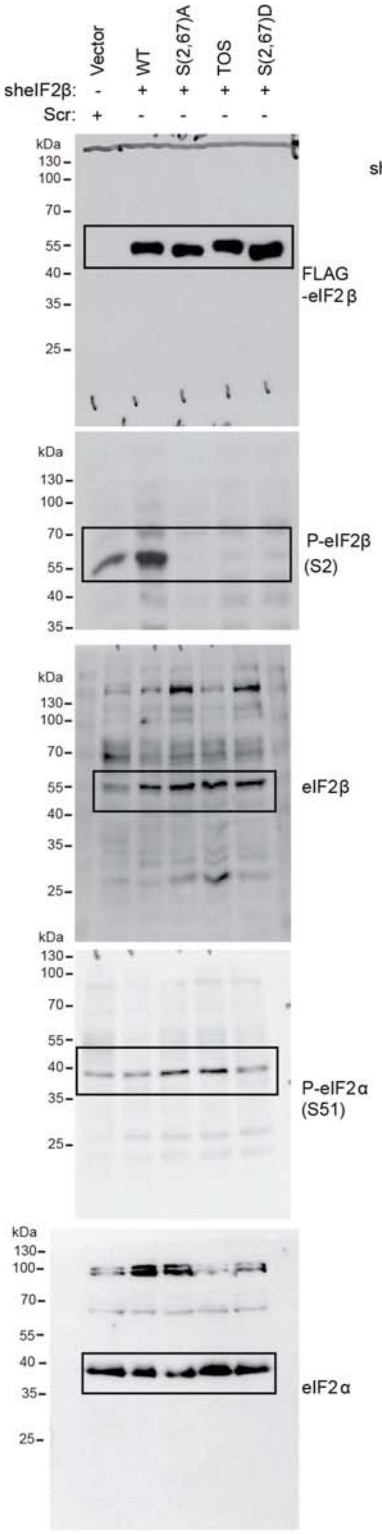

Fig. S4c

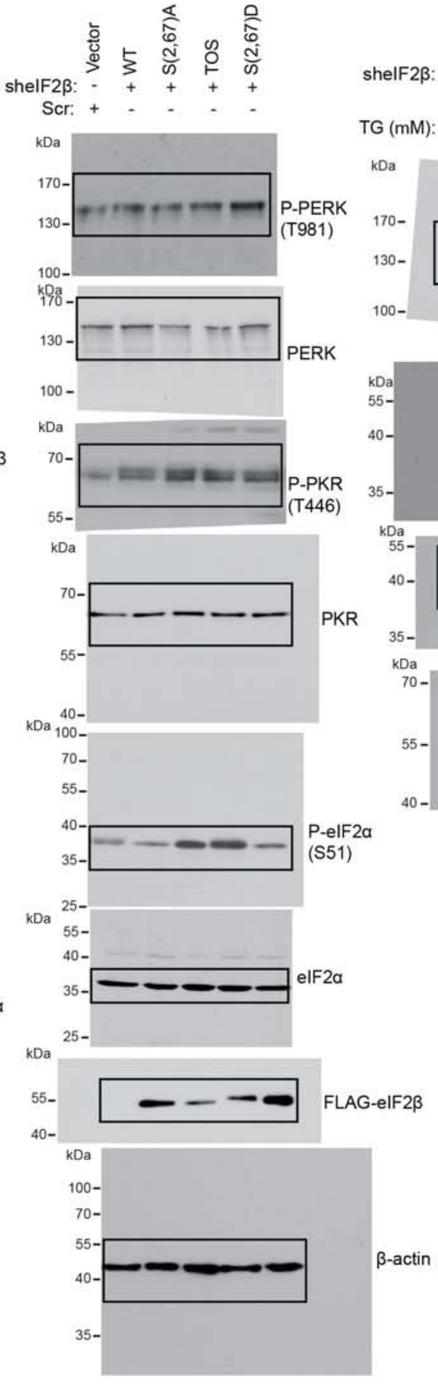

Fig. S5A

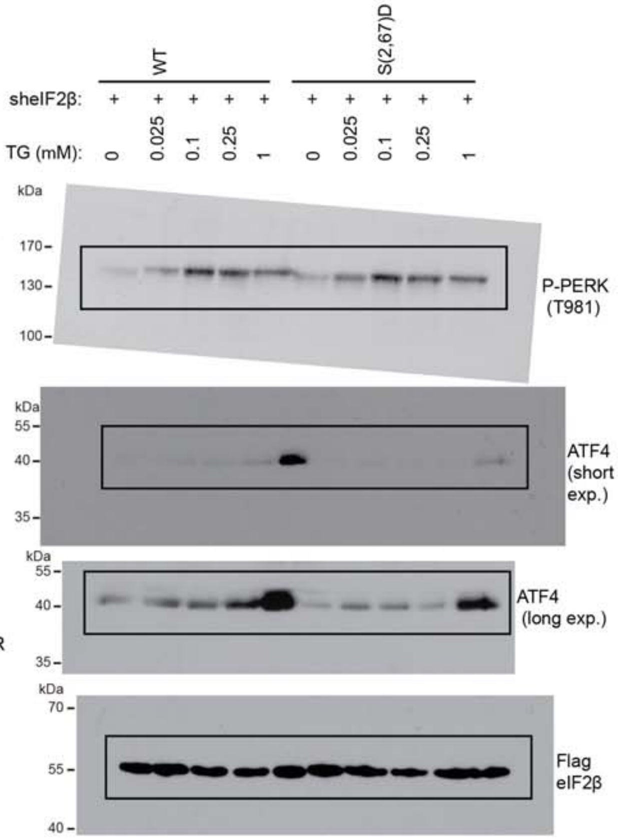

Fig. S5b

Supplementary Fig. 10 (continued below; legend on page 32)

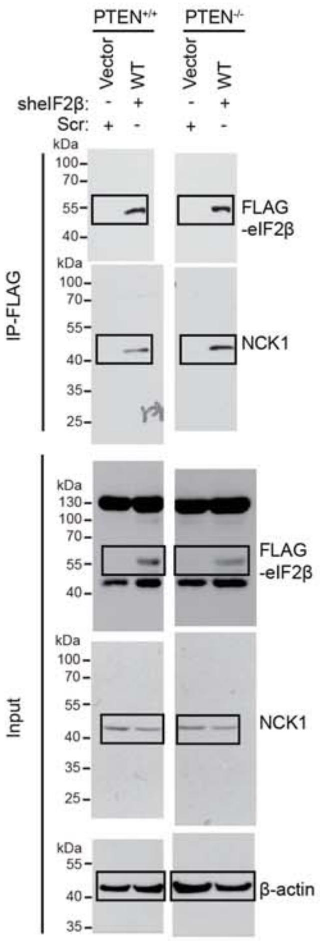

Fig. S5c

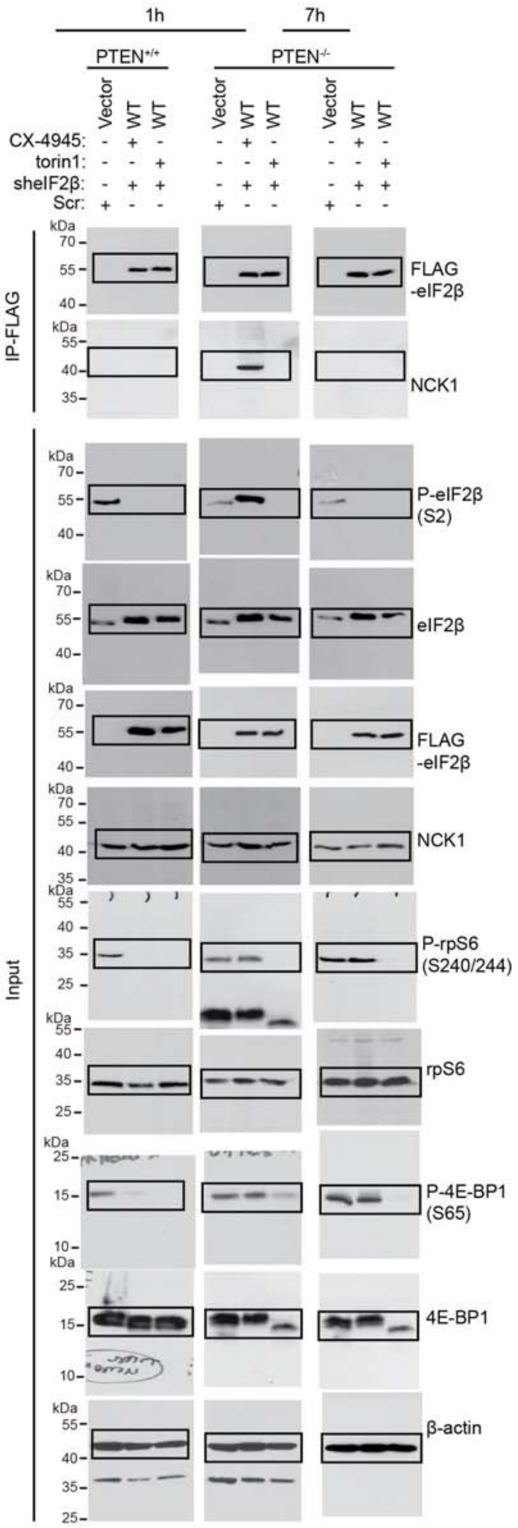

Fig. S5d

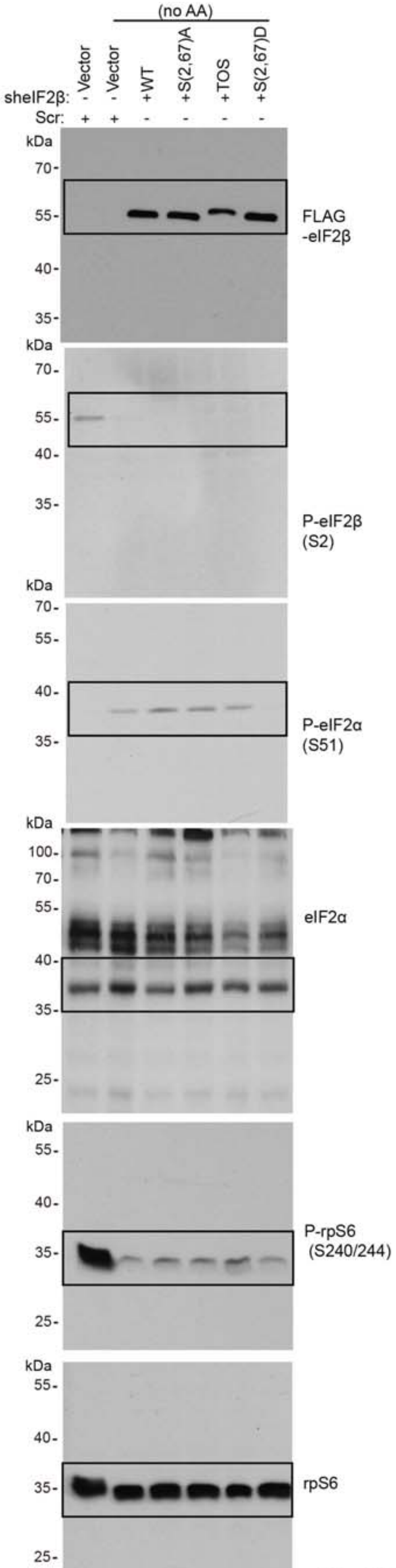

Fig. S6c

Supplementary Fig. 10 (continued below; legend on page 32)

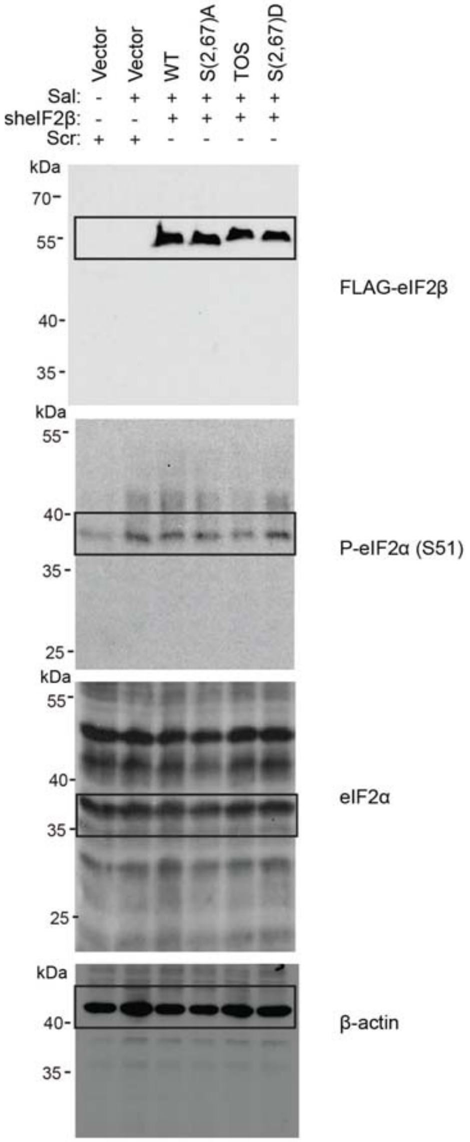

Figure S7C

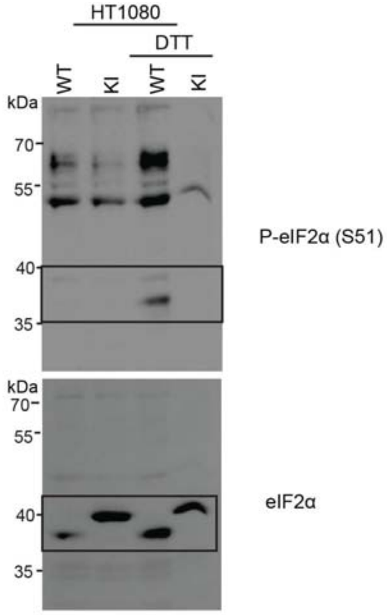

Figure S8B

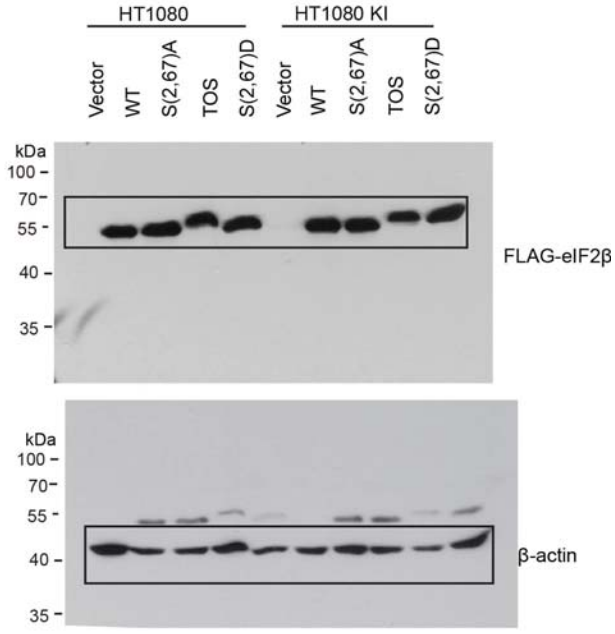

Figure S8C

Supplementary Fig. 10 Scans of the uncropped films and images of agarose gels that were used in the manuscript. Boxes indicate approximate portions of the films that are included in the figures. Films were re-scanned to obtain larger area around the bands, therefore causing slight differences in the contrast. Below the scans it is indicated to which figure they correspond.
